# Supplementary material for: Thermally-induced atropisomerism promotes metal-organic cage construction
Source: Nat Commun. 2023 Dec 9;14:8166. doi: 10.1038/s41467-023-43756-4 (PMC10710450; doi:10.1038/s41467-023-43756-4)

Supplementary Information For:

Thermally-induced atropisomerism promotes metal-organic cage construction

Jiaqi Liang,[1] Shuai Lu,[2] Yang Yang,[1] Yun-Jia Shen,[1] Jin-Ku Bai,[1] Xin Sun,[1] Xu-Lang Chen,[3] Jie Cui,[4] Ai-Jiao Guan,[4] Jun-Feng Xiang,[4] Xiaopeng Li,[2] Heng Wang,[2]* Yu-Dong Yang,[1,5]* Han-Yuan Gong[1]*

[1]College of Chemistry, Beijing Normal University, Xinjiekouwaidajie 19, Beijing 100875, P. R. China.

[2]College of Chemistry and Environmental Engineering, Shenzhen University, Shenzhen, Guangdong 518060, P. R. China.

[3]College of Chemistry and Chemical Engineering, Hubei Key Laboratory of Pollutant Analysis and Reuse Technology, Hubei Normal University, Huangshi 435002, P. R. China.

[4]Institute of Chemistry Chinese Academy of Sciences, Beijing 100190, P. R. China.

[5]Department of Chemistry, The University of Texas at Austin, 105 East 24th Street, Stop A5300, Austin, Texas 78712-1224, United States.

Author e-mail address: hanyuangong@bnu.edu.cn

Supplementary Note 1: General considerations

Supplementary Note 2: NMR and Mass spectra of the compounds **1-9**.

Supplementary Note 3: Theoretical formation energy calculation of cyclization intermediates (**1-im, 2-im**, and **3-im**).

Supplementary Note 4: X-ray crystallography of single crystal structures **1**•2.25CH_2Cl_2, **2**•CH_2Cl_2•0.75H_2O and **3**•$CH_3COOC_2H_5$•2CH_3CN•3.5H_2O.

Supplementary Note 5: Thermally induced transformation processes between **1**-**3**.

Supplementary Note 6: ^{1}H NMR spectra of thermally induced interconversion processes between **1** and **2** enantiomers.

Supplementary Note 7: ^{1}H NMR spectra of thermally induced interconversion processes from **1** or **2** to **3**.

Supplementary Note 8: Theoretical calculation of the conformation transformation process from **1** to **3**.

Supplementary Note 9: X-ray crystallography of single crystal structure **4**.

Supplementary Note 10: ^{1}H NMR spectroscopic titration of **1**, or **2** with **$(PhCN)_2PdCl_2$**.

Supplementary Note 11: Characterization of gel containing **1** or **2** and **Pd^{2+}**.

Supplementary Note 12: Optimized geometries of possible complexes among **1, 2, or 3** and Pd^{2+} and their corresponding minimized binding energies (ΔE) as calculated in vacuum.

Supplementary Note 13: ^{1}H-, ^{1}H DOSY NMR spectroscopic of **1, 2, or 3@3Pd**.

Supplementary References

Supplementary Note 1: General considerations

1D ^{1}H and ^{13}C NMR were recorded on JEOL-400 spectrometers. Tempreture-dependent ^{1}H and time-dependent ^{1}H were recorded on Bruker AVANCE III 500WB. Diffusion-ordered spectroscopy (^{1}H DOSY)), 2D NMR (Homonuclear chemical shift correlation spectroscopy (COSY), and Nuclear Överhauser effect spectroscopy (NOESY)) were recorded on the AVANCE 600 spectrometer. ^{1}H and ^{13}C NMR chemical shifts are referenced to residual solvent signals ($CDCl_3$: δ_H = 7.26 ppm, δ_C = 77.16 ppm; tetrachloroethane-d_2 (TCE-d_2): δ_H = 5.95 ppm, δ_C = 74.10 ppm, nitrobenzene-d_5 ($PhNO_2$-d_5): δ_H = 8.11 ppm, 7.67 ppm, 7.50 ppm, δ_C = 148.6 ppm, 134.8 ppm, 129.5 ppm, 123.5 ppm, dimethyl sulfoxide-d_6 (DMSO-d_6): δ_H = 2.50 ppm, δ_C = 39.6 ppm). High-resolution mass spectra (HMRS) were detected on Bruker Solarix FT-ICR-MS (ESI, EI) or Bruker auto flex speed MALDI-TOF. Different drift times of the three macrocycles were detected on traveling-wave ion mobility mass spectrometry (TWIM-MS and tandem mass spectra (gMS^2). All spectra were collected on a Waters Synapt G2-Si mass spectrometer (Waters Corp., MA, USA). All samples were dissolved in dichloromethane at a concentration of ca. 0.01 mg/mL. The TWIM-MS and gMS^2 experiments were performed under the following conditions: ESI capillary voltage, 3-5 kV; sample cone voltage, 30 V; source temperature 100 ºC; desolvation temperature, 100 ºC; cone gas flow, 100 L/h; desolvation gas flow, 800 L/h (N_2); source gas control, 30 mL/min; trap collision energy, 0–150 kV; transfer collision energy, 4 kV; trap gas flow, 2 mL/min; helium cell gas flow, 180 mL/min; IMS gas flow, 30 mL/min; sample flow rate, 10 µL/min; IMS wave velocity, 700 m/s; IMS wave height, 32 V. The molecular weight of the polymer was determined by gel permeation chromatography (GPC) equipped with a Waters model 1515 pump and a model 2414 differential refractometer. Scanning electron microscopy (SEM) images were obtained by a Hitachi S-4800 (Tokyo, Japan). Dynamic light scattering (DLS) was recorded on a Brookhaven Zeta Plus Particle Size and Zeta Potential Analyzer (Midland, Canada). The viscosity of the mixed solvent was measured using a Ubbelohde viscosimeter with the viscosity of water as standard. Powder X-ray diffraction (PXRD) studies were

carried out using a Shimadzu XRD7000 setup.

Single crystals used to obtain the X-ray diffraction structures grew as a colorless prism, colorless block, or light yellow block. The .cif documents are available as separate supporting information files, and provide details regarding the specific crystals used for the analysis, along with the structures in question. The data crystals used for single crystal analyses were cut from clusters of the corresponding crystals and had the approximate dimensions given in the .cif documents. The data were collected on a Saturn724+ (2 x 2 bin mode) or SuperNova, Dual, Cu at home/near, AtlasS2. Data reduction was performed using CrystalClear or CrysAlisPro software packages. The structures were refined by full–matrix least–squares on F^2 with anisotropic displacement parameters for the non–H atoms using SHELXL-2014 or SHELXL-2018.[1] The hydrogen atoms were calculated in idealized positions with isotropic displacement parameters set to 1.2xUeq of the attached atom (1.5xUeq for methyl hydrogen atoms). Definitions used for calculating R(F), R_w(F2), and the goodness of fit, S, are given below and in the .cif documents.[2] Neutral atom scattering factors and values used to calculate the linear absorption coefficient are from the International Tables for X-ray Crystallography (1992).[3] All ellipsoid figures were generated using SHELXTL/PC.[4] Tables of positional and thermal parameters, bond lengths and angles, torsion angles, figures, and lists of observed and calculated structure factors are located in the .cif documents available from the Cambridge Crystallographic Centre and be shown by quoting ref. numbers shown in Supplementary Table 5-6. The documents also contain details of the crystal data, data collection, and structure refinement for each structure.

Supplementary Note 2: NMR and Mass spectra of the compounds **1-9**.

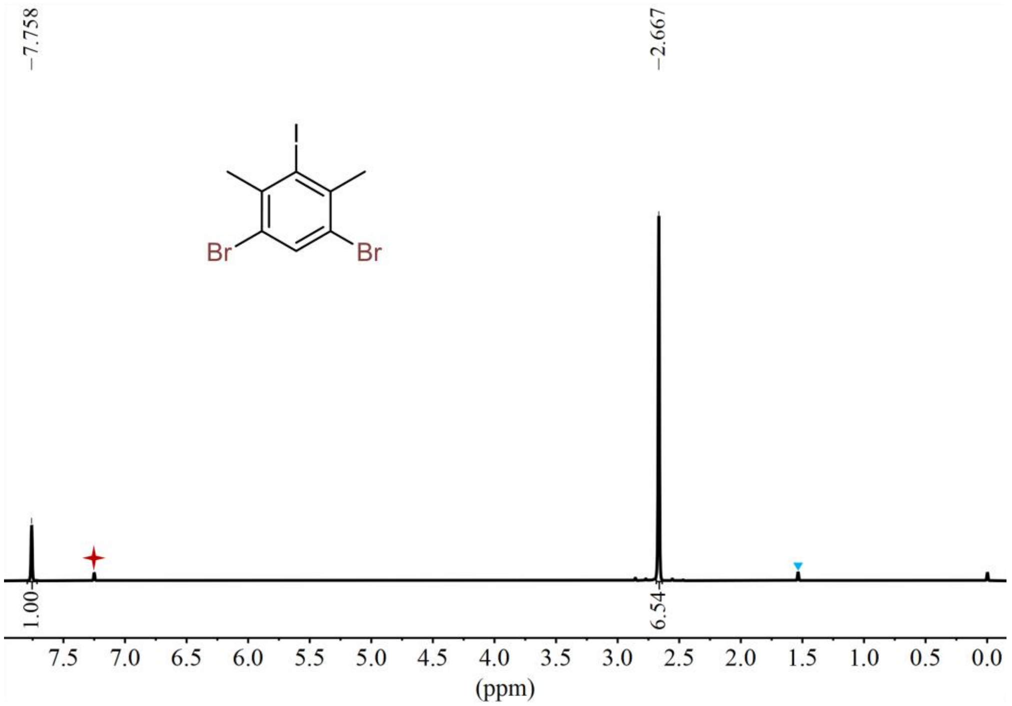

Supplementary Fig. 1 ^{1}H NMR spectrum of **5** (5.00×10^{-2} M) in $CDCl_3$ at 298 K (400 MHz) (red four-pointed star represents residual $CHCl_3$; blue inverted triangle represents H_2O).

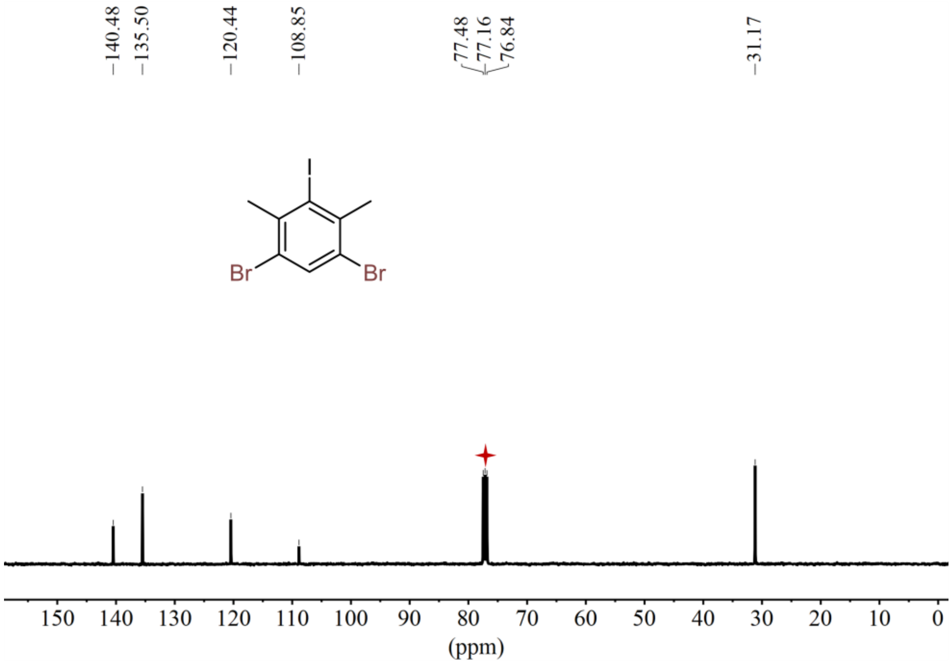

Supplementary Fig. 2 ^{13}C NMR spectrum of **5** (5.00×10^{-2} M) in $CDCl_3$ at 298 K (100 MHz) (red four-pointed star represents residual $CHCl_3$).

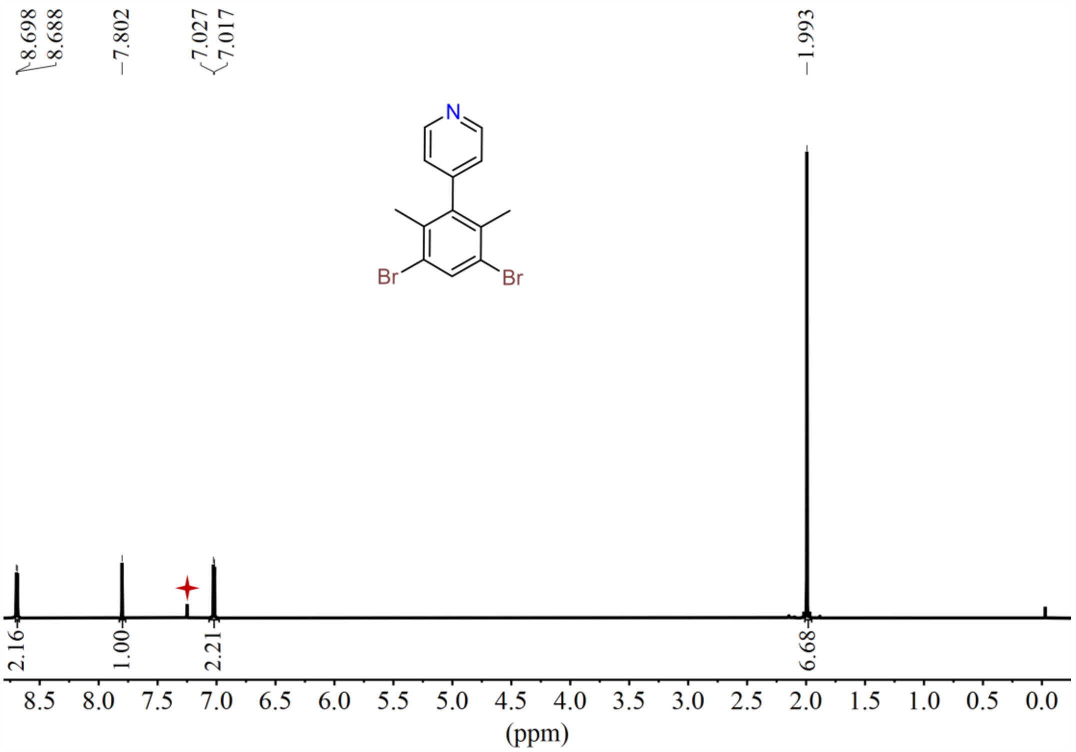

Supplementary Fig. 3 ^{1}H NMR spectrum of **6** (5.00×10^{-2} M) in $CDCl_3$ at 298 K (400 MHz) (red four-pointed star represents residual $CHCl_3$).

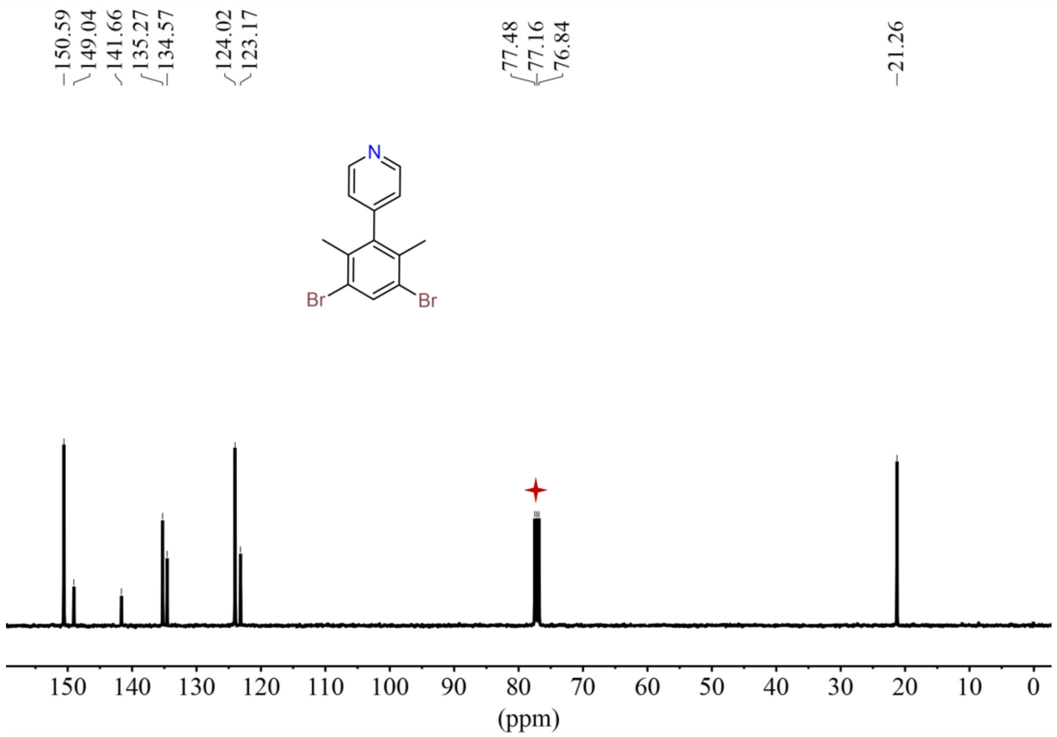

Supplementary Fig. 4 ^{13}C NMR spectrum of **6** (5.00×10^{-2} M) in $CDCl_3$ at 298 K (100 MHz) (red four-pointed star represents residual $CHCl_3$).

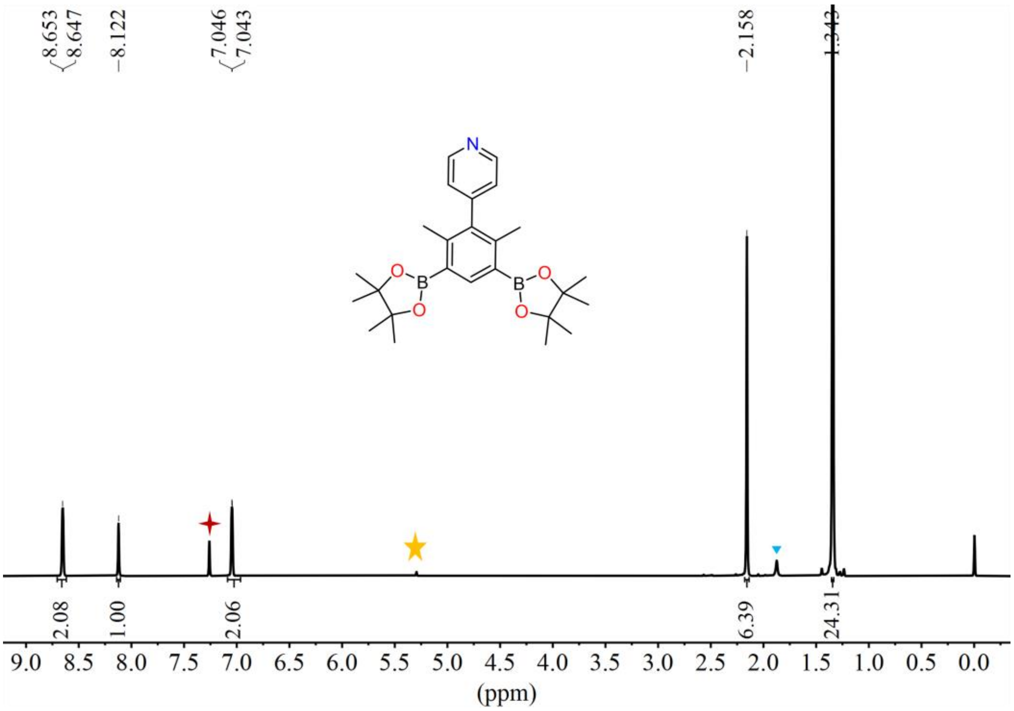

Supplementary Fig. 5 ^{1}H NMR spectrum of **7** (5.00×10^{-2} M) in $CDCl_3$ at 298 K (400 MHz) (red four-pointed star represents residual $CHCl_3$; orange five-pointed star represents CH_2Cl_2, blue inverted triangle represents H_2O).

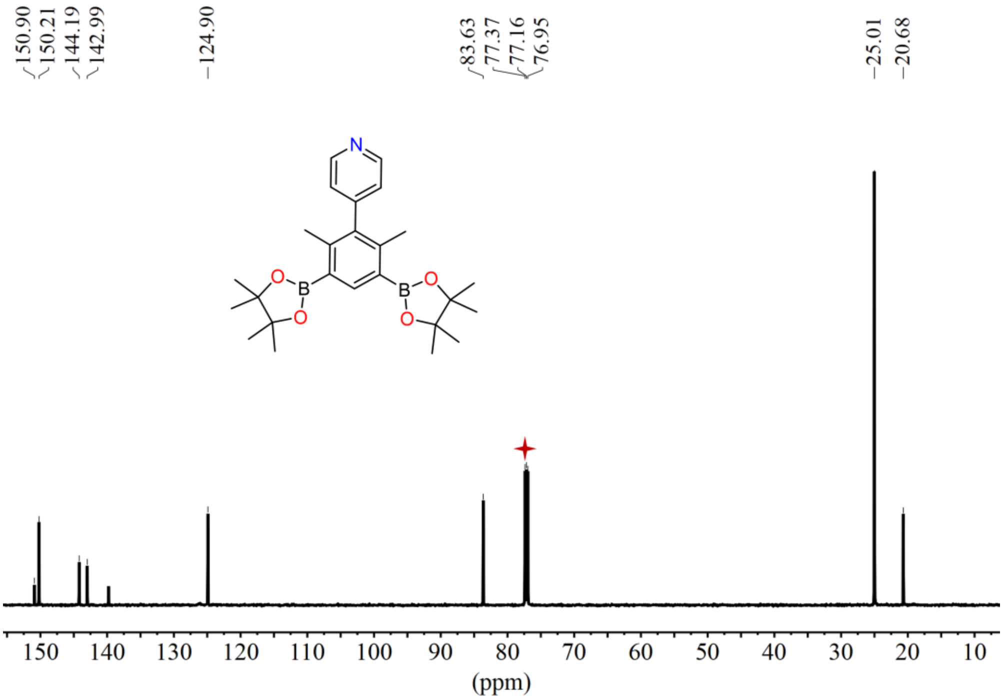

Supplementary Fig. 6 ^{13}C NMR spectrum of **7** (5.00×10^{-2} M) in $CDCl_3$ at 298 K (100 MHz) (red four-pointed star represents residual $CHCl_3$).

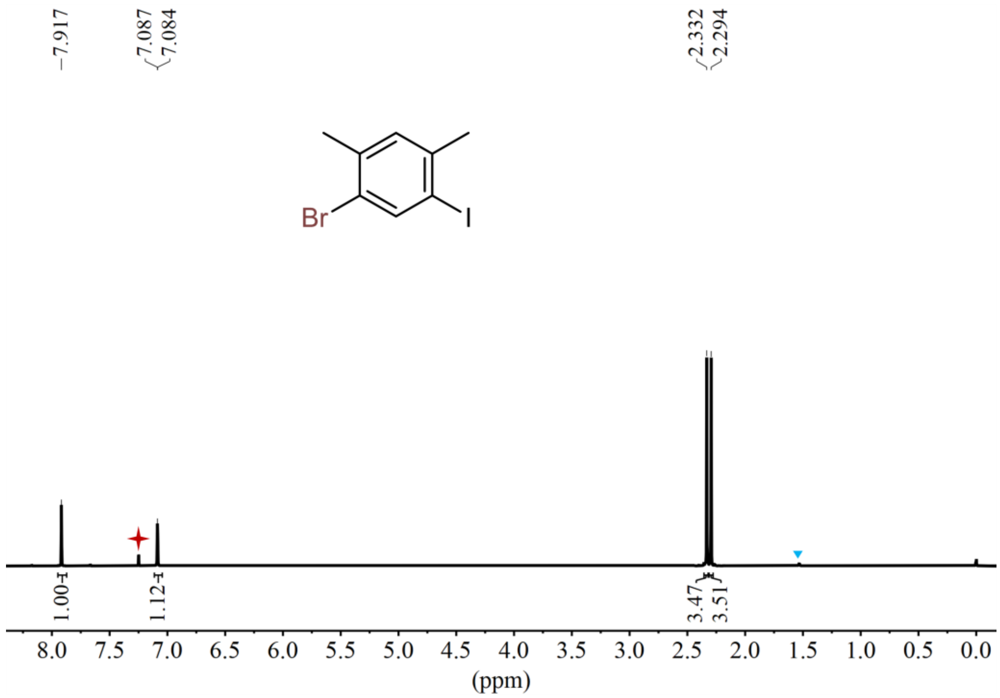

Supplementary Fig. 7 ^{1}H NMR spectrum of **8** (5.00×10^{-2} M) in $CDCl_3$ at 298 K (400 MHz) (red four-pointed star represents residual $CHCl_3$; blue inverted triangle represents H_2O).

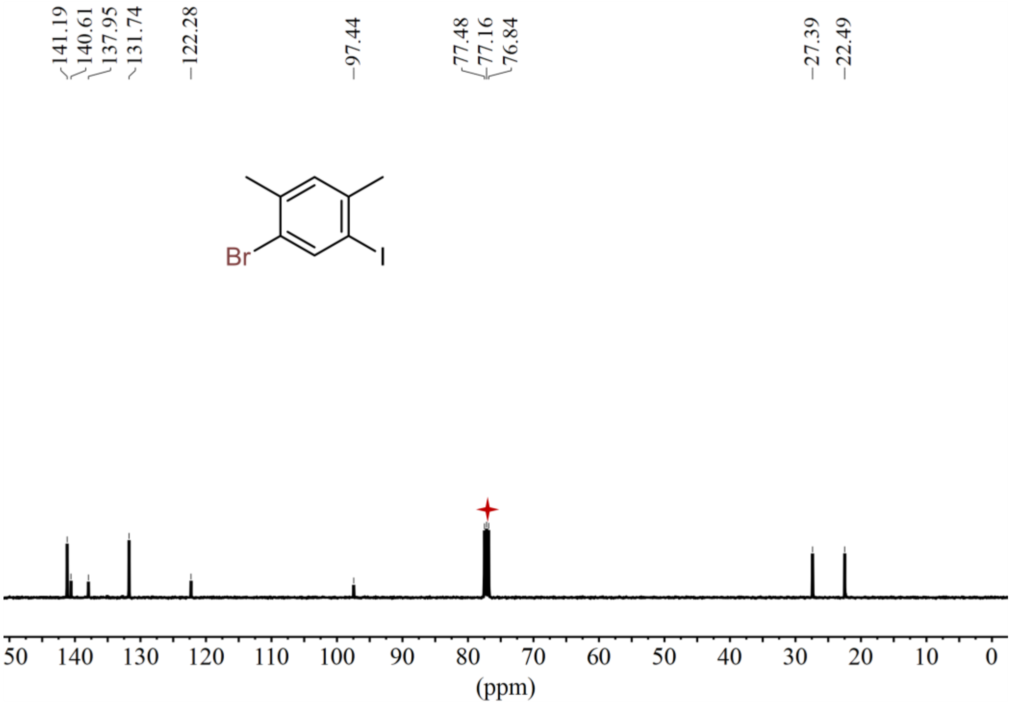

Supplementary Fig. 8 ^{13}C NMR spectrum of **8** (5.00×10^{-2} M) in $CDCl_3$ at 298 K (100 MHz) (red four-pointed star represents residual $CHCl_3$).

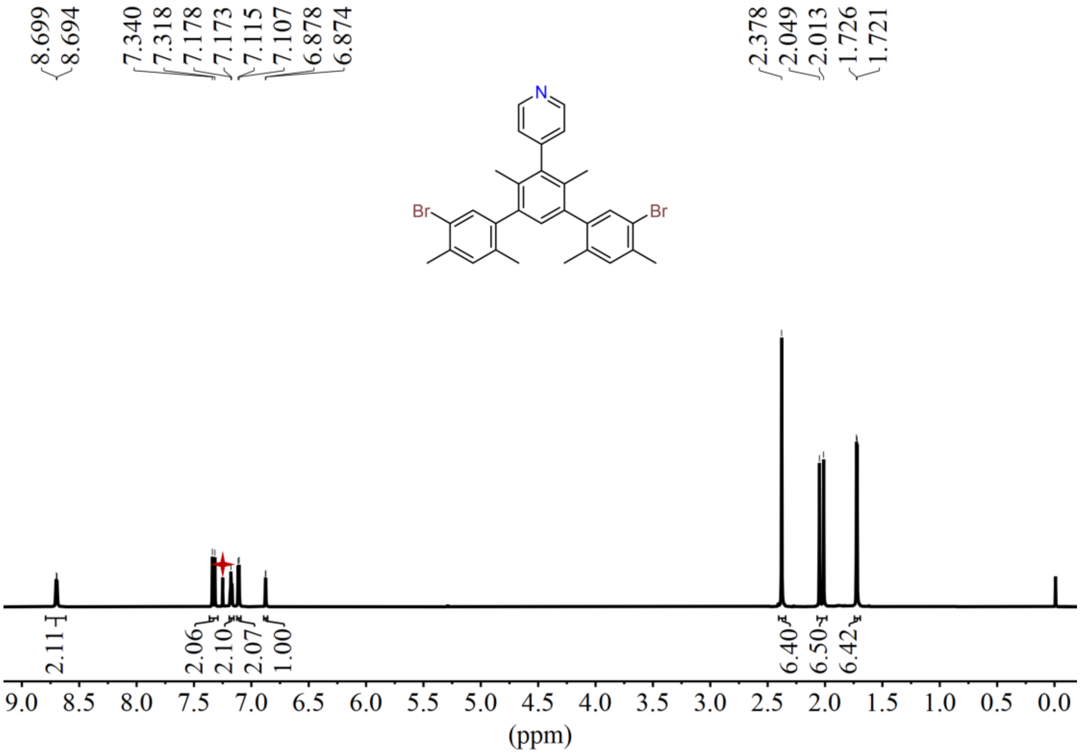

Supplementary Fig. 9 ^{1}H NMR spectrum of **9** (4.00×10^{-2} M) in $CDCl_3$ at 298 K (400 MHz) (red four-pointed star represents residual $CHCl_3$).

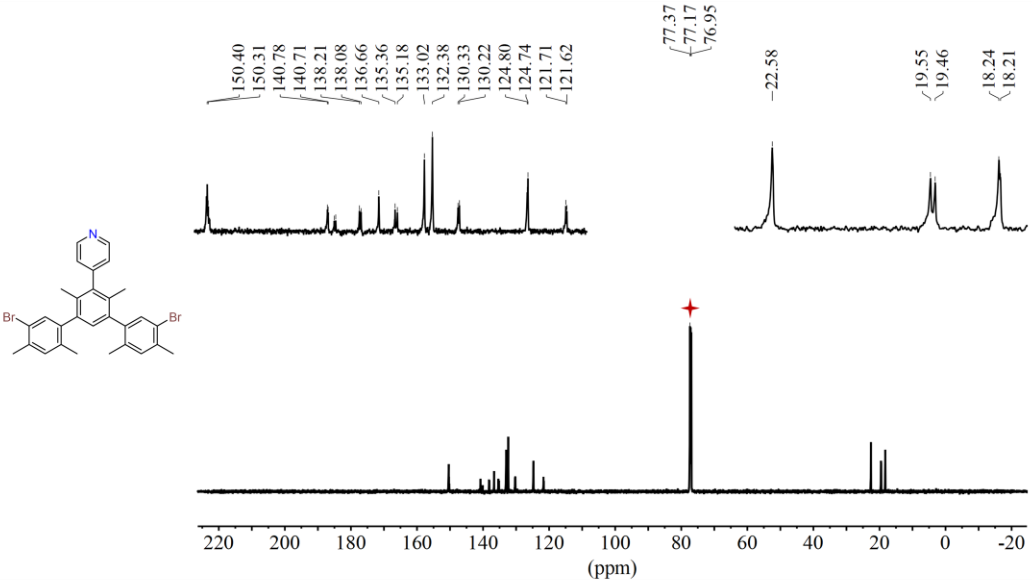

Supplementary Fig. 10 Expanded (up) and full (down) views of ^{13}C NMR spectrum corresponding to **9** (4.00×10^{-2} M) in $CDCl_3$ at 298 K (100 MHz) (red four-pointed star represents residual $CHCl_3$).

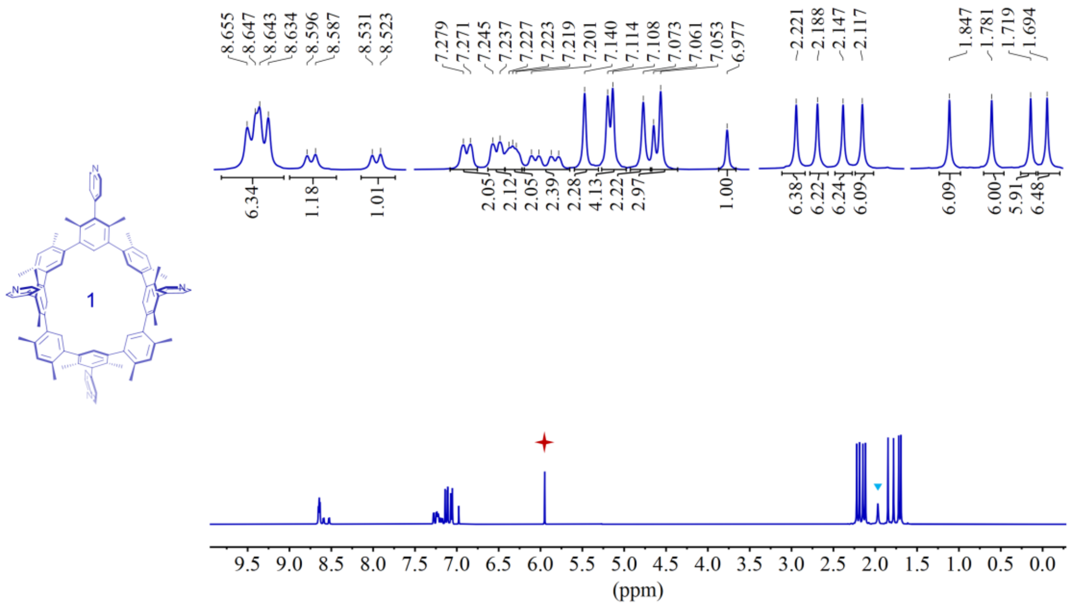

Supplementary Fig. 11 Expanded (up) and full (down) views of ^{1}H NMR spectrum corresponding to **1** (1.00×10^{-2} M) in TCE-d_2 at 298 K (600 MHz) (red four-pointed star represents residual TCE; blue inverted triangle represents cyclohexane).

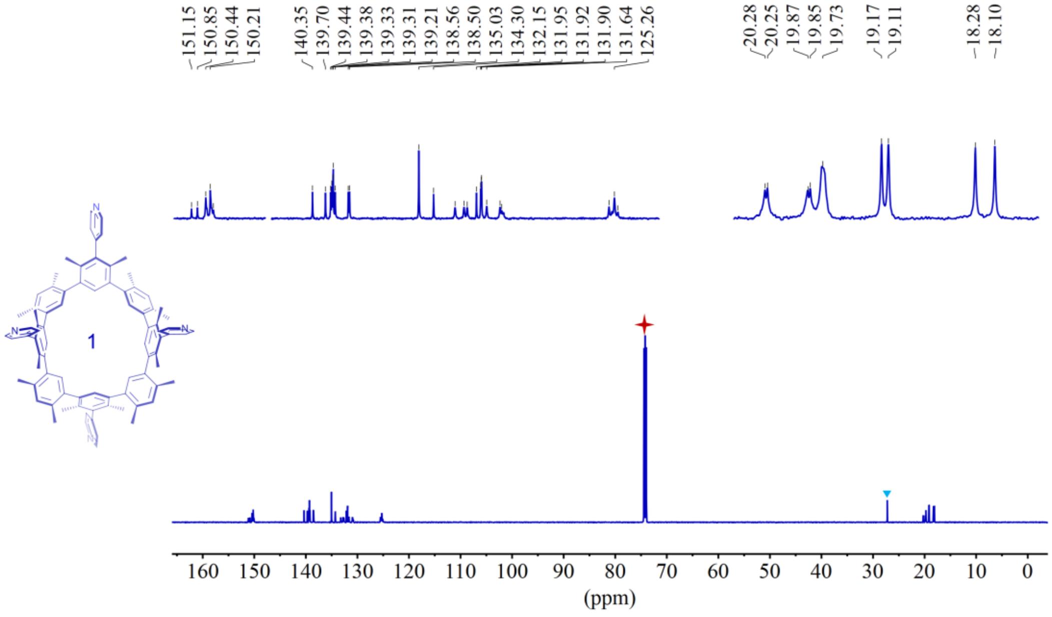

Supplementary Fig. 12 Expanded (up) and full (down) views of ^{13}C NMR spectrum corresponding to **1** (3.00×10^{-2} M) in TCE-d_2 at 298 K (150 MHz) (red four-pointed star represents residual TCE; blue inverted triangle represents cyclohexane).

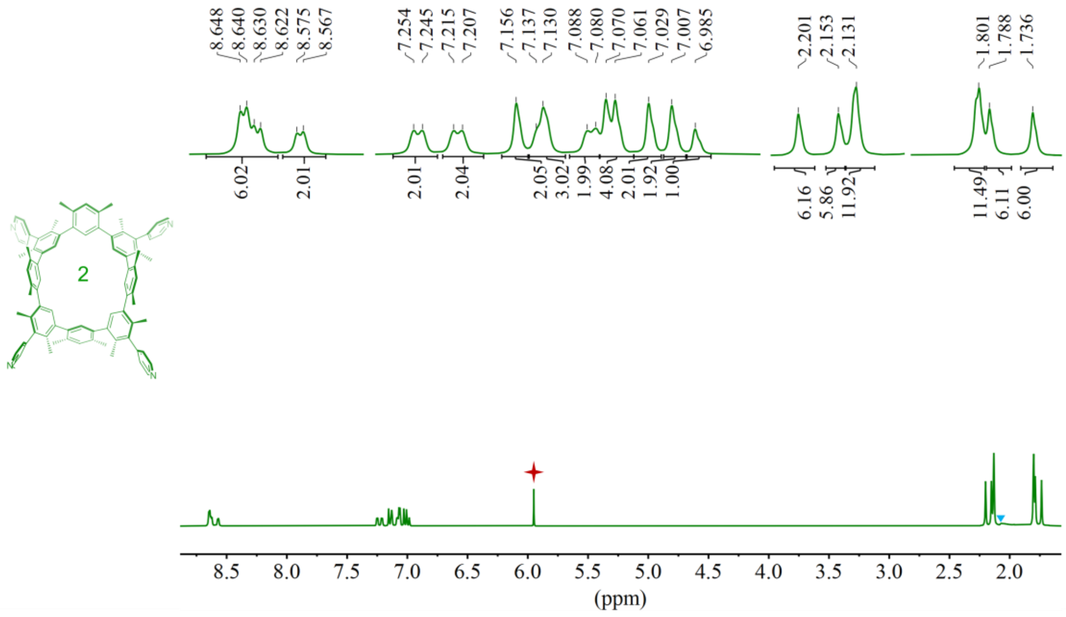

Supplementary Fig. 13 Expanded (up) and full (down) views of ^{1}H NMR spectrum corresponding to **2** (1.00×10^{-2} M) in TCE-d_2 at 298 K (600 MHz) (red four-pointed star represents residual TCE; blue inverted triangle represents cyclohexane).

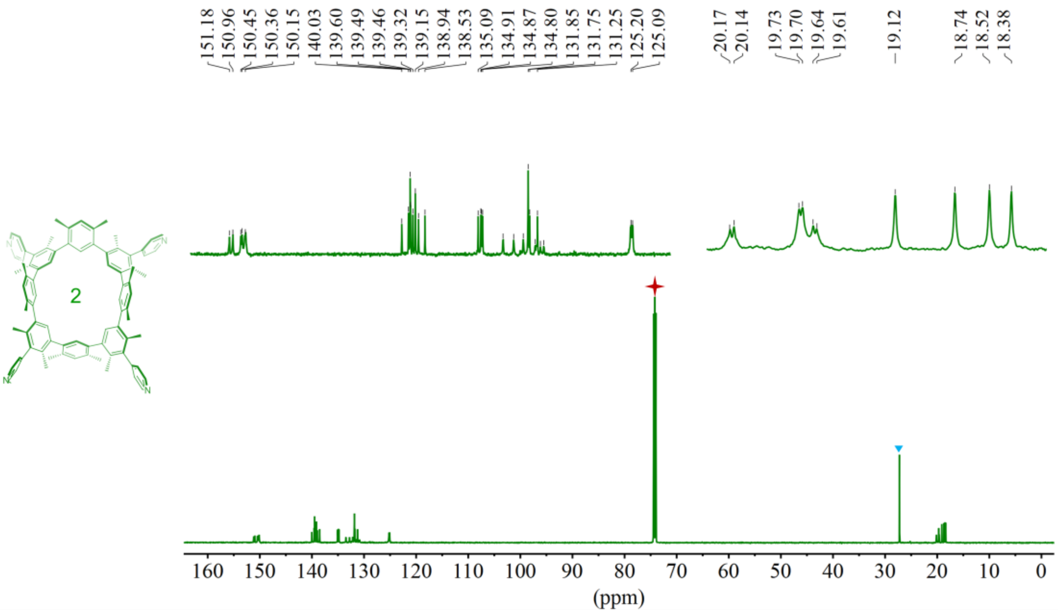

Supplementary Fig. 14 Expanded (up) and full (down) views of ^{13}C NMR spectrum corresponding to **2** (3.00×10^{-2} M) in TCE-d_2 at 298 K (150 MHz) (red four-pointed star represents residual TCE; blue inverted triangle represents cyclohexane).

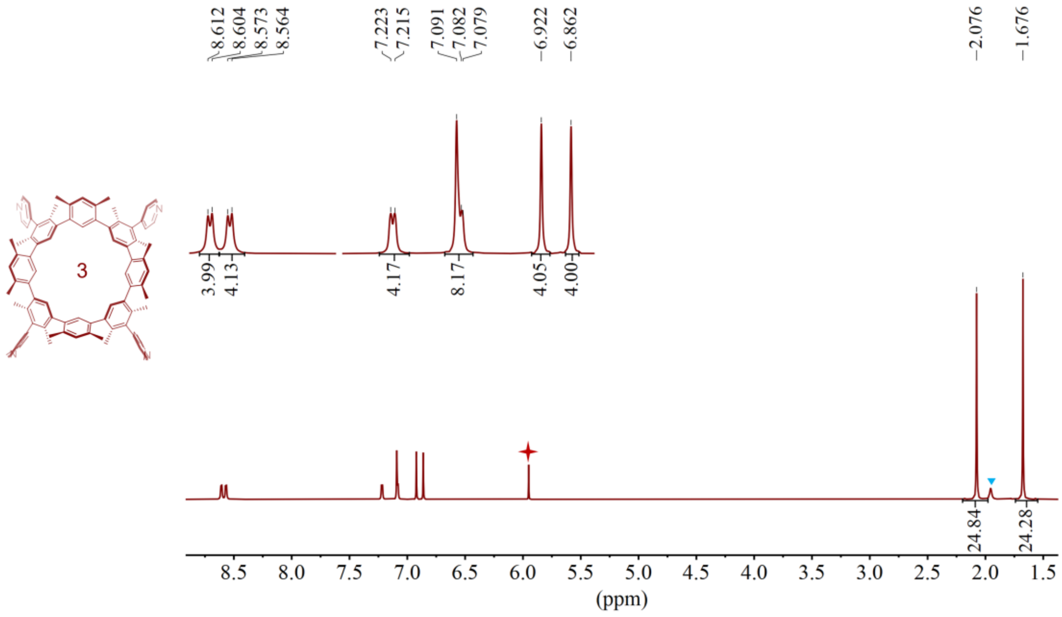

Supplementary Fig. 15 Expanded (inserted) and full views of ^{1}H NMR spectrum corresponding to **3** (1.00 × 10^{-2} M) in TCE-d_2 at 298 K (600 MHz) (red four-pointed star represents residual TCE; blue inverted triangle represents cyclohexane).

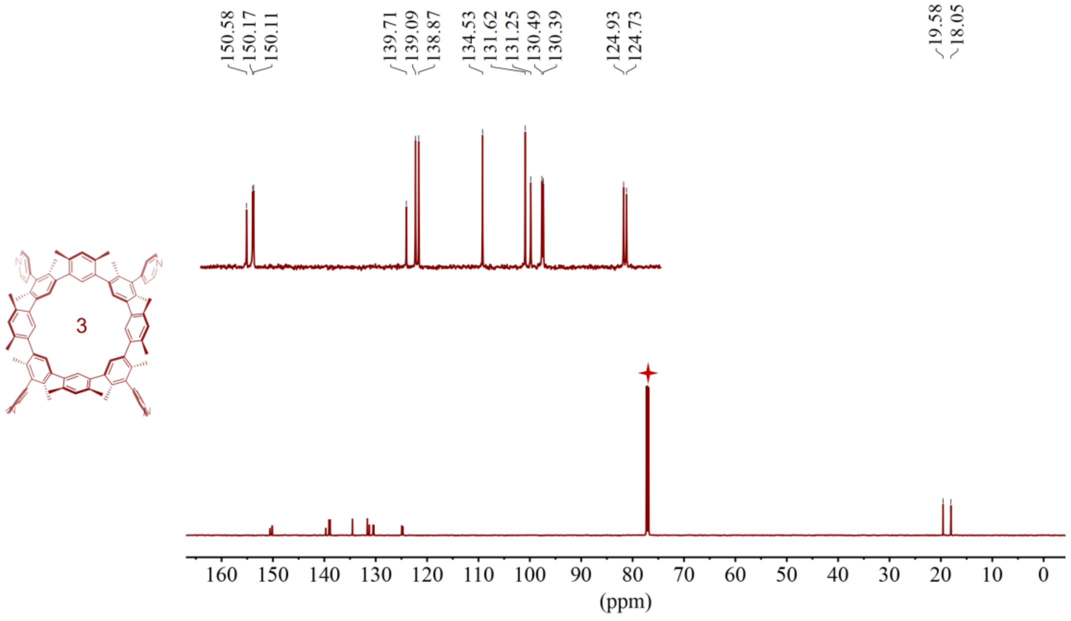

Supplementary Fig. 16 Expanded (inserted) and full views of ^{13}C NMR spectrum corresponding to **3** (3.00 × 10^{-2}) in TCE-d_2 at 298 K (150 MHz) (red four-pointed star represents residual TCE).

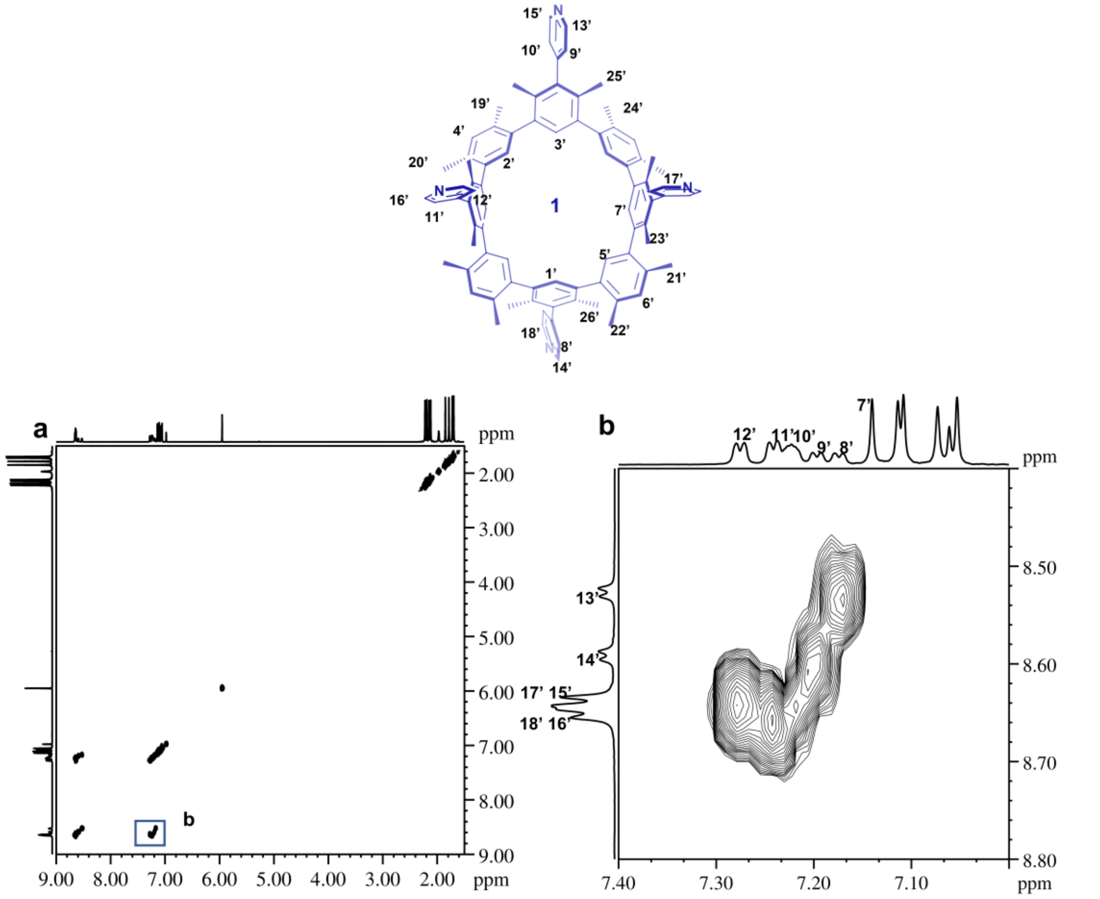

Supplementary Fig. 17 The top blue illustration shows the molecular structure formula of **1**. Full (a) and expanded views (b) of the COSY spectrum corresponding to **1** (3.0×10^{-2} M) in TCE-d_2 at 298 K (600 MHz).

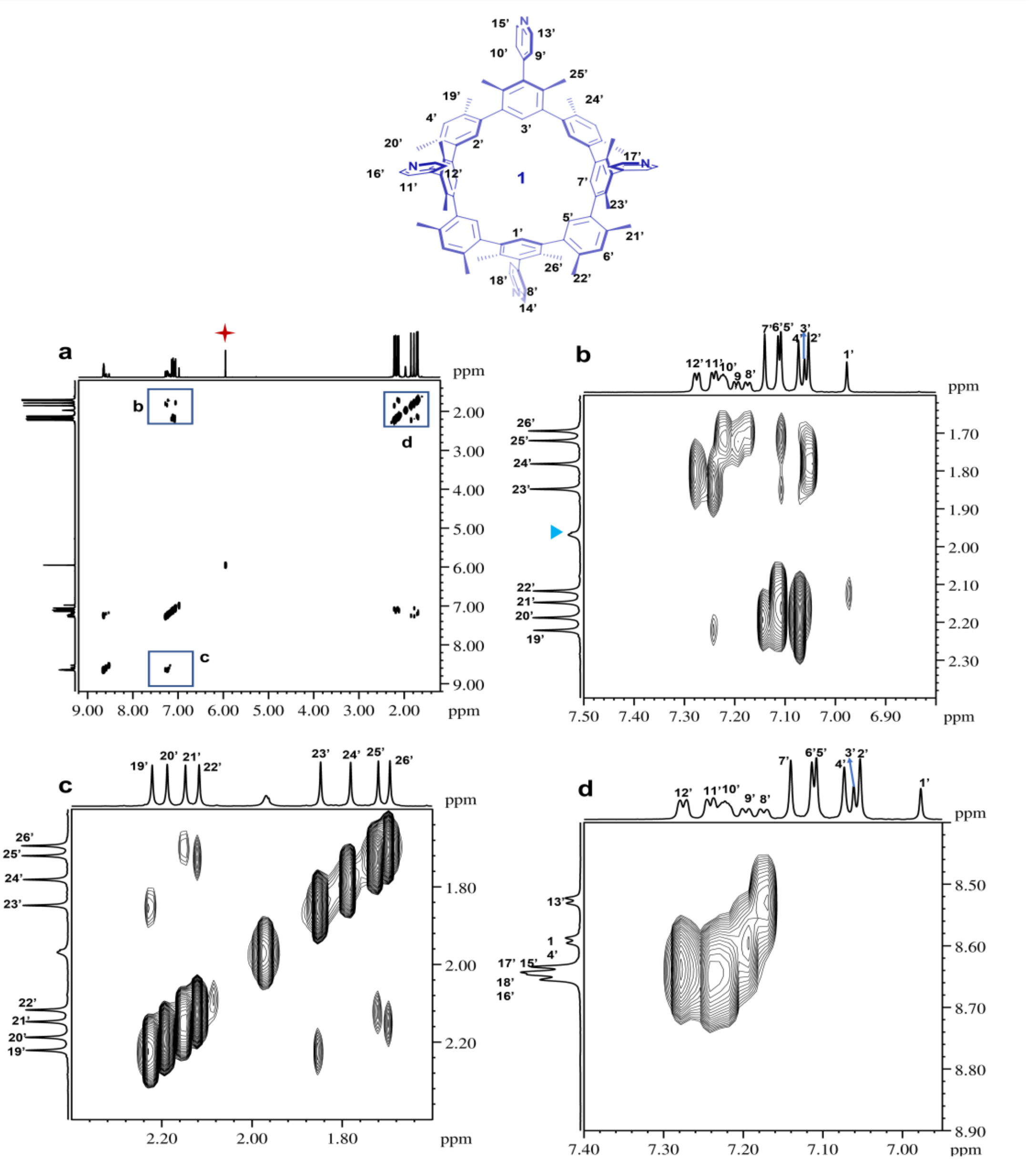

Supplementary Fig. 18 The top blue illustration shows the molecular structure formula of **1**. Full (a) and expanded views (b, c, d) of the NOESY spectrum corresponding to **1** (3.00×10^{-2} M) in TCE at 298 K (600 MHz) (red four-pointed star represents residual TCE; blue inverted triangle represents cyclohexane).

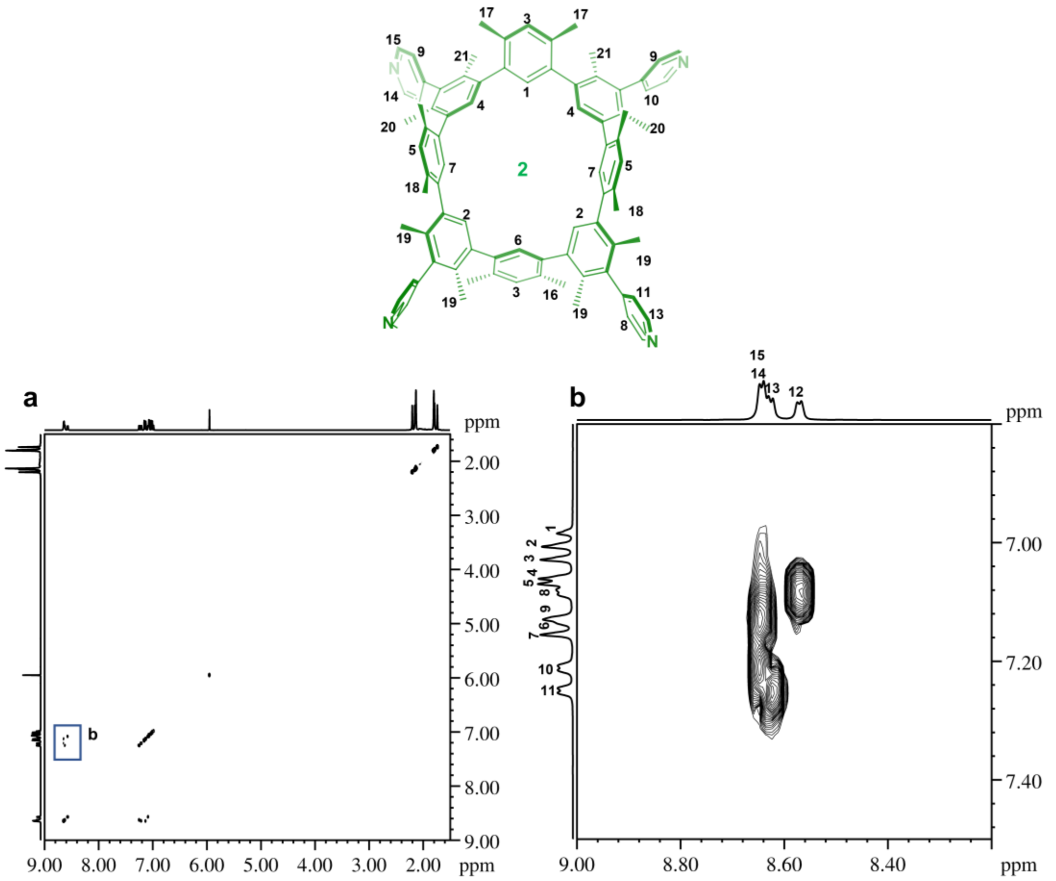

Supplementary Fig. 19 The top green illustration shows the molecular structure formula of **2**. Full (a) and expanded views (b) of the COSY spectrum corresponding to **2** (3.0×10^{-2} M) in TCE-d_2 at 298 K (600 MHz).

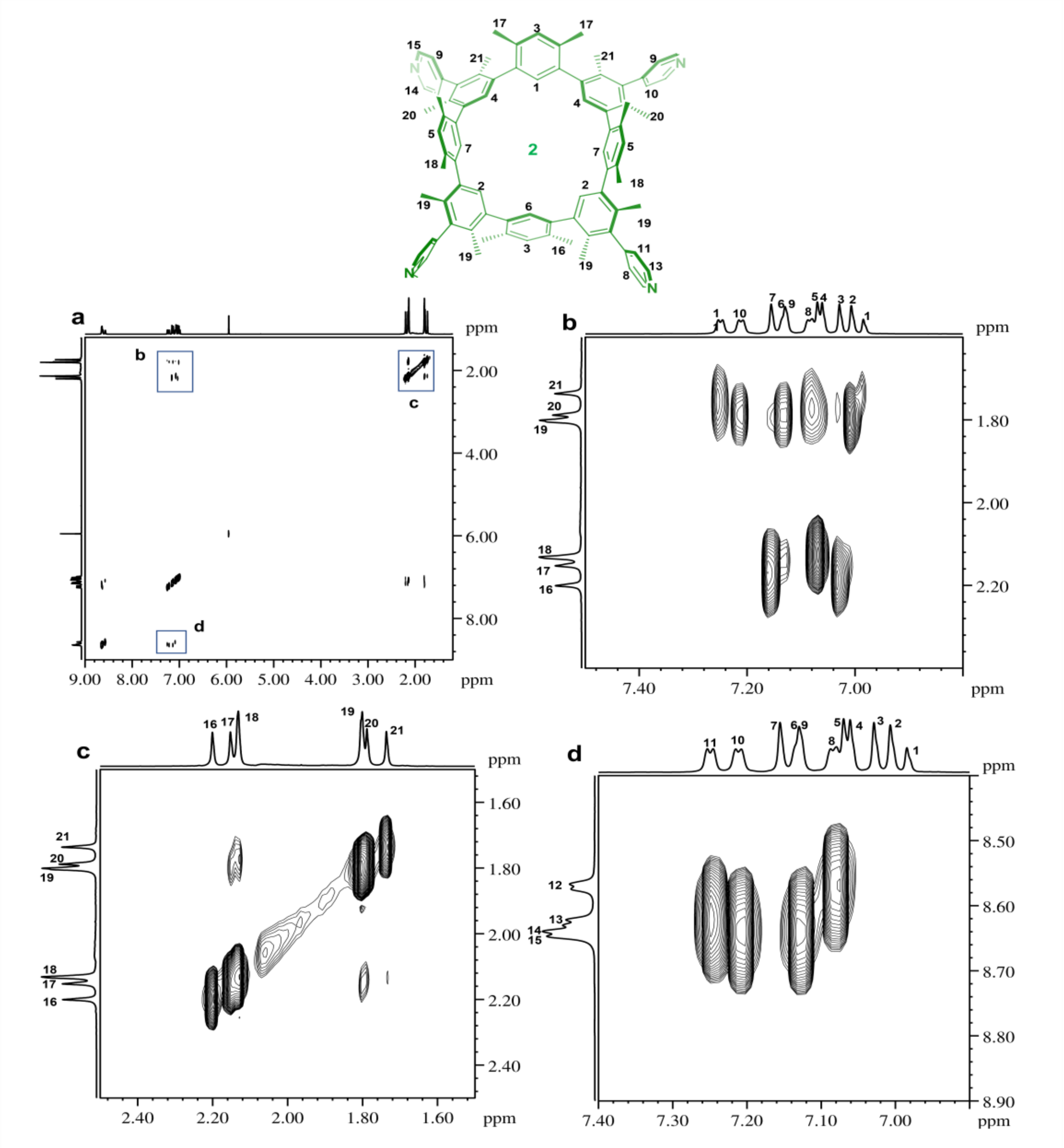

Supplementary Fig. 20 The top green illustration shows the molecular structure formula of **2**. Full (a) and expanded views (b, c, d) of the NOESY spectrum corresponding to **2** (3.00×10^{-2} M) in TCE-d_2 at 298 K (600 MHz).

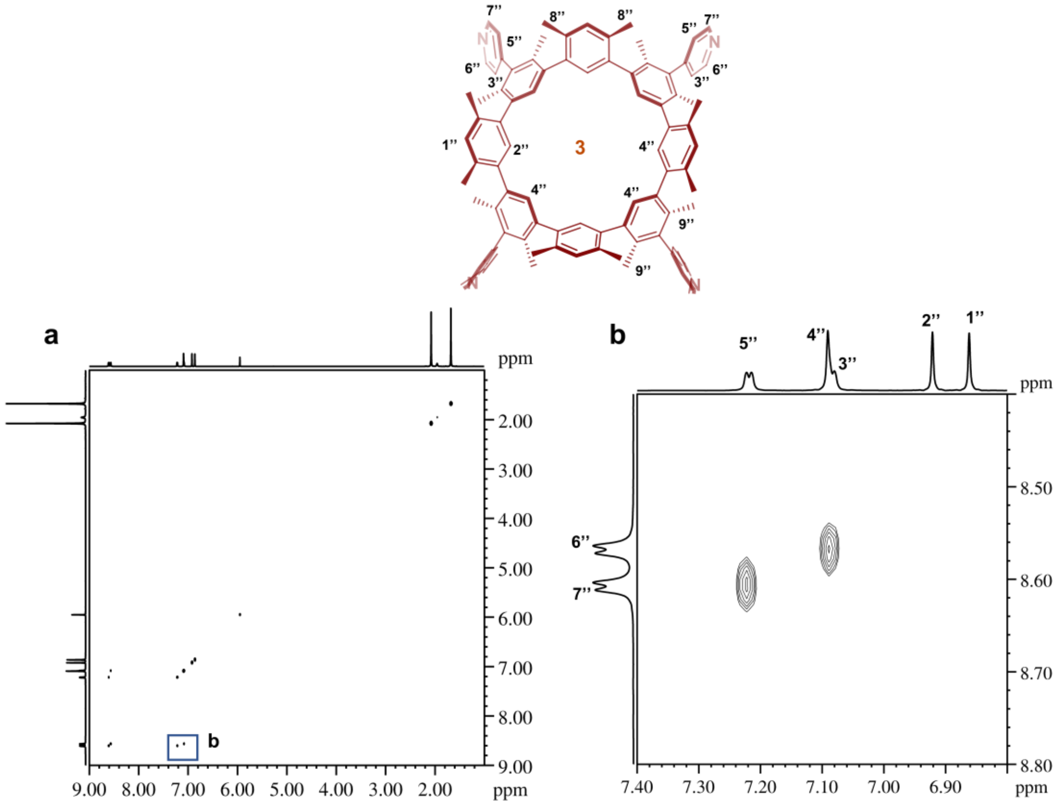

Supplementary Fig. 21 The top red illustration shows the molecular structure formula of **3**. Full (a) and expanded views (b) of the COSY spectrum corresponding to **3** (3.0×10^{-2} M) in TCE-d_2 at 298 K (600 MHz).

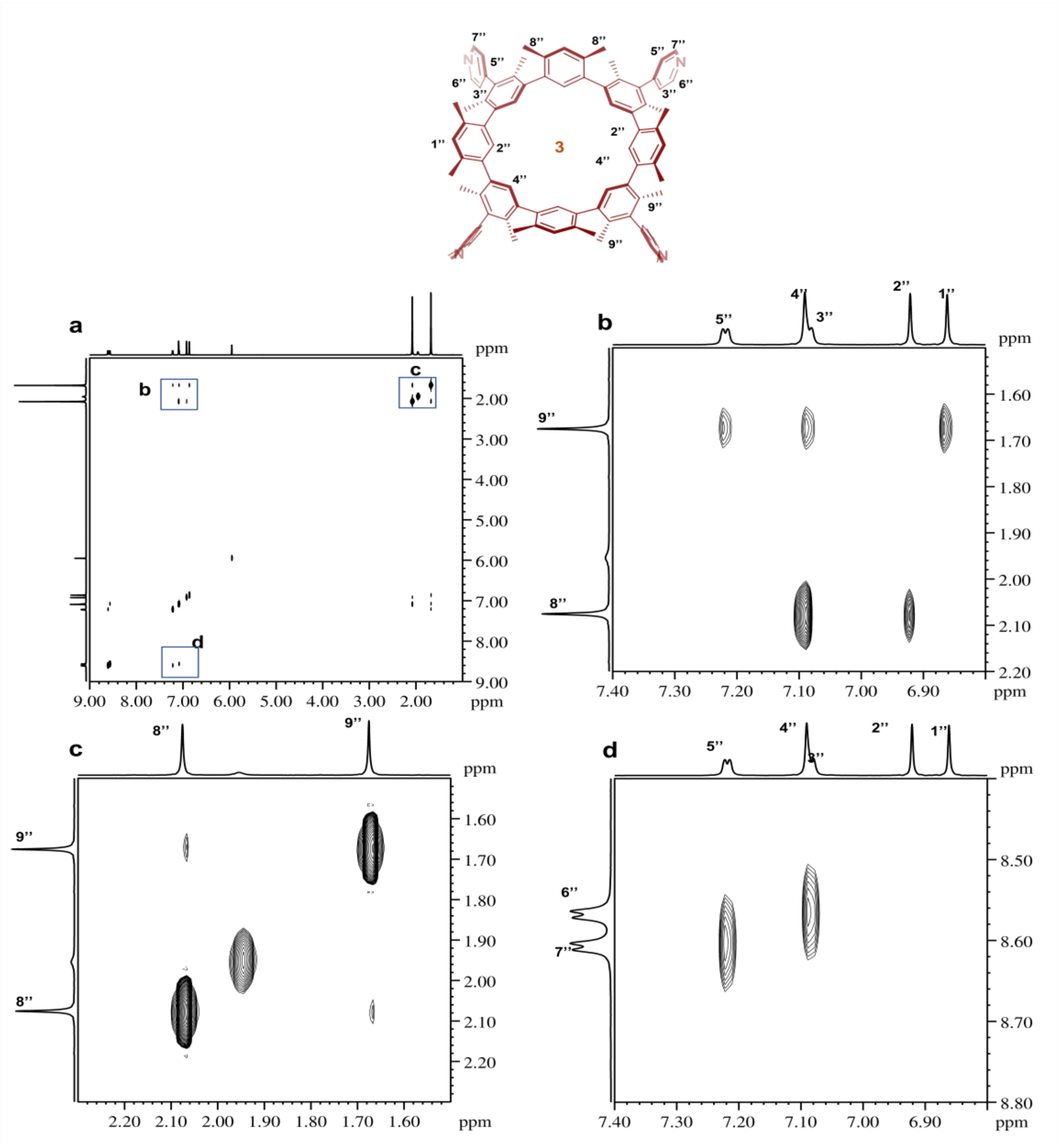

Supplementary Fig. 22 The top red illustration shows the molecular structure formula of **3**. Full (a) and expanded views (b, c, d) of the NOESY spectrum corresponding to **3** (3.00×10^{-2} M) in TCE-d_2 at 298 K (600 MHz).

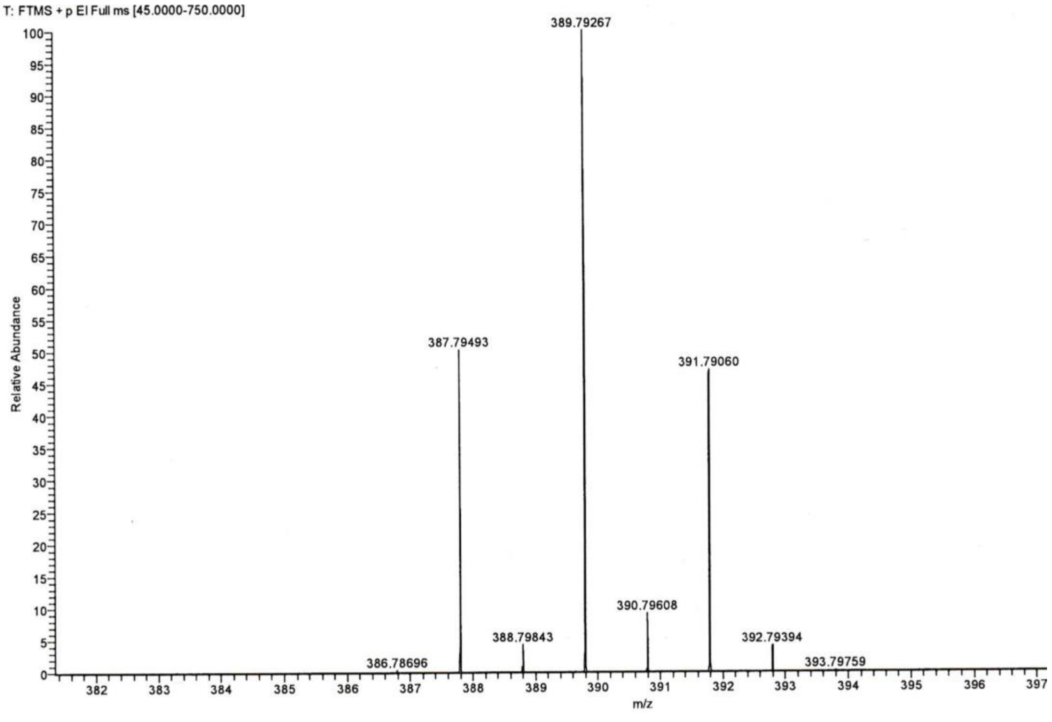

Supplementary Fig. 23 Positive EI HRMS spectrum of **5**, EI HRMS (*m*/*z*): [M-H]$^{+•}$ calcd. for $C_8H_7Br_2I$, 389.8012; found, 389.7927.

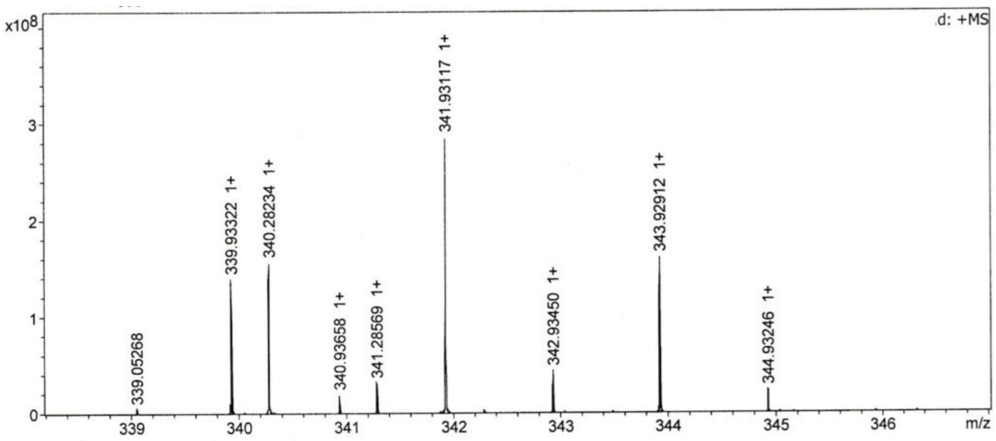

Supplementary Fig. 24 Positive ESI HRMS spectrum of **6**, ESI HRMS (*m*/*z*): [M+H]$^{+•}$ calcd. for $C_{13}H_{11}Br_2N$, 341.9311; found, 341.9312.

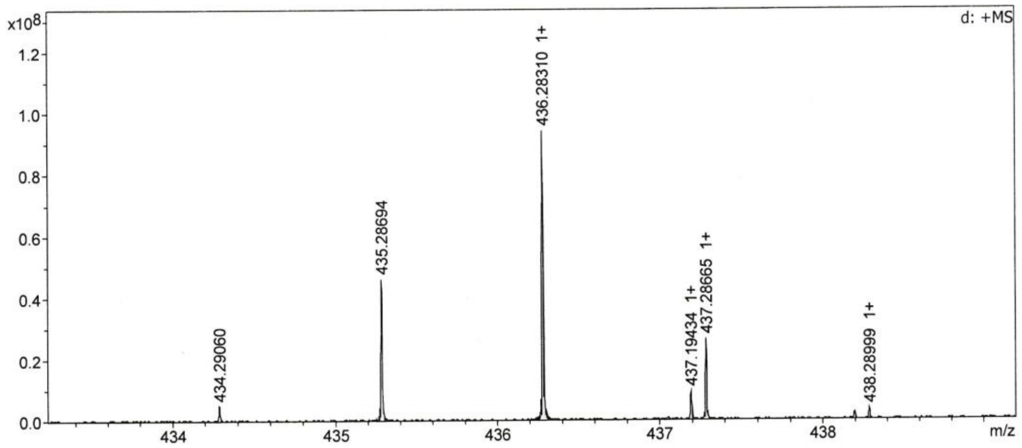

Supplementary Fig. 25 Positive ESI HRMS spectrum of **7,** ESI HRMS (*m*/*z*): $[M]^{+\bullet}$ calcd. for $C_{25}H_{35}B_2NO_4$, 436.2834; found, 436.2831.

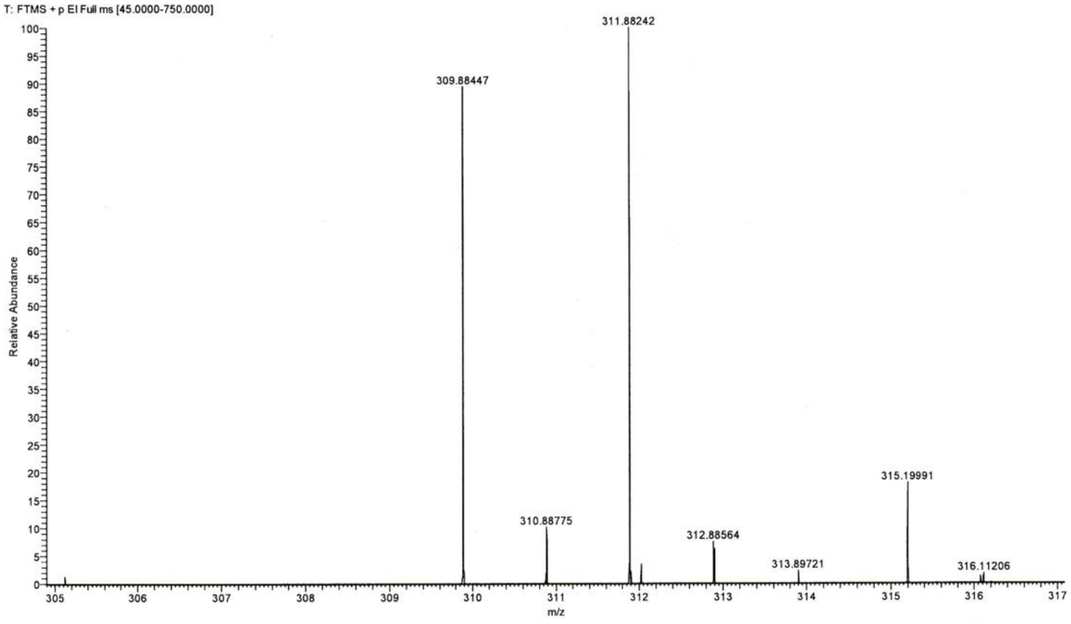

Supplementary Fig. 26 Positive EI HRMS spectrum of **8**, ESI HRMS (*m*/*z*): $[M\text{-}H]^{+\bullet}$ calcd. for C_8H_8BrI, 309.8927; found, 309.8845.

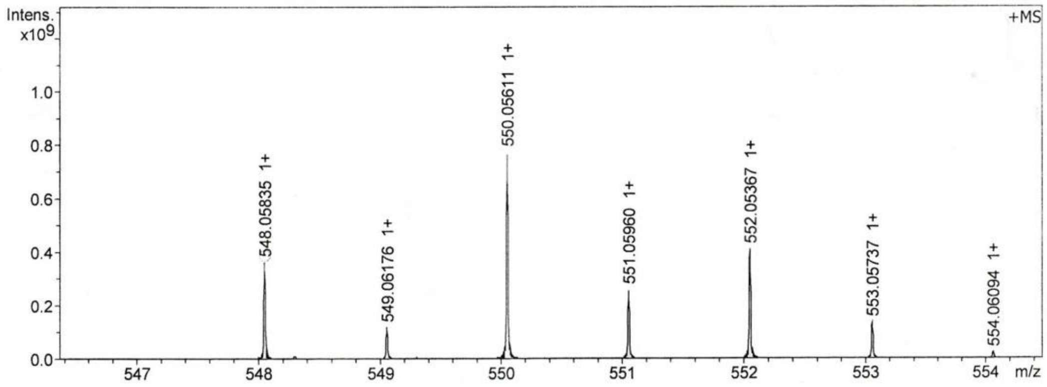

Supplementary Fig. 27 Positive ESI HRMS spectrum of **9**, MALDI-TOF HRMS (*m/z*): $[M+H]^{+\bullet}$ calcd. for $C_{29}H_{27}Br_2N$, 550.0565; found, 550.0561.

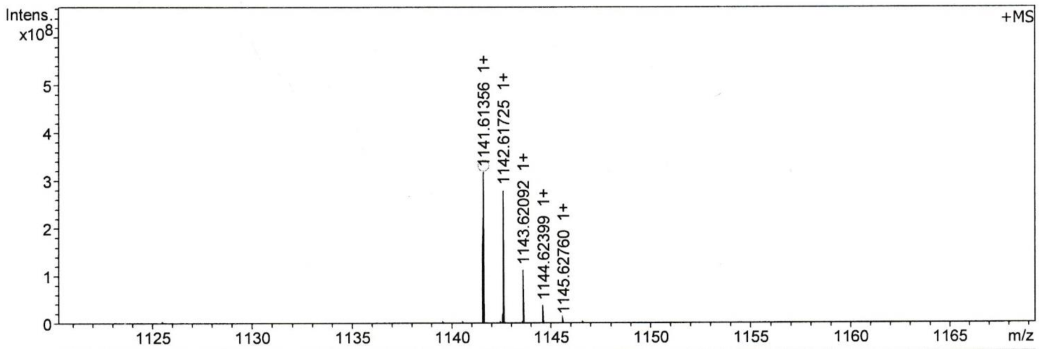

Supplementary Fig. 28 Positive MALDI-TOF HRMS spectrum of **1**, MALDI-TOF HRMS (*m/z*): $[M]^{+\bullet}$ calcd. for $C_{84}H_{76}N_4$, 1141.6143; found, 1141.6136.

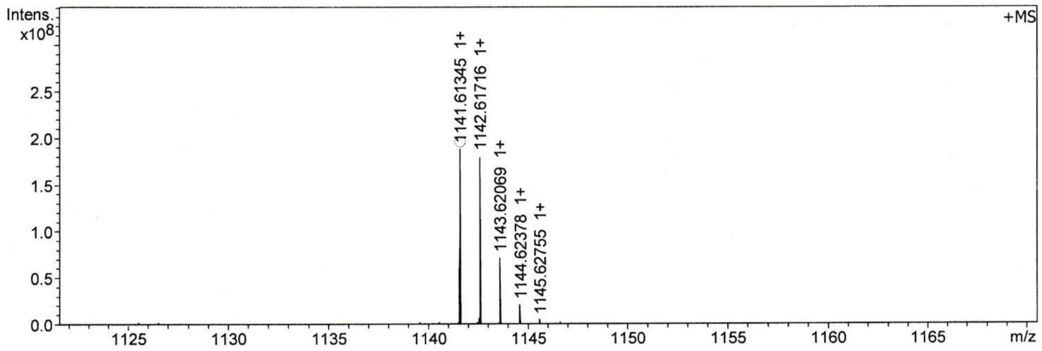

Supplementary Fig. 29 Positive MALDI-TOF HRMS spectrum of **2**, MALDI-TOF HRMS (*m/z*): $[M]^{+\bullet}$ calcd. for $C_{84}H_{76}N_4$, 1141.6143; found, 1141.6134.

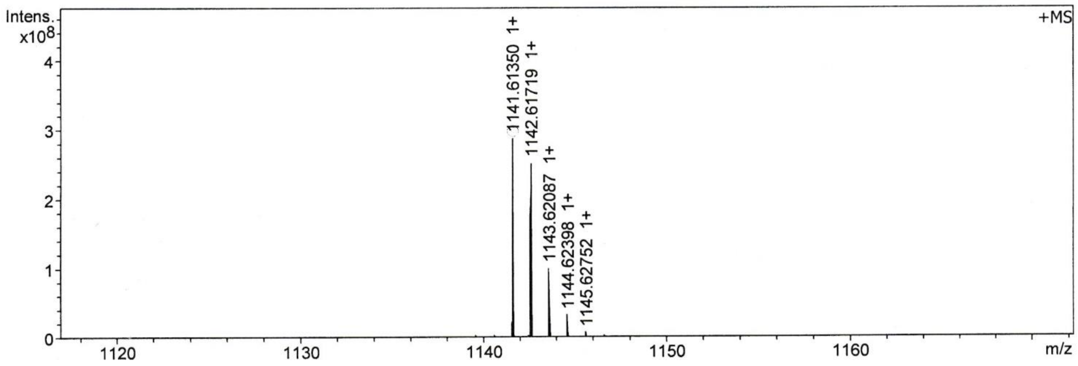

Supplementary Fig. 30 Positive MALDI-TOF HRMS spectrum of **3**, MALDI-TOF HRMS (*m/z*): $[M]^{+\bullet}$ calcd. for $C_{84}H_{76}N_4$, 1141.6143; found, 1141.6135.

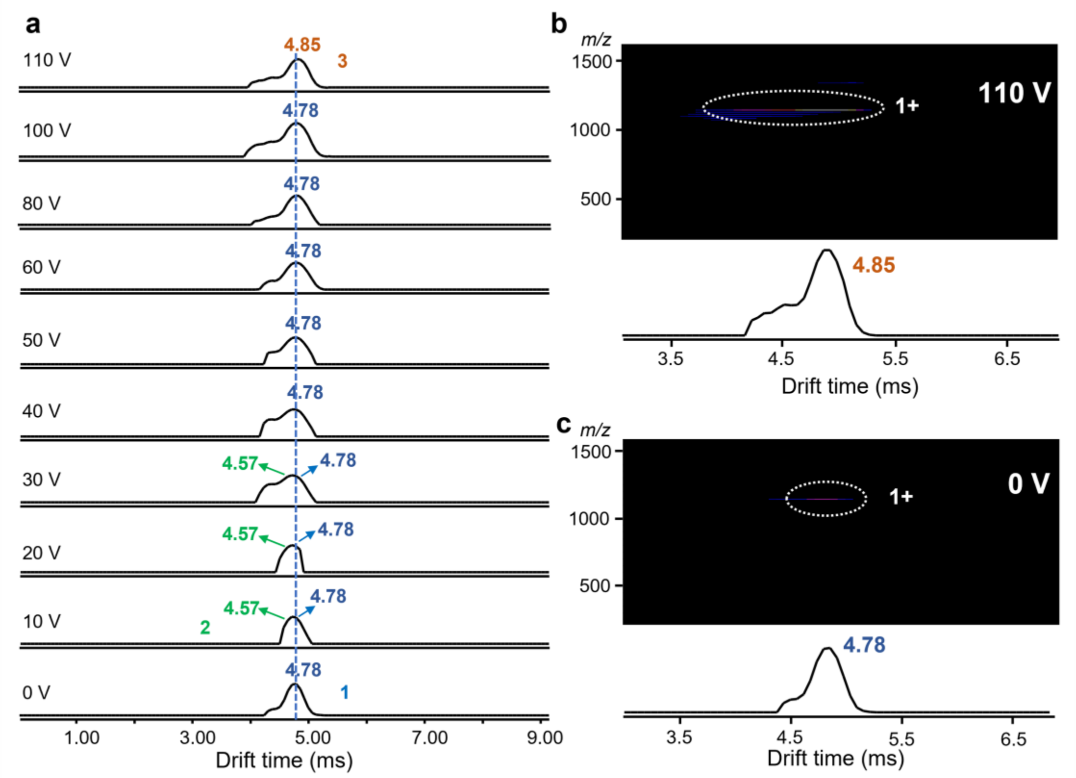

Supplementary Fig. 31 Arrival time distributions of the species formed from **1** at 0-110 V collision voltage in the mass-selected CID/IMS experiments (a), TWIM-MS spectra of **1** at 110 V (b) and 0 V (c) collision voltage.

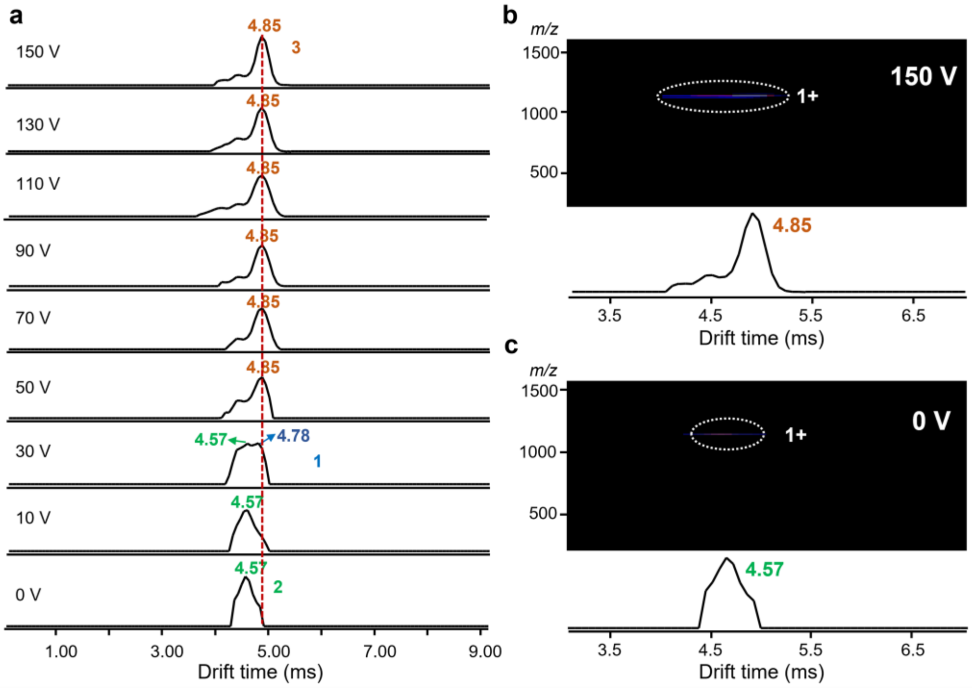

Supplementary Fig. 32 Arrival time distributions of the species formed from **2** at 0-150 V collision voltage in the mass-selected CID/IMS experiments (a), TWIM-MS spectra of **2** at 150 V (b) and 0 V (c) collision voltage.

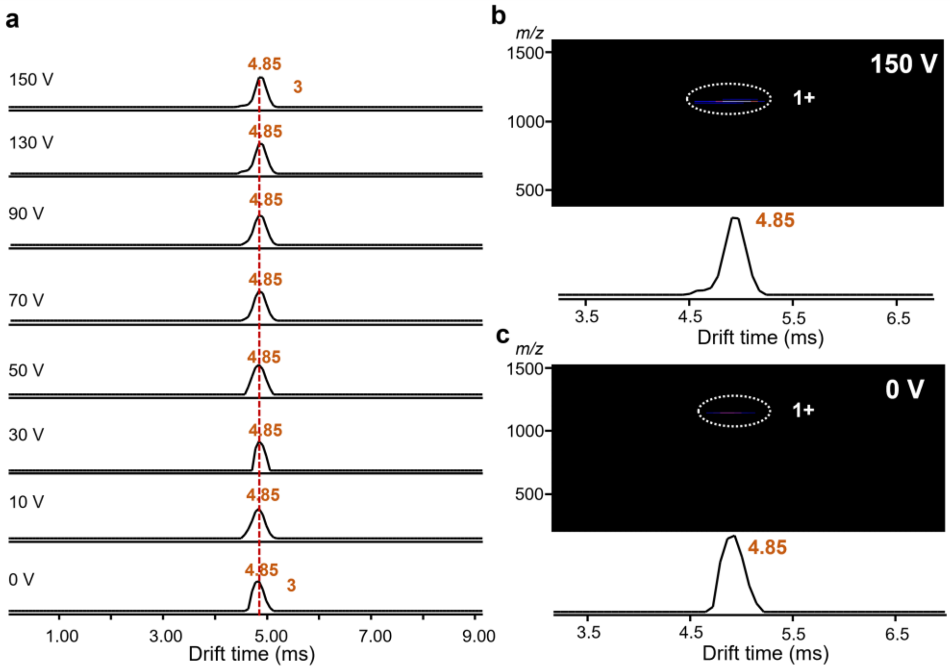

Supplementary Fig. 33 Arrival time distributions of the species formed from **3** at 0-150 V collision voltage in the mass-selected CID/IMS experiments (a), TWIM-MS

spectra of **3** at 150 V (b) and 0 V (c) collision voltage.

Supplementary Note 3: Theoretical formation energy calculation of cyclization intermediates (**1-im, 2-im**, and **3-im**).

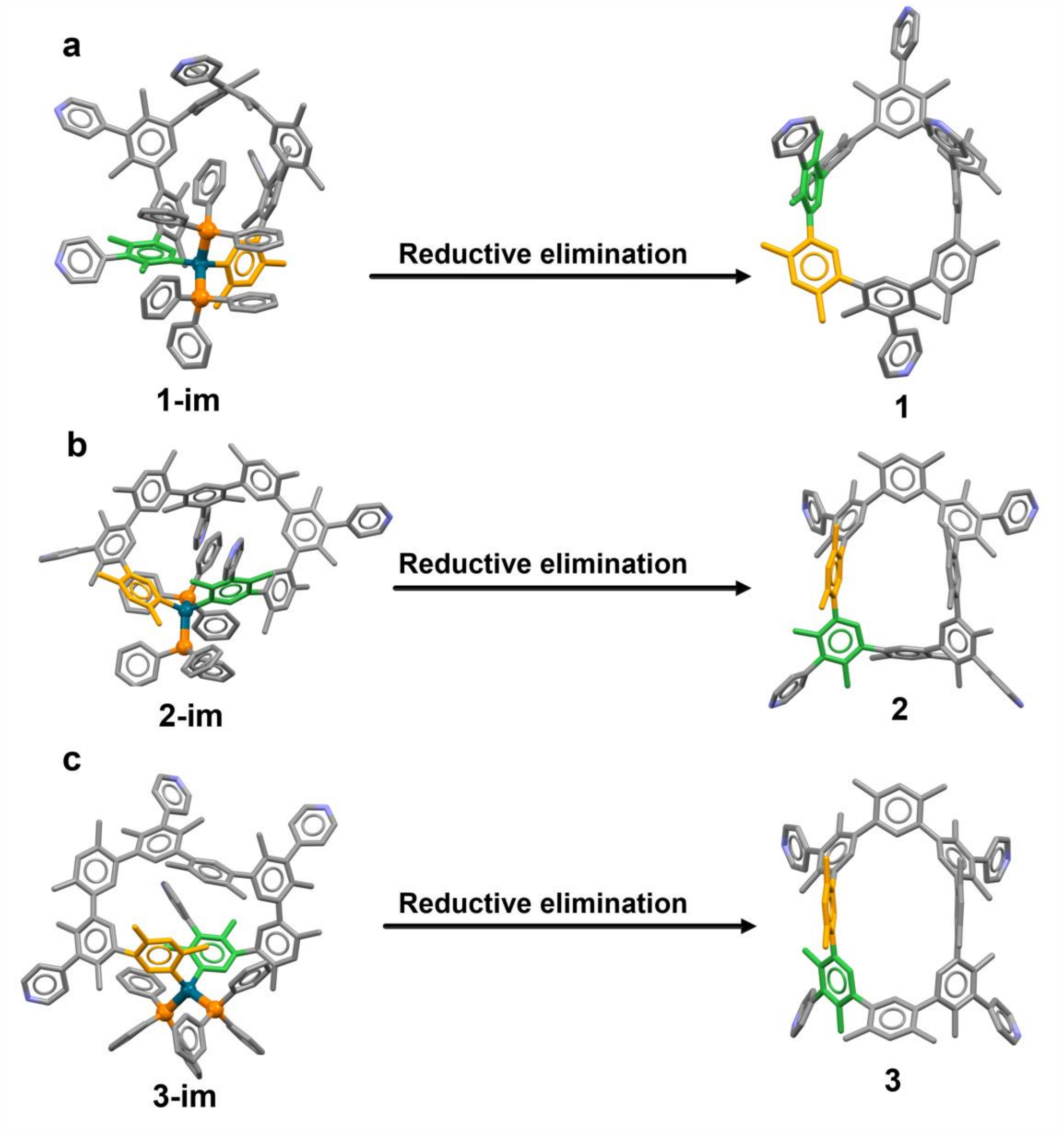

Supplementary Fig. 34 Reductive elimination step from **1-im** to **1** (a), from **2-im** to **2** (b), or from **3-im** to **3** (c). The lowest formation heat values of **1-im, 2-im**, and **3-im** in vacuum were calculated via molecular mechanics (MM+) using the force filed in the HyperChem 8.0 program[5], or semiempirical methods (PM7) in MOPAC program[6]. All the hydrogen atoms were omitted for clarity. Carbon: silvery grey, yellow or green, Nitrogen: bluish violet, Palladium cyanine, Phosphorus orange.

Supplementary Table 1. The formation heat (***E***) and the relative formation heat (Δ***E***) values of **1-im**, **2-im**, and **3-im** as calculated in vacuum.

Method / Species	MM+		PM7	
	E (kJ mol^{-1})	Δ***E*** = ***E***$_{(\mathbf{n}\text{-im})}$- ***E***$_{(\mathbf{1}\text{-im})}$ (kJ mol^{-1})	***E*** (kJ mol^{-1})	Δ***E*** = ***E***$_{(\mathbf{n}\text{-im})}$- ***E***$_{(\mathbf{1}\text{-im})}$ (kJ mol^{-1})
**1**-im	538	0	1245	0
**2**-im	531	-7.0	1320	75
**3**-im	573	35	1408	163

Supplementary Note 4: X-ray crystallography of single crystal structures **1**•2.25CH_2Cl_2, **2**•CH_2Cl_2•0.75H_2O and **3**•$CH_3COOC_2H_5$•2CH_3CN•3.5H_2O.

Supplementary Table 2. X-ray crystallographic data comparison of **1**•2.25CH_2Cl_2, **2**•CH_2Cl_2•0.75H_2O and **3**•$CH_3COOC_2H_5$•2CH_3CN•3.5H_2O.

	**1**•2.25•CH_2Cl_2	**2**•CH_2Cl_2•0.75H_2O	**3**•$CH_3COOC_2H_5$•2CH_3CN•3.5H_2O
CCDC No.	2280091	2280092	2280089
Empirical formula	$C_{86.25} H_{80.50} Cl_{4.50} N_4$	$C_{85} H_{79.50} Cl_2 N_4 O_{0.75}$	$C_{92} H_{97} N_6 O_{5.50}$
Formula weight	1332.57	1239.92	1374.75
Temperature/K	169.99(13)	169.99(16)	100.00(10)
Crystal system	orthorhombic	monoclinic	triclinic
Space group	P n m a	P 21/c	P-1
a [Å]	13.2724(8)	16.7411(9)	15.4676(3)
b [Å]	27.336(3)	16.0298(8)	16.1929(3)
c [Å]	20.0597(15)	26.5504(12)	16.3730(3)
α [deg]	90.00	90.00	90.832(2)
β [deg]	90.00	97.788(4)	100.813(2)
γ [deg]	90.00	90.00	91.175(2)
Volume/ [$Å^3$]	7278.1(10)	7059.3(6)	4026.50(13)
Z	4	4	2
ρ_{calc} (g/cm^3)	1.216	1.167	1.134
Absorption coefficient/mm^{-1}	2.010	1.195	0.549
F(000)	2810	2630	1470
crystal size (mm^3)	0.050 × 0.020 × 0.020	0.180 × 0.150 × 0.100	0.300 × 0.200 × 0.100
Radiation	Cu-Kα (λ = 1.54184)	Cu-Kα (λ = 1.54184)	Cu-Kα (λ = 1.54184)
2Θ range for data collection/°	5.464 to 124.984	5.328 to 124.994	7.216 to 124.998
Index ranges	$-10 \le h \le 15$, $-31 \le k \le 30$, $-22 \le l \le 23$	$-19 \le h \le 19$, $-18 \le k \le 18$, $-30 \le l \le 28$	$-16 \le h \le 17$, $-18 \le k \le 18$, $-18 \le l \le 16$
Reflections collected	22930	40703	55599
Independent reflections	5925 [R_{int} = 0.0884, R_{sigma} = 0.0785]	11267 [R_{int} = 0.0716, R_{sigma} = 0.0593]	12820 [R_{int} = 0.0405, R_{sigma} = 0.0292]
Data/restraints/parameters	5925/210/471	11267/48/865	12820/339/1055
Goodness-of-fit on F^2	1.012	1.001	1.020
Final R indexes [I>=2σ (I)]	R_1 = 0.0986, wR_2 = 0.2060	R_1 = 0.0866, wR_2 = 0.2363	R_1 = 0.1079, wR_2 = 0.2366
Final R indexes [all data]	R_1 = 0.1720, wR_2 = 0.2440	R_1 = 0.1429, wR_2 = 0.2867	R_1 =0.0775, wR_2 = 0.2400
Largest diff. peak/hole/e $Å^{-3}$	0.65/-0.60	1.48/-0.45	1.14/-0.78

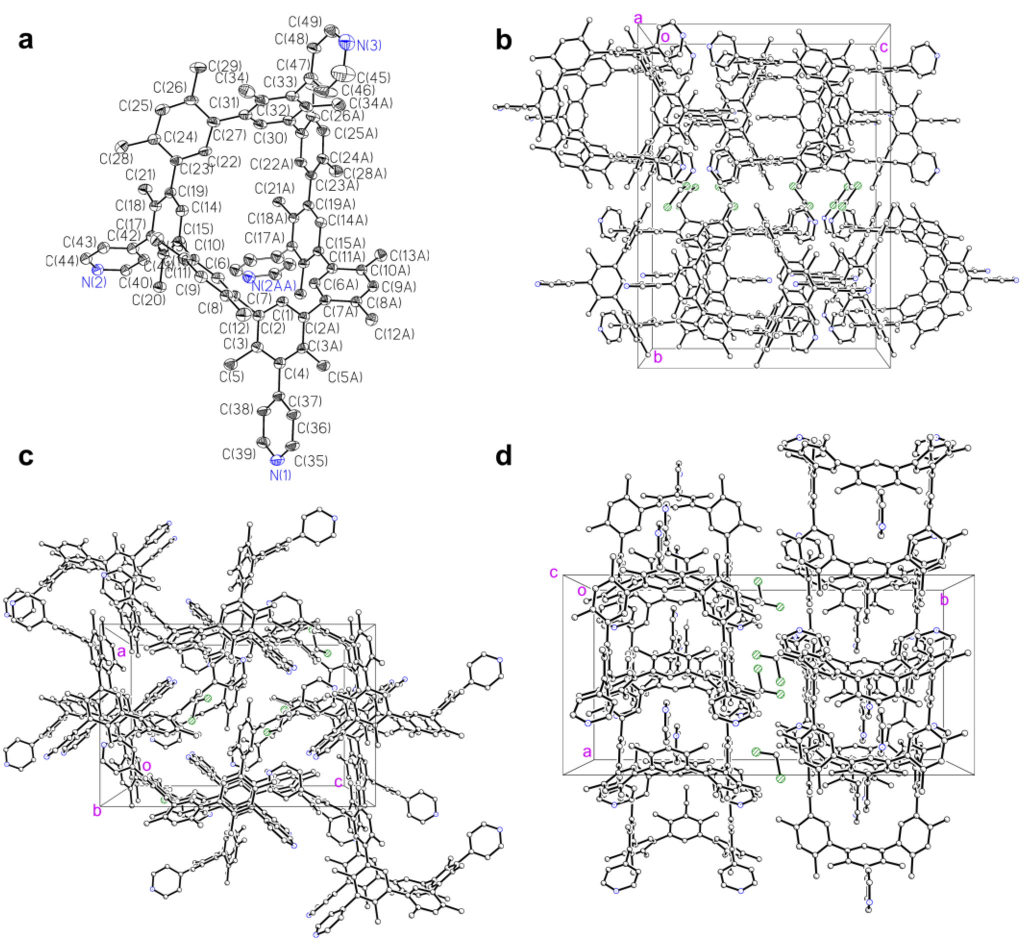

Supplementary Fig. 35 Ellipsoid form (a) and packing structure of **1** along with a (b), b (c), or c (d) axis in the single crystal structure of **1**•2.25CH_2Cl_2. Ellipsoids are drawn at 25% probability. Carbon: black, Nitrogen: bluish violet. Selected atomic distances [Å]: N(1)-N(2) 13.198(5), N(2)-N(2AA) 9.442(3), N(2) -N(3) 17.090(2), N(1)-N(3) 18.561(1). The X-ray crystallographic data of **1** have been deposited to the CCDC, with deposition numbers as 2280091.

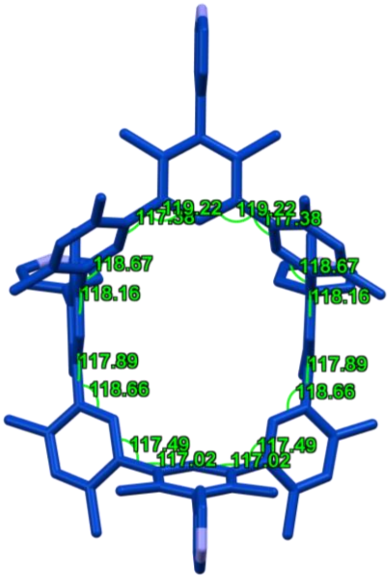

Supplementary Fig. 36 The torsion angles between the neighboring meso-dimethyl benzene units range on **1** in the single crystal structure of **1**•2.25CH_2Cl_2. Carbon: blue, Nitrogen: bluish violet.

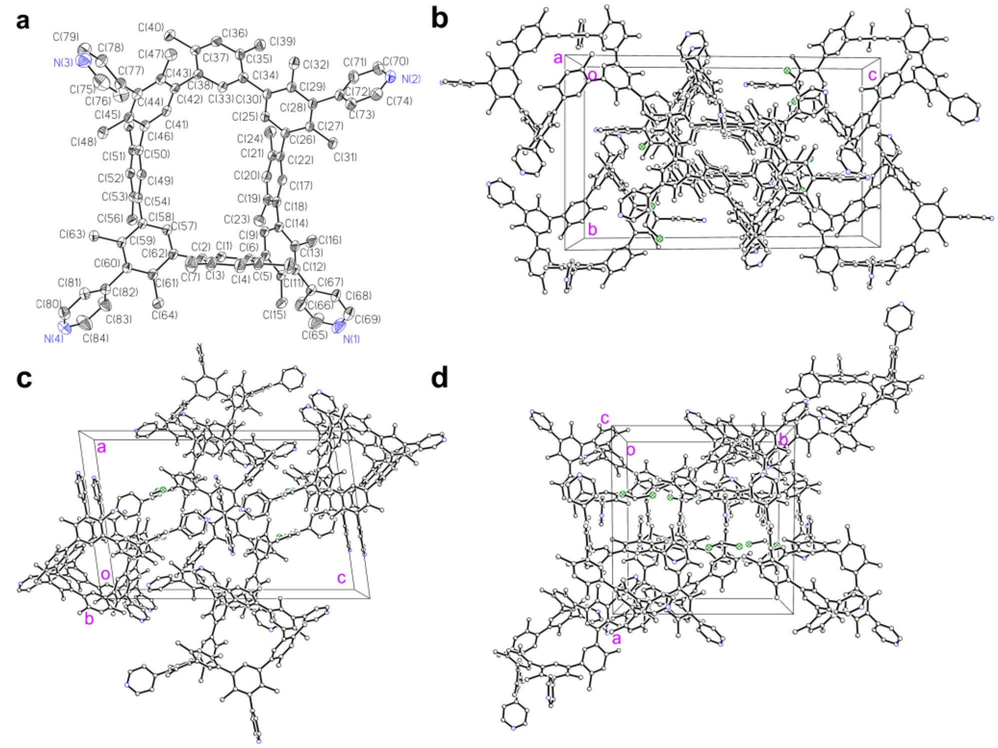

Supplementary Fig. 37 Ellipsoid form (a) and packing structure of **2** along with a (b), b (c), or c (d) axis in the single crystal structure of **2**•CH_2Cl_2•0.75H_2O. Ellipsoids are drawn at 25% probability. Carbon: black, Nitrogen: bluish violet. Selected atomic

distances [Å]: N(1)-N(2) 13.975(9), N(1)-N(3) 20.455(8), N(2) -N(3) 15.244(8), N(1)-N(4) 14.860(9). The X-ray crystallographic data of **2** have been deposited to the CCDC, with deposition numbers as 2280092.

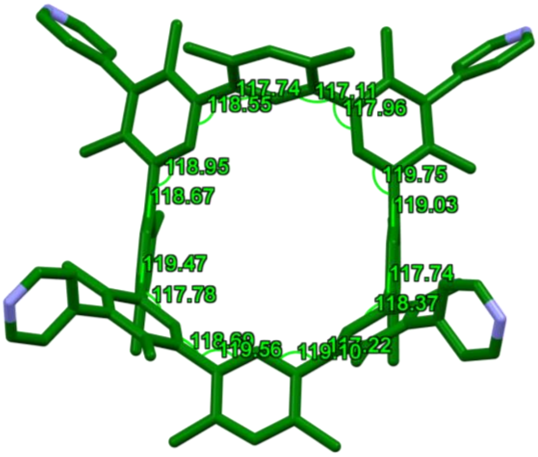

Supplementary Fig. 38 The torsion angles between the neighboring meso-dimethyl benzene units range on **2** in the single crystal structure of **2**•CH_2Cl_2•0.75H_2O. Carbon: green, Nitrogen: bluish violet.

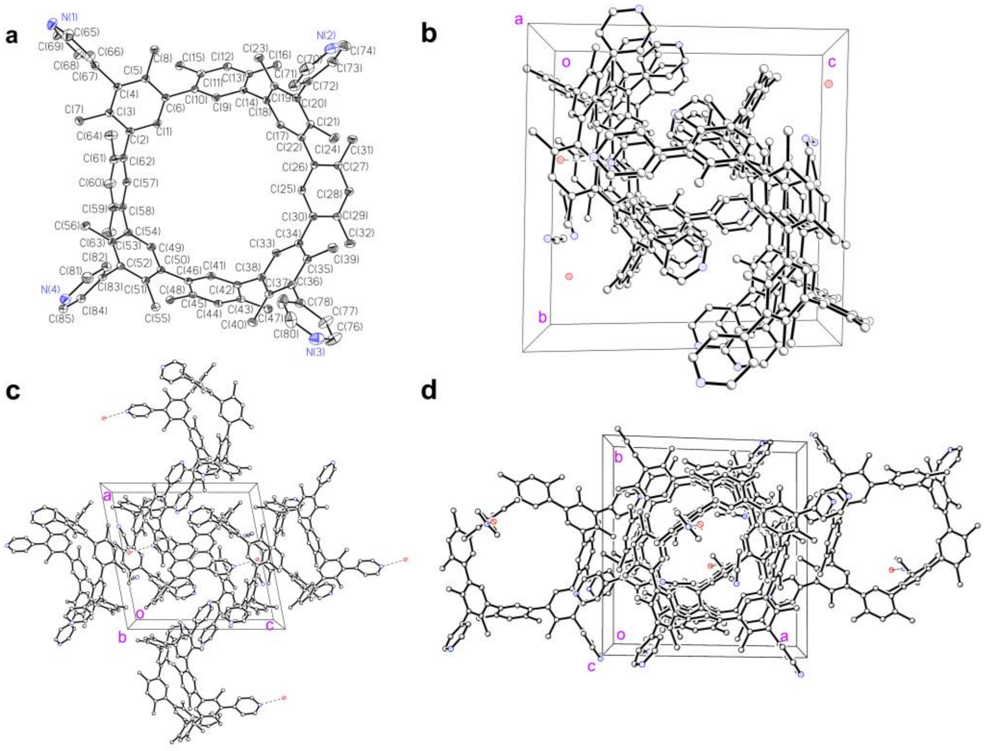

Supplementary Fig. 39 Ellipsoid form (a) and packing structure of **3** along with a (b),

b (c), or c (d) axis in the single crystal structure of **3**•$CH_3COOC_2H_5$•$2CH_3CN$•$3.5H_2O$. Ellipsoids are drawn at 25% probability. Carbon: black, Nitrogen: bluish violet. Selected atomic distances [Å]: N(1)-N(2) 13.443(7), N(1)-N(4) 18.795(1). The X-ray crystallographic data of **3** have been deposited to the CCDC, with deposition numbers as 2280089.

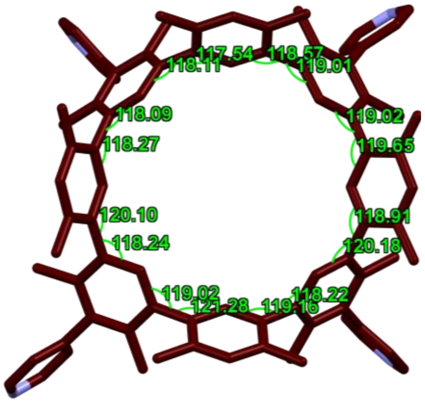

Supplementary Fig. 40 The torsion angles between the neighboring meso-dimethyl benzene units range on **3** in the single crystal structure of **3**•$CH_3COOC_2H_5$•$2CH_3CN$•$3.5H_2O$. Carbon: dark red, Nitrogen: bluish violet.

Supplementary Note 5: Thermally induced transformation processes between different atropisomers.

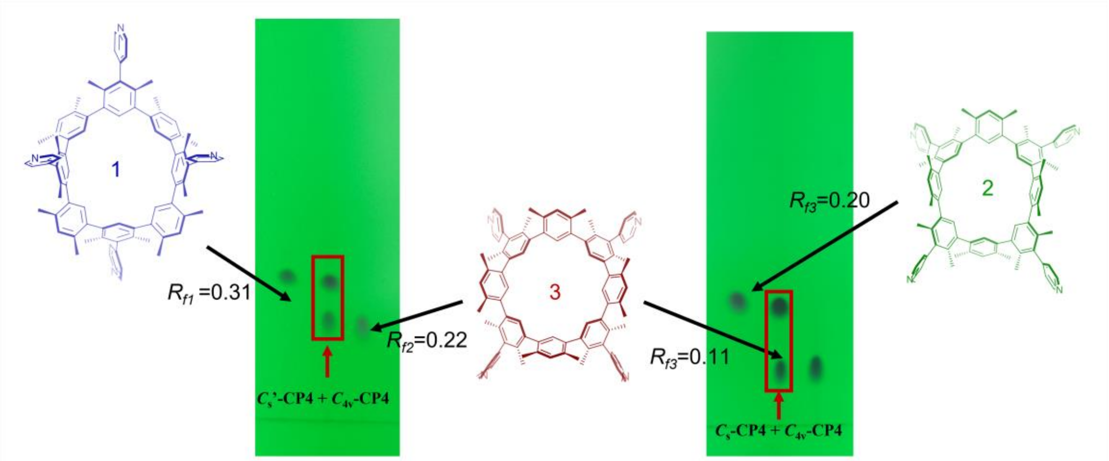

Supplementary Fig. 41 Conversion from **1** (left) or **2** (right) to **3** in solid state with NaCl substrate at 573 K. Eluent was *n*-hexane/acetone/methanol (left, 10:1:0.1, right, 10:1:0.05; *v*/*v*/*v*).

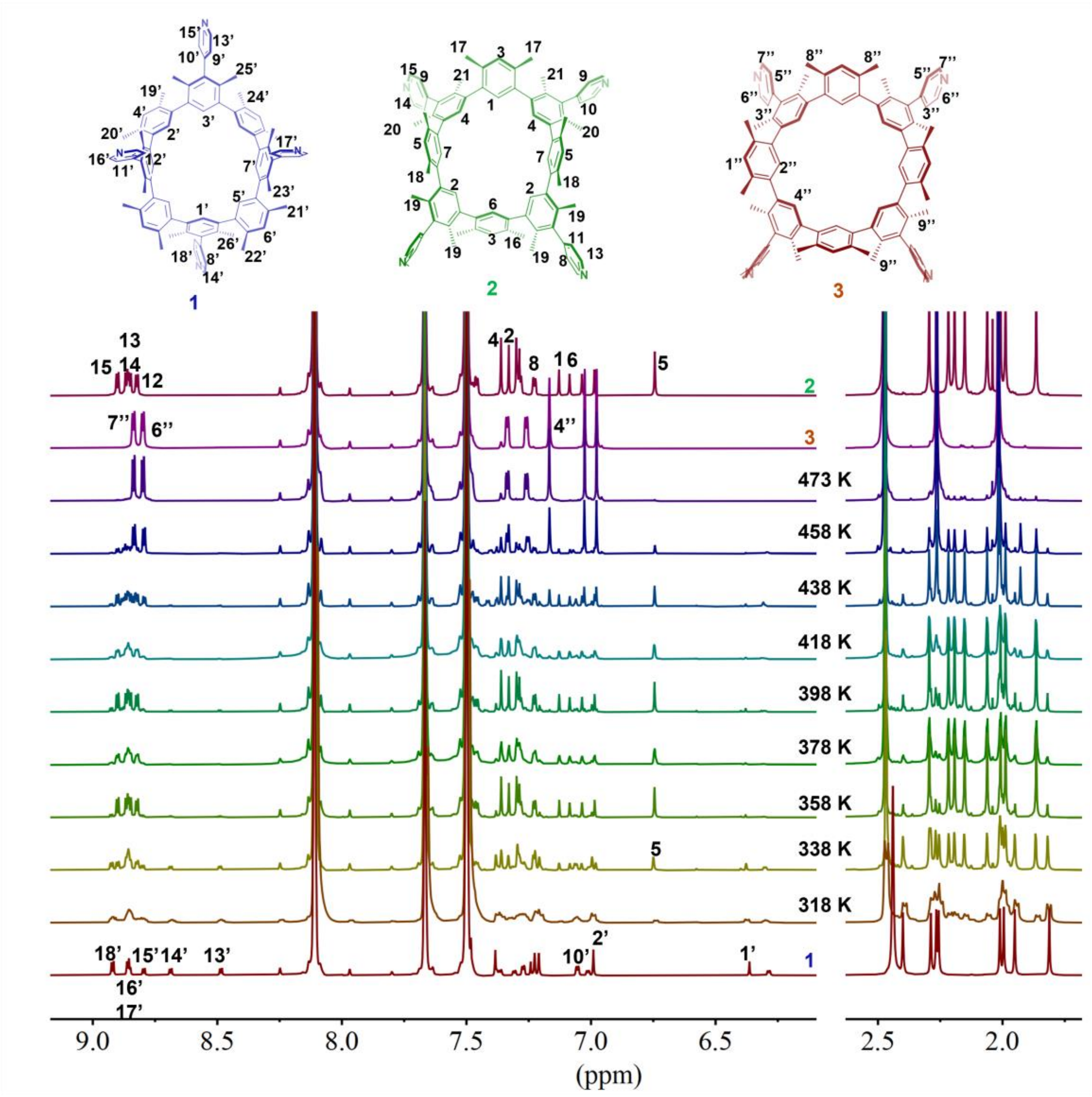

Supplementary Fig. 42 Temperature-dependent ^{1}H NMR spectra of **1** (5.3×10^{-3} M) in PhNO$_2$-d_5 (500 MHz).

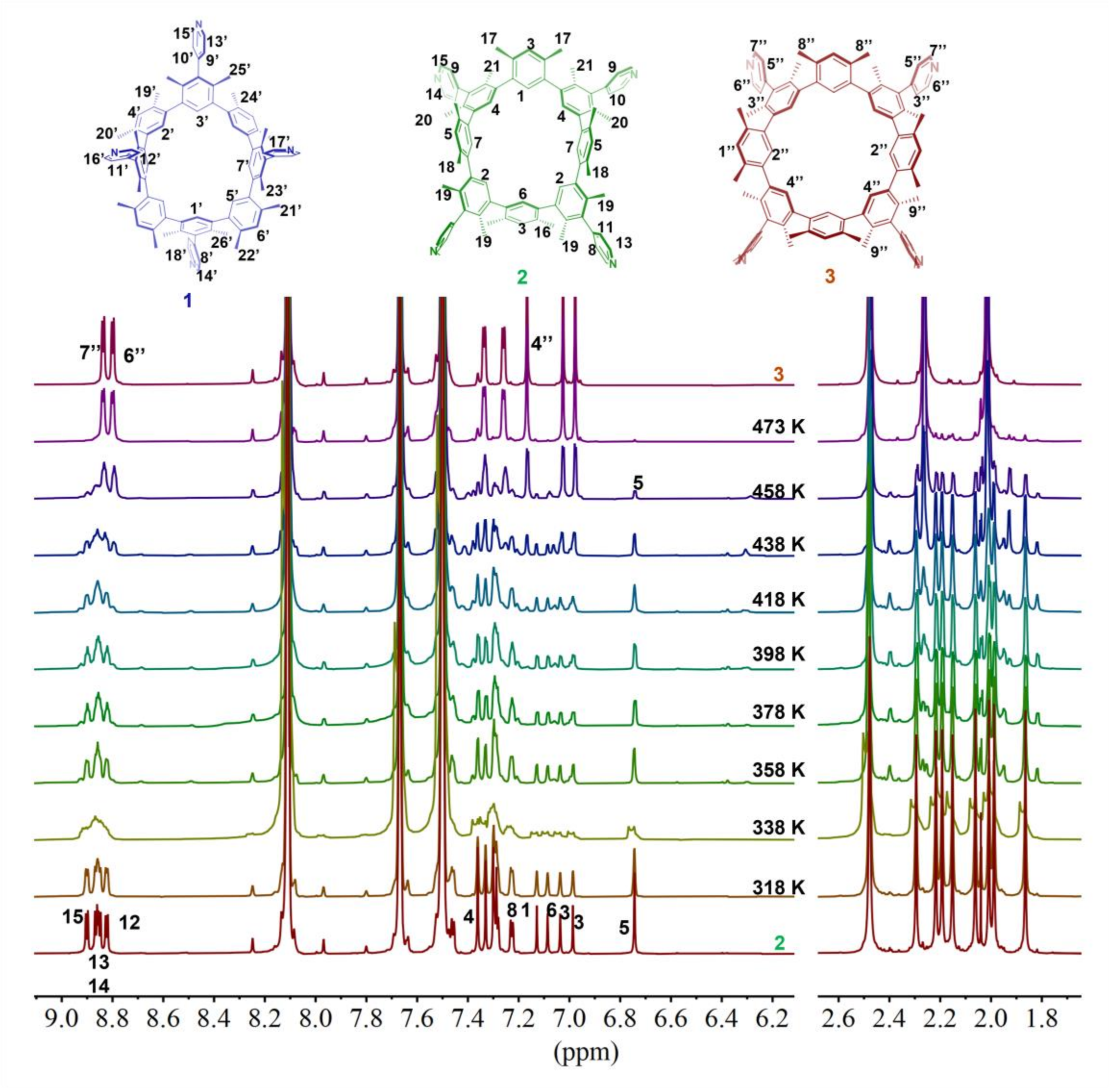

Supplementary Fig. 43 Temperature-dependent ^{1}H NMR spectra of **2** (5.3×10^{-3} M) in $PhNO_2$-d_5 (500 MHz).

Supplementary Note 6: ^{1}H NMR spectra of thermally induced interconversion processes between **1** and **2** enantiomers.

The integral areas of the characteristic peaks of **1** (**$H_{1'}$**) and **2** (**H_3**) in Supplementary Fig. 44 are carefully measured and calculated in Supplementary Fig. 46.

It was observed that **1** and **2** converted to each other at 393 K. **3** didn't involve in the reaction (Supplementary Fig. 44, 45). It is implied that the temperature is too low to induce **1** or **2** crossing the energy barrier to form **3**. Herein, the transition between **1**

and **2** can be regarded as the first-order reversible reaction, which can be expressed by the following formula (eq.1 ~ eq.3).

$$\mathbf{1} \underset{k_{-1}}{\overset{k_1}{\rightleftharpoons}} \mathbf{2}$$

$$\frac{dc_2}{dt} = k_1 c_1 - k_{-1} c_2 \qquad (2)$$

$$c_1 + c_2 = c_0 \qquad (3)$$

c_0 is the total concentration of **1** and **2**; c_1 and c_2 are the concentrations of **1** and **2**, respectively; k_1 and k_{-1} are the reaction rate constants.

Substituting eq.3 into eq.2 gives eq.4.

$$\frac{dc_2}{dt} = k_1 (c_0 - c_2) - k_{-1} c_2 \qquad (4)$$

Expression of equilibrium rate constant (K_1) of first order reversible reaction.

$$\frac{k_1}{k_{-1}} = \frac{c_2}{c_1} = K_1 \qquad (5)$$

When the reaction reaches equilibrium, eq.5 can be written as follows:

$$\frac{c_{2e}}{c_0 - c_{2e}} = \frac{k_1}{k_{-1}} = K_1 \qquad (6)$$

c_{2e} is the equilibrium concentration of **2.**

The expression k_{-1} can be obtained according to eq.6.

$$k_{-1} = k_1 \frac{c_0 - c_{2e}}{c_{2e}} \qquad (7)$$

Substituting eq.7 into eq. 4 gives eq. 8.

$$\frac{dc_2}{dt} = k_1 (c_0 - c_2) - k_1 \frac{(c_0 - c_{2e})}{c_{2e}} c_2$$

$$= \frac{k_1 c_0 (c_{2e} - c_2)}{c_{2e}} \qquad (8)$$

By calculus derivation of formula eq.8, the expression formula of c_2 can be obtained as (eq.9).

$$c_2 = c_{2e} - c_{2e} e^{\frac{-t k_1 c_0}{c_{2e}}} \qquad (9)$$

Take c_2 as the vertical axis and time as the horizontal axis of the plot, and use the graph method to obtain the first-order derivative of the curve (dc_2/dt). Then ((dc_2/dt)/c_2) as the vertical coordinate, c_1/c_2 as the horizontal coordinate to the plot, and fit the linear equation related to $y = ax + b$ (Supplementary Fig. 46b). The slope of the equation was the reaction rate $k_1 = (1.4 \pm 0.1) \times 10^{-2}$ s^{-1}, and the intercept was the reaction rate $k_{-1} = -(2.3 \pm 0.1) \times 10^{-3}$ s^{-1}. Substituting them into eq.9, the equation of c_2 concerning t can be obtained. This equation can be used to obtain the theoretical data value which is consistent with the experimental data.

Similarly, the function equation eq.10 of c_1 about time t can be obtained:

$$c_1 = c_0 - c_2 = c_0 - c_{2e} + c_{2e} e^{\frac{-t k_1 c_0}{c_{2e}}} \qquad (10)$$

By substituting time t into the equation, the data consistent with the experimental data can be obtained, and the curve fitted by this formula is highly coexisting with the experimental data (Supplementary Fig. 41a).

Substituting the values of k_1, k_{-1}, and T into eq.5, eq.11 gives the Gibbs free energy ΔG_1^{θ} of this reaction:

$$\Delta G_1^{\theta} = -RT\ln K_1 \qquad (11)$$

K_1 is the equilibrium constant of the reaction; k_1 *and* k_{-1} are positive and reverse reaction rate constants respectively; R is the universal gas constant; T is the temperature; ΔG_1^{θ} is the Gibbs free energy.

Substituting the values of k_1, k_{-1}, and T into eq.5, eq.12gives the Gibbs activation free energy -$\Delta G_1^{\neq}$ of this reaction.

$$k_1 = \kappa \frac{k_B T}{h} \exp \frac{-\Delta G_1^{\neq}}{RT} \qquad (12)$$

R is the universal gas constant; T is the temperature; k_1 is the reaction rate constant; κ is the transition coefficient, generally regarded as a unit; k_B is Boltzmann constant; h is Planck constant; $-\Delta G_1^{\neq}$ is the Gibbs activation free energy.

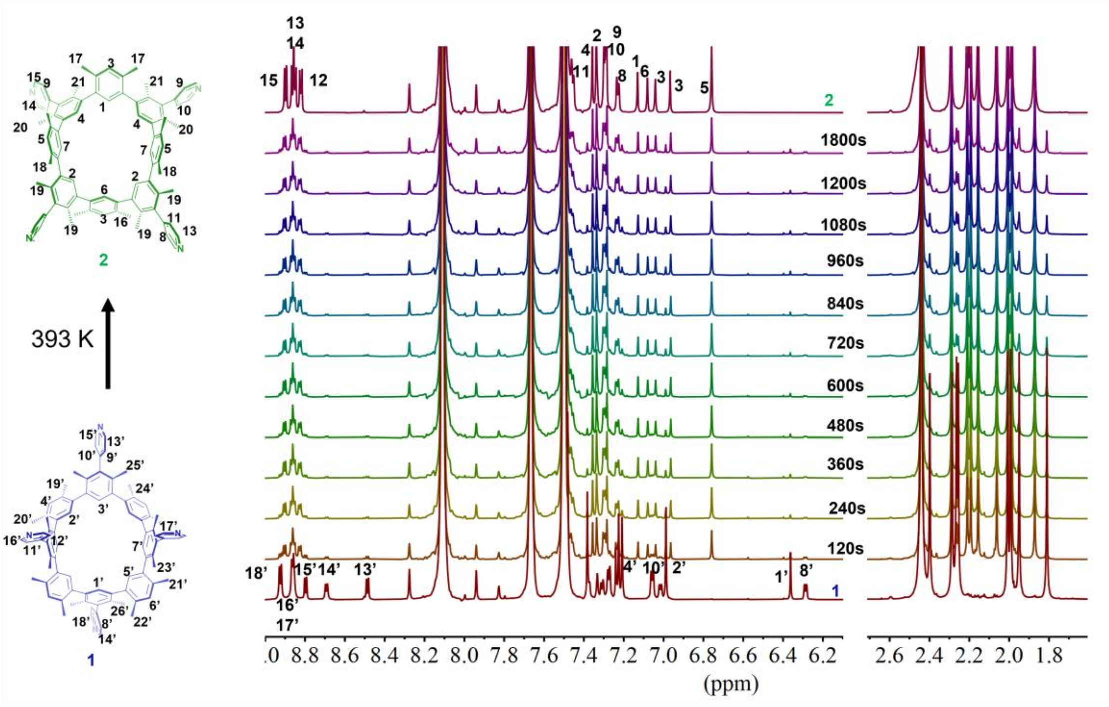

Supplementary Fig. 44 Time-dependent ^{1}H NMR spectra of **1** (5.3×10^{-3} M) in PhNO$_2$-d_5 at 393 K (500 MHz).

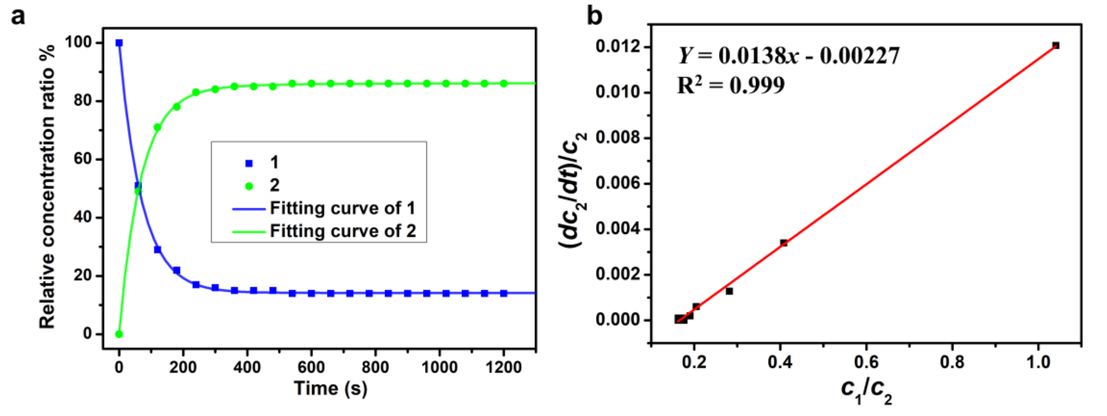

Supplementary Fig. 45 (a) Time-dependent relative concentration ratio changes of **1** (blue dot) to **2** (green dot) in PhNO$_2$-d_5 at 393 K. (b) Linear fitting graph for $(dc_2/dt)/c_2$

and c_1/c_2.

Supplementary Table 3. Reaction rate constants (k_1, k_{-1}), Gibbs free energy ($\Delta G^{\theta}_{1(393\ K)}$), Gibbs activation free energy ($\Delta G^{\neq}_{1(393\ K)}$) for the **1** to **2** at 393 K.

k_1	k_{-1}	$\Delta G^{\theta}_{1(393\ K)}$	$\Delta G^{\neq}_{1(393\ K)}$
(1.4 ± 0.1) × 10^{-2} s^{-1}	-(2.3 ± 0.1) × 10^{-3} s^{-1}	-5.9 ± 0.2 kJ mol^{-1}	111 ± 6 kJ mol^{-1}

Meanwhile, after heating **2** in $PhNO_2$-d_5 at 393 K for 1 min, the proton signal of **1** appeared (Supplementary Fig. 45). The reaction also reached dynamic equilibrium, and the relative concentration ratio c_2:c_1 = 87: 13.

The integral areas of characteristic peaks of **1** (Supplementary Fig. 45, $H_{2'}$) and **2** (Supplementary Fig. 45, H_2) were measured carefully. The calculation method shown above (i.e., eq.1~eq.12) was used to give out the non-linear fitting curve highly consistent with the experimental data (Supplementary Fig. 47).

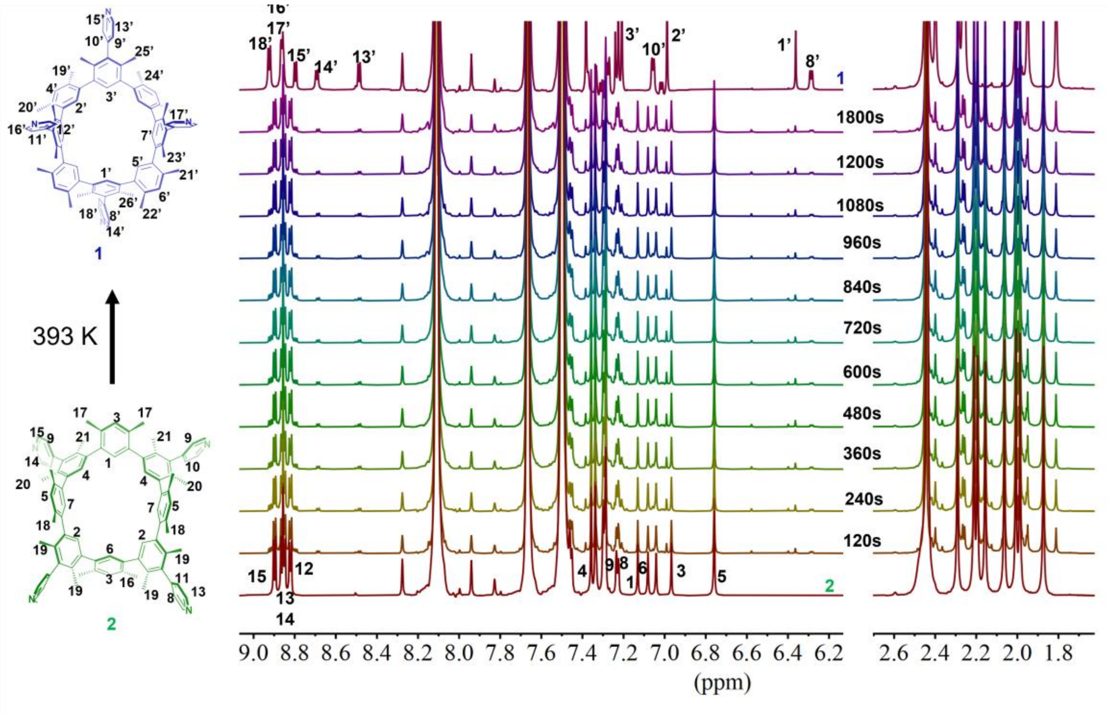

Supplementary Fig. 46 Time-dependent ^{1}H NMR spectra of **2** (5.3 × 10^{-3} M) in

$PhNO_2$-d_5 at 393 K (500 MHz).

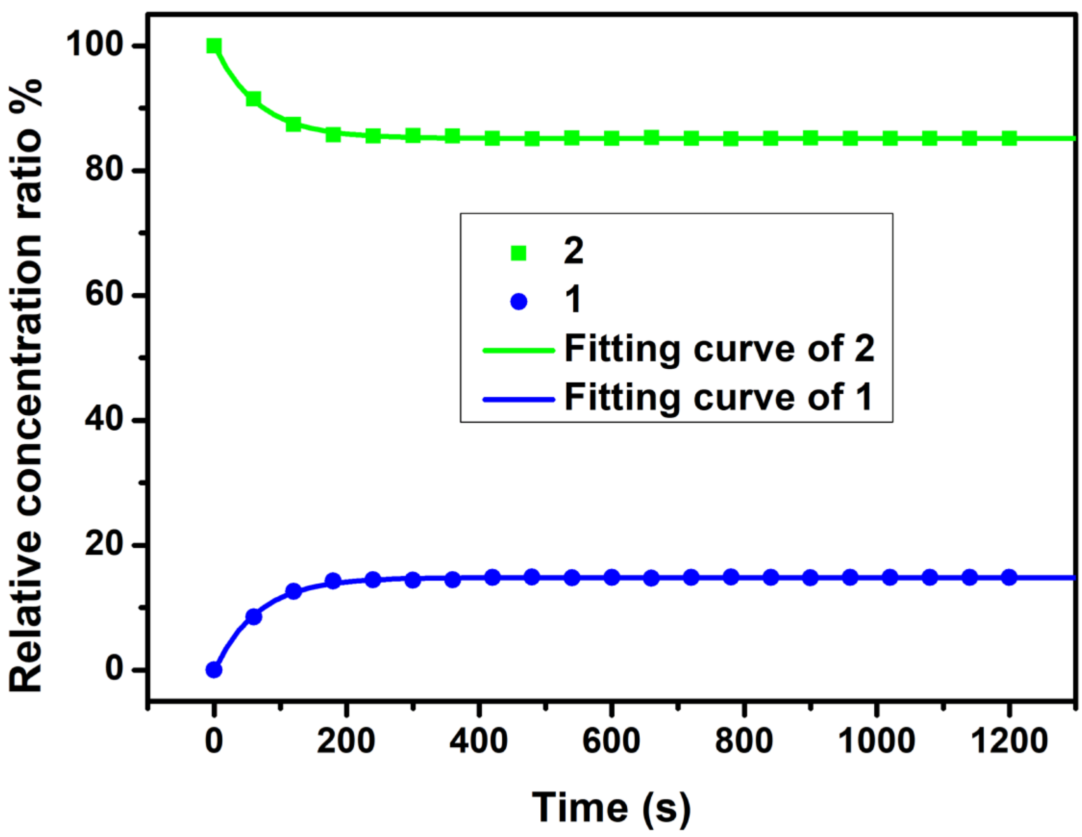

Supplementary Fig. 47 Time-dependent relative concentration ratio changes of **2**(green dot) to **1** (blue dot) in $PhNO_2$-d_5 at 393 K.

According to the above equilibrium, the thermal dynamics and kinetic parameters of the transition were calculated using time-dependent ^{1}H NMR data in TCE-d_2 at 393 K. These parametes include the reaction rate constant (k_1 as $(5.2 \pm 0.4) \times 10^{-3}$ s^{-1}, k_{-1} as -$(1.4 \pm 0.1) \times 10^3$ s^{-1}), the Gibbs activated free energy ($\Delta G^{\neq}_{1(393\ K)}$ as 114 ± 6 kJ mol^{-1}), the equilibrium constant (K_1 as 3.7 ± 0.2), and the Gibbs free energy ($\Delta G^{\theta}_{1(393\ K)}$ as -4.4 ± 0.2 kJ mol^{-1}).

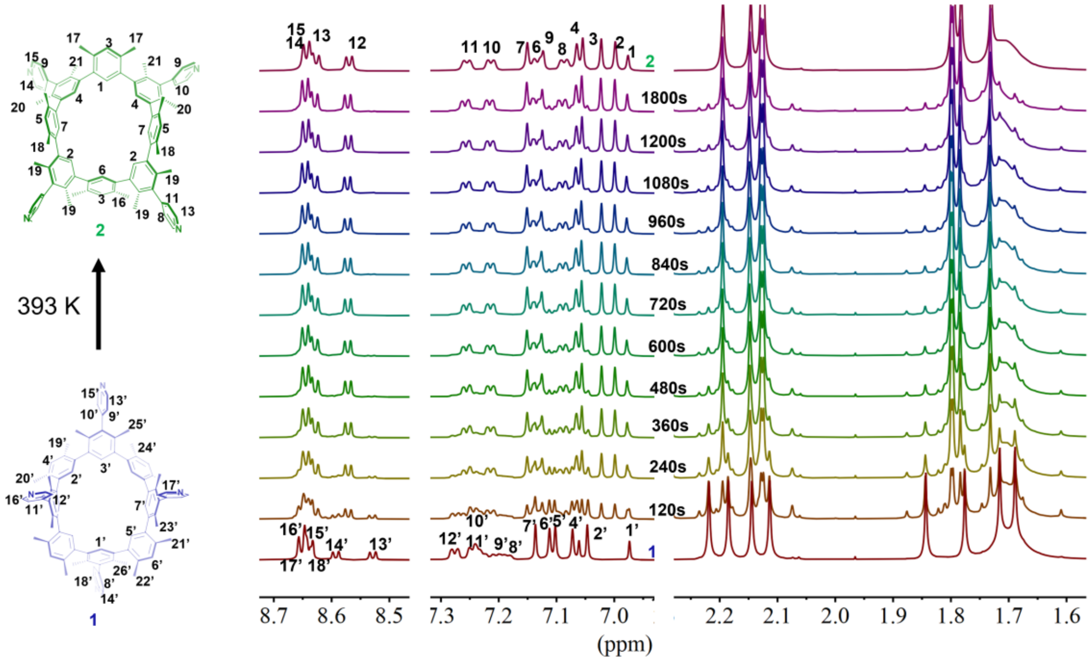

Supplementary Fig. 48 Time-dependent ^{1}H NMR spectra of **1** (5.3×10^{-3}M) in TCE-d_2 at 393 K (500 MHz).

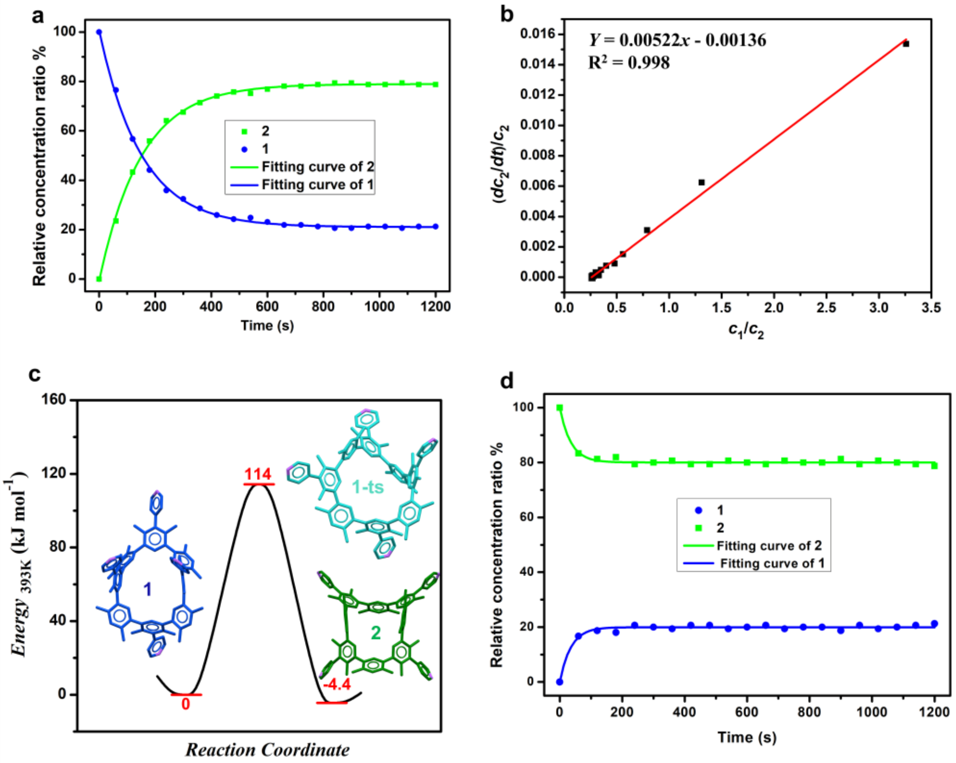

Supplementary Fig. 49 (a) Time-dependent relative concentration ratio changes of **1**

(blue dot) and **2** (green dot) in **1** (5.3×10^{-3} M) dissolved in TCE-d_2 at 393 K. (b) Linear fitting graph for $(dc_2/dt)/c_2$ and c_1/c_2. (c) The potential energy diagram of the conversion between **1** and **2** in TCE-d_2 at 393 K. The transition state is listed based on theoretical calculation. The relative energy values were calculated with the free energy of **1** as standard (0 kJ mol^{-1}). (d) Time-dependent relative concentration ratio changes of **1** (blue dot) and **2** (green dot) in **2** (5.3×10^{-3} M) dissolved in TCE-d_2 at 393 K.

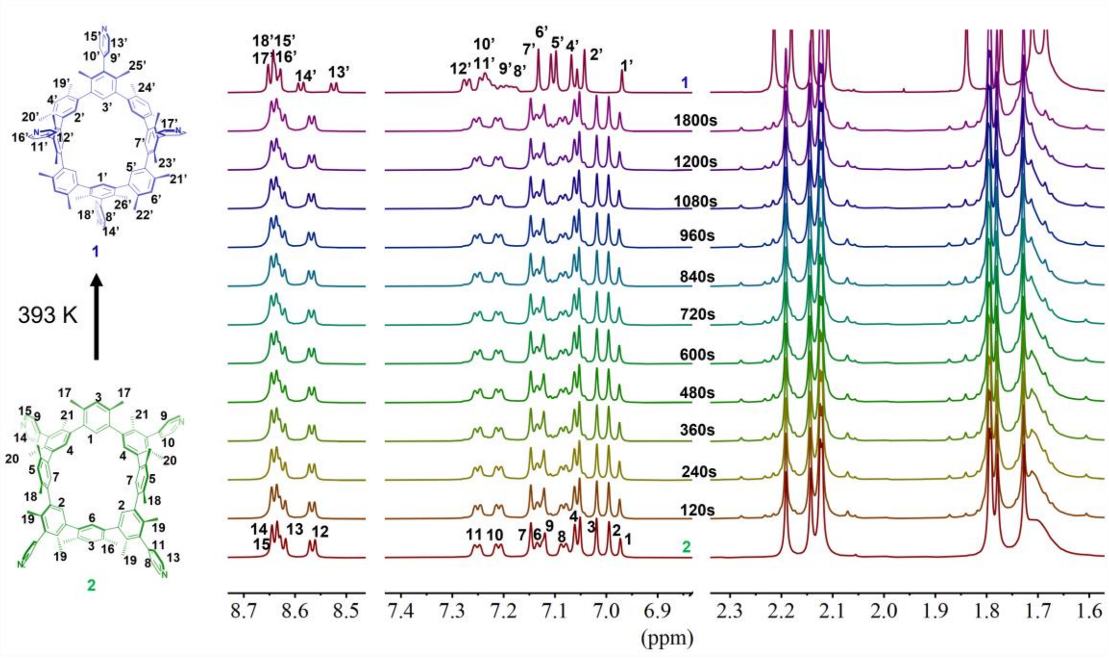

Supplementary Fig. 50 Time-dependent ^{1}H NMR spectra of **2** (5.3×10^{-3}M) in TCE-d_2 at 393 K (500 MHz).

Supplementary Note 7: ^{1}H NMR spectra of thermally induced interconversion processes from **1** or **2** to **3**.

The integral areas of characteristic peaks of **1** ($H_{1'}$), **2** (H_5), and **3** ($H_{4''}$) were measured in this case (Supplementary Fig. 51). The relative proportions of the three atropisomers are further obtained (Supplementary Fig. 52).

At 473 K, the conversion from **1** to **2** can be completed very quickly, and both are converted to **3**. This conversion process between the three isomers of macrocycle was considered a combination of two reversible reactions. One is a fast conversion process between **1** and **2**. Another is a slow conversion process from **2** to **3**.

$$1 \underset{k_{-1}}{\overset{k_1}{\rightleftharpoons}} 2 \underset{k_{-2}}{\overset{k_2}{\rightleftharpoons}} 3$$

fast slow

$$\frac{k_1}{k_{-1}} = \frac{c_{2e}}{c_{1e}} = K_1, \quad \frac{k_2}{k_{-2}} = \frac{c_{3e}}{c_{2e}} = K_2 \qquad (13)$$

Herein, the conversion between **1** and **2** is too fast to be well characterized with the whole time dependent specctra. c_{1e} is the equilibrium concentration of **1**, c_{2e} is the equilibrium concentration of **2**, c_{3e} is the equilibrium concentration of **3**. K_1 or K_2 is corresponding equilibrium constant. But the two isomers are distinguishable in the NMR time scale, eq.14~eq.17 were used to calculate the reaction rate constant k_1 of this reaction.

$$\frac{P_2 - P_1}{P_1 + P_2} = \Delta P = \left(\frac{X^2 - 2}{3}\right)^{3/2} \frac{1}{X} \qquad (14)$$

$$X = 2\pi\tau\Delta v \qquad (15)$$

$$\frac{1}{\tau} = \frac{1}{\tau_1} + \frac{1}{\tau_2} \qquad (16)$$

$$k_1 = \frac{1}{2\tau}(1 - \Delta P) \qquad (17)$$

P_1 and P_2, the relative abundances of species **1** and **2** can be obtained via integrating the spectra, and the chemical shift difference Δv between specific corresponding proton(s) on **1** and **2** can be measured from the spectra. τ is the average lifespan, τ_1 is the lifespan of species **1**, τ_2 is the lifespan of species **2**. Plugging these parameters into the above formula gives k_1 as $(8.0 \pm 0.4) \times 10^{-2}$ s^{-1}.

Before the signal of **2** disappears, the slow conversion process from **2** to **3** can be investigated with eq.18~eq.31.

$$\frac{dc_3}{dt} = k_2 c_2 - k_{-2} c_3 \qquad (18)$$

$$c_1 + c_2 + c_3 = c_0 \qquad (19)$$

c_0 is the total concentration. c_1, c_2, and c_3 are the concentrations of **1**, **2**, and **3** respectively, and k_2 and k_{-2} are the reaction rate constants.

Since the concentration ratio between **1** and **2** kept as $c_2/c_1 = 7/2$ (i.e., $c_1 + c_2 = \frac{9}{7}c_2 = \mathrm{K}c_2$). It means

$$\mathrm{K}c_2 + c_3 = c_0 \qquad (20)$$

The expression c_2 can be obtained according to eq.20, namely eq.19 can be simplified as eq.21:

$$c_2 = \frac{c_0 - c_3}{\mathrm{K}} \qquad (21)$$

Substituting eq.21 into eq.18 gives eq.22:

$$\frac{dc_3}{dt} = \frac{k_2(c_0 - c_3)}{\mathrm{K}} - k_{-2}c_3 \qquad (22)$$

Suppose: $k_3 = \frac{k_2}{\mathrm{K}}$, substituting k_3 into eq.22 gives the following formula eq.23:

$$\frac{dc_3}{dt} = k_3c_0 - k_3c_3 - k_{-2}c_3$$
$$= k_3c_0 - (k_3 + k_{-2})c_3 \qquad (23)$$

Suppose: $k_4 = k_3 + k_{-2}$, substituting k_4 into eq.23 gives the following formula eq.24:

$$\frac{dc_3}{dt} = k_3c_0 - k_4 \qquad (24)$$

With formula eq.24, the expression formula of c_3 can be obtained as (eq.26).

$$t = \frac{-\ln(k_3c_0 - k_3c_3)}{k_4} + \mathrm{C}_1 \qquad (25)$$

$$c_3 = \frac{k_3c_0 - \mathrm{b}e^{-k_4t}}{k_4} \qquad (26)$$

C_1, b for integral constant

Take c_3 as the vertical axis and time as the horizontal axis of the plot, and use the

graph method to obtain the first-order derivative of the curve (dc_3/dt). Then ($dc_3/dt/c_3$) as the vertical coordinate, c_2/c_3 as the horizontal coordinate to the plot, and fit the linear equation related to y= ax + b. (Supplementary Fig. 52 b). The slope of the equation was k_2 = (2.1 ± 0.1) × 10^{-3} s^{-1}, and the intercept was k_{-2} = -(7.9 ± 0.1) × 10^{-5} s^{-1}. The curve of the theoretical data can be obtained by substituting the data of t, k_2, and k_{-2} into eq.26, and the experimental data are consistent (Supplementary Fig. 52a,).

The expression c_3 can be obtained as eq.27 according to eq.20:

$$c_3 = c_0 - \mathrm{K}c_2 \qquad (27)$$

Substituting eq.27 into eq.18 gives eq.28:

$$\frac{dc_2}{dt} = -k_2c_2 + k_{-2}c_3$$

$$= (-k_2 - \mathrm{K}k_{-2})c_2 + k_{-2}c_0 \qquad (28)$$

Suppose: $k_5 = k_2 + \mathrm{K}k_{-2}$, substituting k_5 into eq.23 gives eq.29:

$$\frac{dc_2}{dt} = k_{-2}c_0 - k_5c_2 \qquad (29)$$

Derivation of formula eq.29 created the expression formula of c_3 as (eq.31).

$$t = -\frac{\ln(k_{-2}c_0 - k_5c_2)}{k_5} + \mathrm{C}_2 \qquad (30)$$

$$c_2 = \frac{k_{-2}c_0 - \mathrm{m}e^{-k_5t}}{k_5} \qquad (31)$$

C_2, m for integral constant

Substituting k_2, k_{-2}, t into eq.32, the theoretical data is consistent with the experimental data of **2** (Supplementary Fig. 52a).

Substituting the values of k_1, k_2,and T into eq.10 and eq.11, gives the Gibbs free energy $\Delta G^{\theta}_{1(473\ \mathrm{K})}$, $\Delta G^{\theta}_{2(473\ \mathrm{K})}$, By substituting the values of k_1 or k_{-2} into eq.11, the Gibbs activation free energy $\Delta G^{\neq}_{1(473\ \mathrm{K})}$, $\Delta G^{\neq}_{2(473\ \mathrm{K})}$ can be obtained, respectively

(Supplementary Table 4).

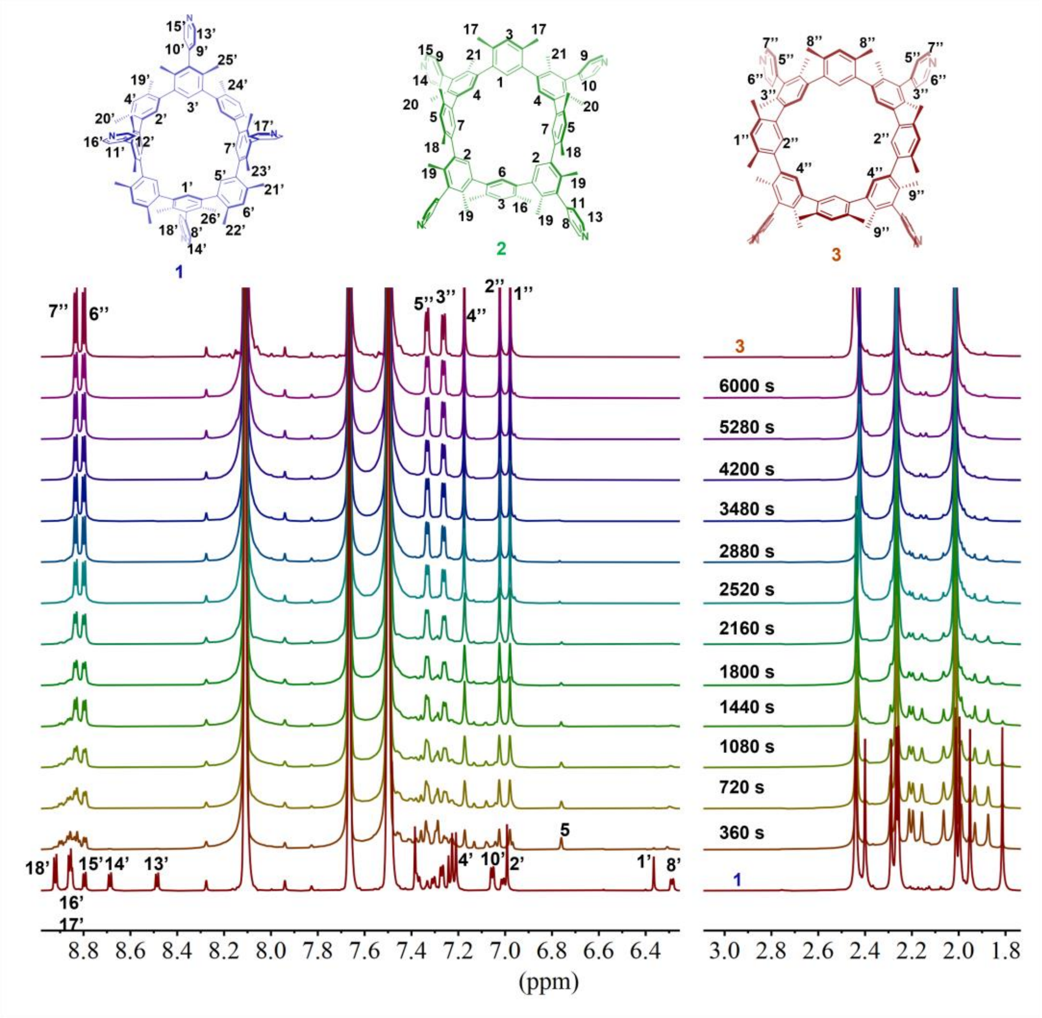

Supplementary Fig. 51 Time-dependent ^{1}H NMR spectra of **1** (5.3×10^{-3} M) in $PhNO_2$-d_5 at 473 K (500 MHz).

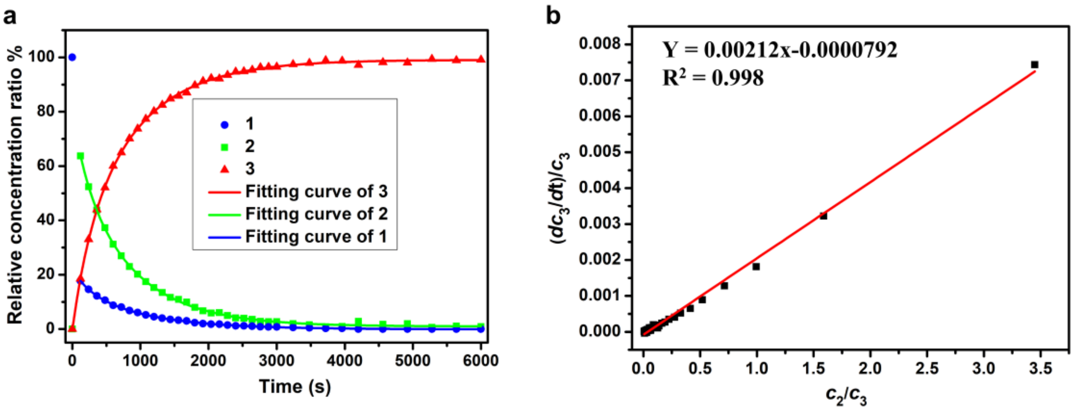

Supplementary Fig. 52 (a) Time-dependent relative concentration ratio changes of **1,**

2 and **3** in **1** (5.3×10^{-3} M) dissolved in PhNO$_2$-d_5 at 473 K (**1** (blue dot), **2** (green dot), and **3** (red dot)). (b) Linear fitting graph for(dc_3/dt)/c_3 and c_2/c_3.

Supplementary Table 4. Reaction rate constants (k_1, k_{-1}, k_2, k_{-2}), Gibbs free energy ($\Delta G^{\theta}_{1(473\ K)}$, $\Delta G^{\theta}_{2(473\ K)}$), Gibbs activation free energy ($\Delta G^{\neq}_{1(473\ K)}$, $\Delta G^{\neq}_{2(473\ K)}$) for the **1** to **3** at 473 K.

**1 to 2**			
k_1 (s^{-1})	k_{-1} (s^{-1})	$\Delta G^{\theta}_{1(473\ K)}$	$\Delta G^{\neq}_{1(473\ K)}$
$(8.0 \pm 0.4) \times 10^{-2}$	$-(2.3 \pm 0.1) \times 10^{-2}$	-4.3 ± 0.3 kJ mol^{-1}	128 ± 6 kJ mol^{-1}
**2 to 3**			
k_2 (s^{-1})	k_{-2} (s^{-1})	$\Delta G^{\theta}_{2(473\ K)}$	$\Delta G^{\neq}_{2(473\ K)}$
$(2.1 \pm 0.1) \times 10^{-3}$	$-(7.9 \pm 0.1) \times 10^{-5}$	-13 ± 1 kJ mol^{-1}	142 ± 7 kJ mol^{-1}

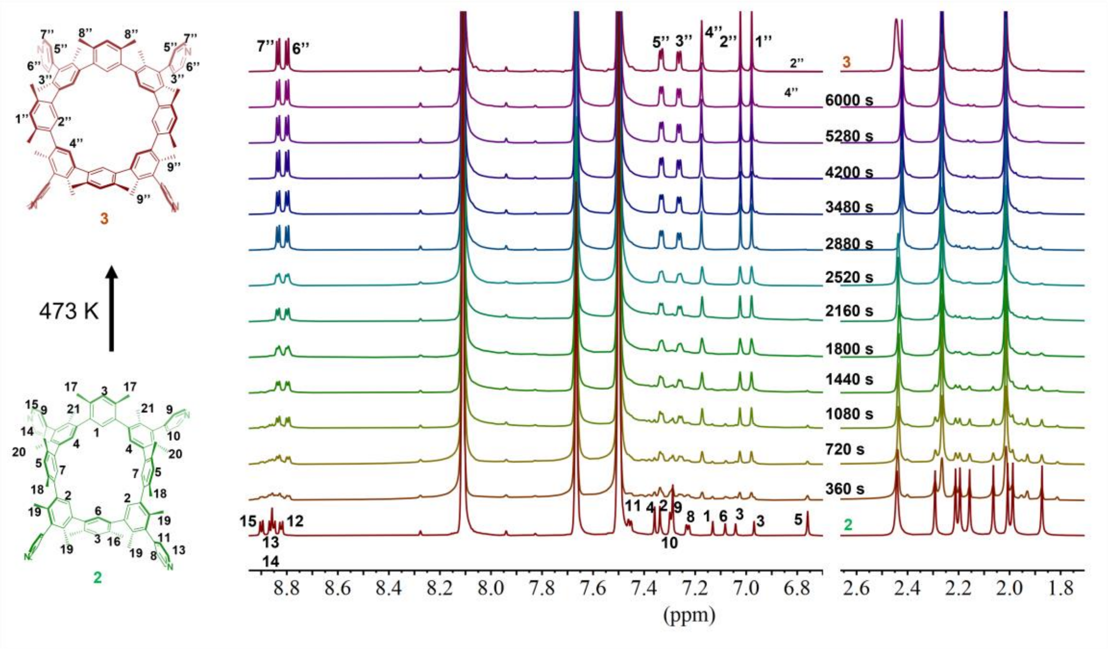

Supplementary Fig. 53 Time-dependent ^{1}H NMR spectra of **2** (5.3×10^{-3} M) in PhNO$_2$-d_5 at 473 K (500 MHz).

Supplementary Note 8: Theoretical calculation of the conformation transformation process from **1** to **3.**

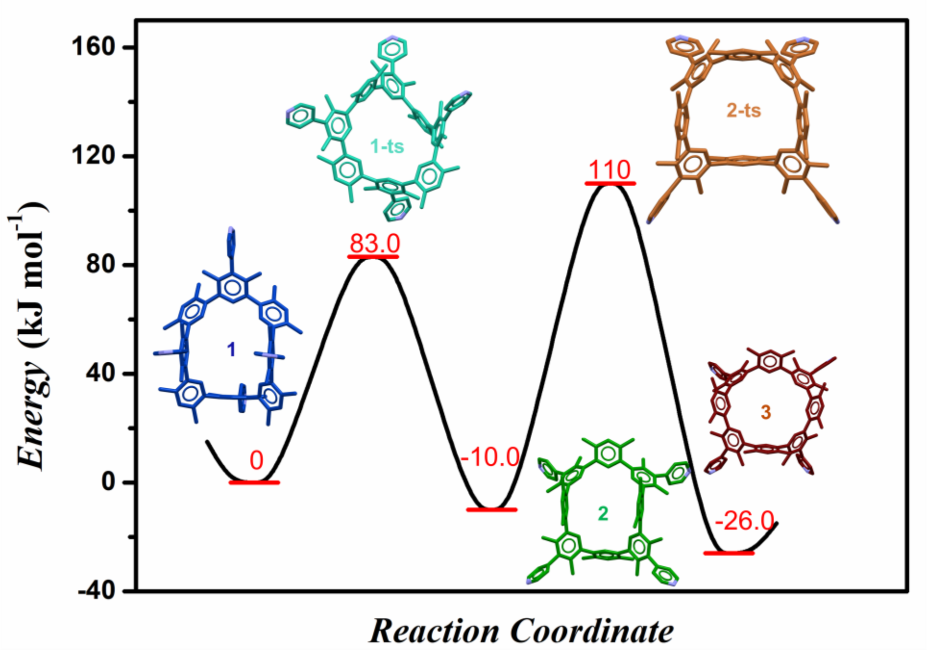

Supplementary Fig. 54 The optimized geometries and relative-energy (*E*) profiles correspond to the conversion from **1** to **3**. **1-ts** is the transition state in the conversion from **1** to **2**. **2-ts** is the the transition state in the conversion from **2** to **3**. Their optimized geometries and relative energy profiles were calculated via semiempirical methods (PM7) in MOPAC program[6]. All the hydrogen atoms have been omitted for clarity.

Supplementary Table 5. The energy (*E*) and relative energy (ΔE) of the species in the conversion process from **1** to **3**. The ΔE values were calculated with the formation energty of **1** as standard 0 kJ mol^{-1} at 473 K.

Species / Energy	**1**	**1-ts**	**2**	**2-ts**	**3**
E (kJ mol^{-1})	-241674	-241591	-241684	-241564	-241700
ΔE (kJ mol^{-1})	0	83	-10	110	-26

Supplementary Note 9: X-ray crystallography of single crystal structure of **4**.

Supplementary Table 6. X-ray crystallographic data of **4**.

**4**			
6(**3**)·12$PdCl_2$·69C_2H_6SO			
CCDC No.	2280095	Empirical formula	C_{642} H_{870} Cl_{24} N_{24} O_{69} Pd_{12} S_{69}
Formula weight	14367.31	Reflections collected	116261
Temperature/K	169.99(14)	Crystal system	tetragonal
Space group	P 42/m n m	*a* [Å]	30.115(4)
b [Å]	30.115(4)	*c* [Å]	53.271(11)
α [deg]	90.00	*β* [deg]	90.00
γ [deg]	90.00	Volume/ [$Å^3$]	48313(17)
Z	2	ρ_{calc} (g/cm^3)	0.988
Absorption coefficient/mm^{-1}	4.133	F(000)	15012
crystal size (mm^3)	0.150 × 0.100 × 0.050	Radiation	Cu-Kα (λ = 1.54184)
2Θ range for data collection/°	5.314 to 124.992	Index ranges	$-32 \leq h \leq 31$ $-34 \leq k \leq 26$ $-53 \leq l \leq 61$
Independent reflections	5925 [R_{int} = 0.1407, R_{sigma} = 0.0828]	Data/restraints/parameters	19792/520/762
Goodness-of-fit on F^2	1.012	Final R indexes [I>=2σ (I)]	R_1 = 0.0775 wR_2 = 0.2039
Final R indexes [all data]	R_1 = 0.1770 wR_2 = 0.2652	Largest diff. peak/hole/e $Å^{-3}$	0.64/-0.49

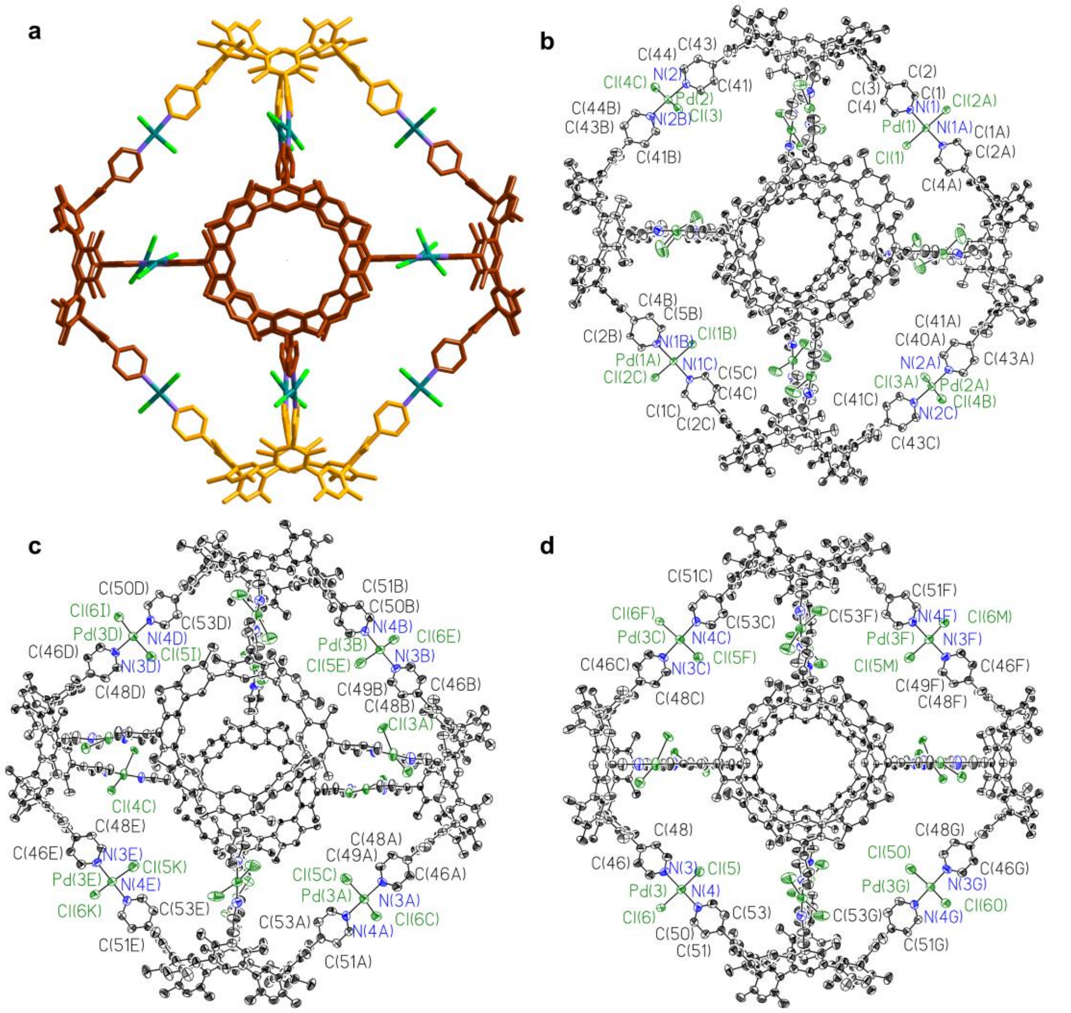

Supplementary Fig. 55 Single crystal X-ray diffraction structures of **4** (a main view). The ellipsoid form of the three views of **4** is drawn according to a ((b) top view, (c) main view, (d) left view). Displacement ellipsoids are scaled to the 20% probability level. All the other molecules and atoms have been omitted for clarity. Selected atomic distances [Å]: N(1)-Pd(1) 2.056(5), N(2)-Pd(2) 2.045(5), N(3)-Pd(3) 2.053(7), N(4)-Pd(3) 2.037(7). Selected atomic angle [°]: Cl(1)-Pd(1)-N(1) 91.13, Cl(2A)-Pd(1)-N(1) 88.96, Cl(3)-Pd(2)-N(2) 89.42, Cl(4B)-Pd(2A)-N(2C) 90.85, Cl(5)-Pd(3)-N(4) 91.49, Cl(5)-Pd(3)-N(3) 88.92, Cl(6)-Pd(3)-N(4) 90.58, Cl(6)-Pd(3)-N(3) 88.98. The X-ray crystallographic data of **4** have been deposited to the CCDC, with deposition numbers as 2280095.

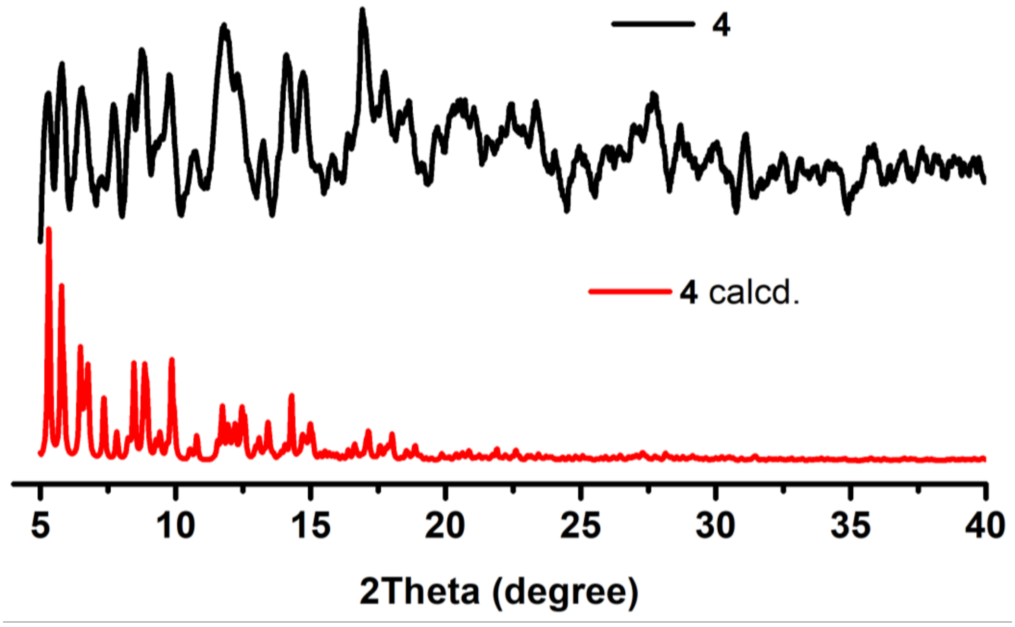

Supplementary Fig. 56 PXRD patterns of **4** (black line) and simulation curve obtained from single crystal data (red line), respectively.

Supplementary Note 10: ^{1}H NMR spectroscopic titration of **1**, or **2** with **$(PhCN)_2PdCl_2$**.

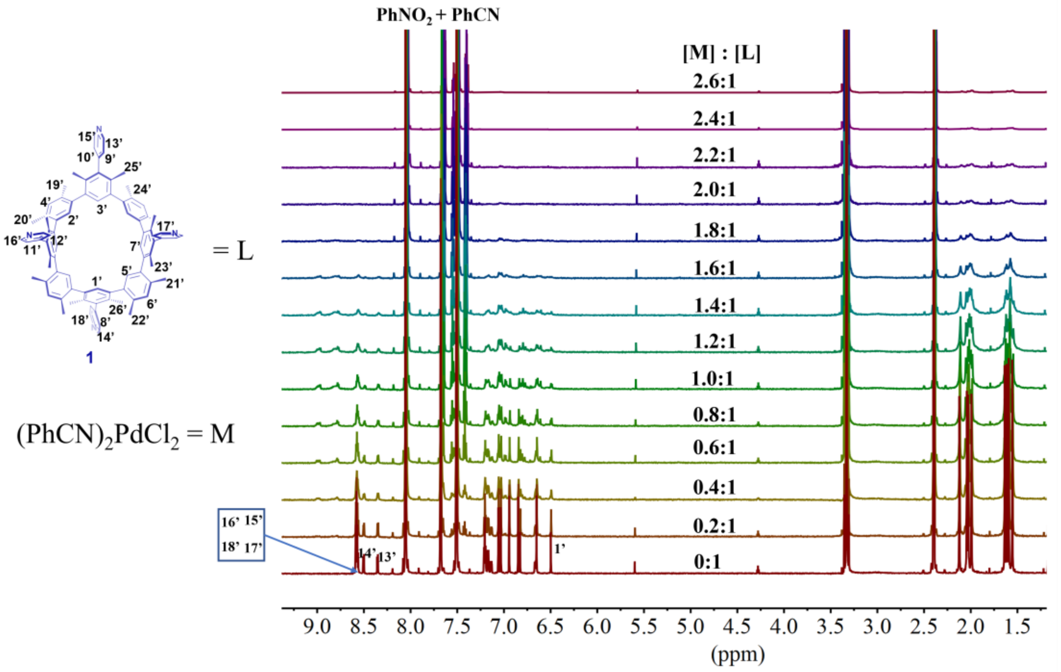

Supplementary Fig. 57 ^{1}H NMR spectroscopic titration of **1** (L; 0.100 mM) with increasing $(PhCN)_2PdCl_2$ (M; 0-2.6 molar equiv.; down to up) in $PhNO_2$-d_5/DMSO-d_6

(1/4, *v/v*) at 298K (600 MHz).

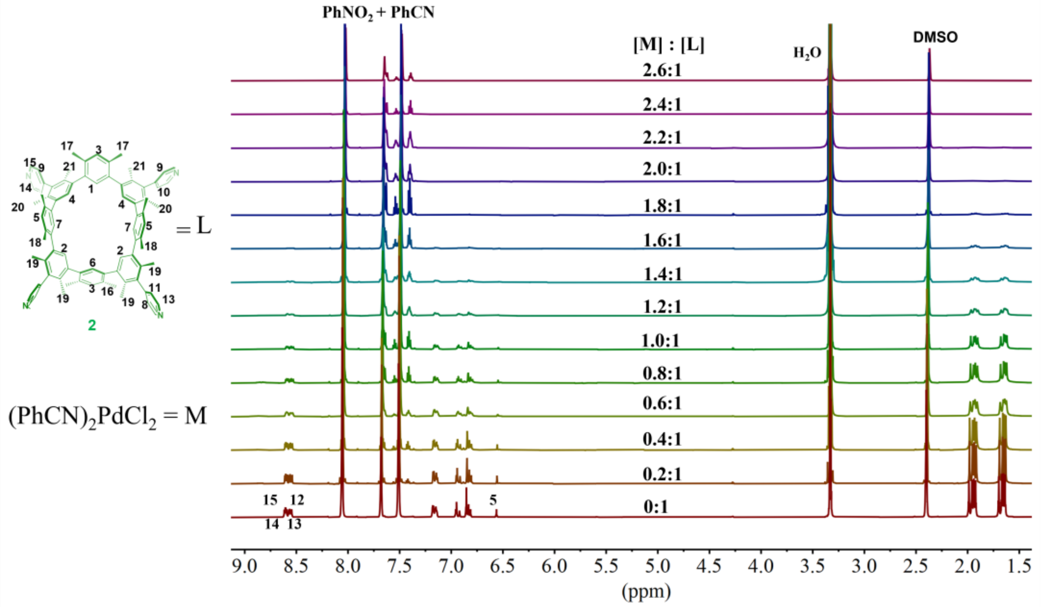

Supplementary Fig. 58 ^{1}H NMR spectroscopic titration of **2** (L; 0.10 mM) with increasing $(PhCN)_2PdCl_2$ (M, 0-2.6 molar equiv.down to up) in $PhNO_2$-*d*$_5$/DMSO-*d*$_6$ (1/4) at 298K (600 MHz).

Supplementary Note 11: Characterization of gel containing **1**, or **2** and Pd^{2+}.

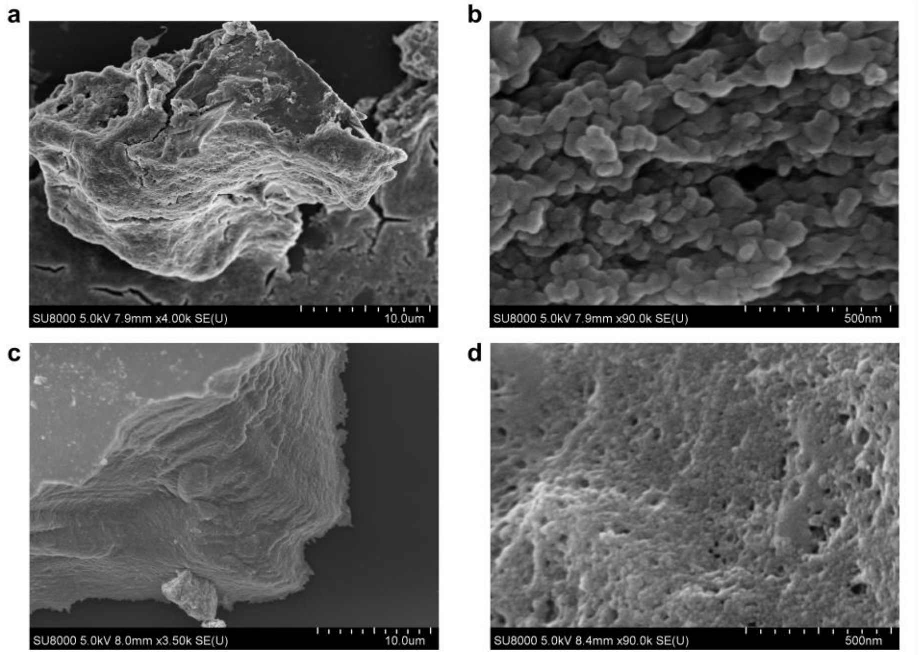

Supplementary Fig. 59 SEM micrograph of **1@2Pd** (a,b), **2@2Pd** (c,d) gels.

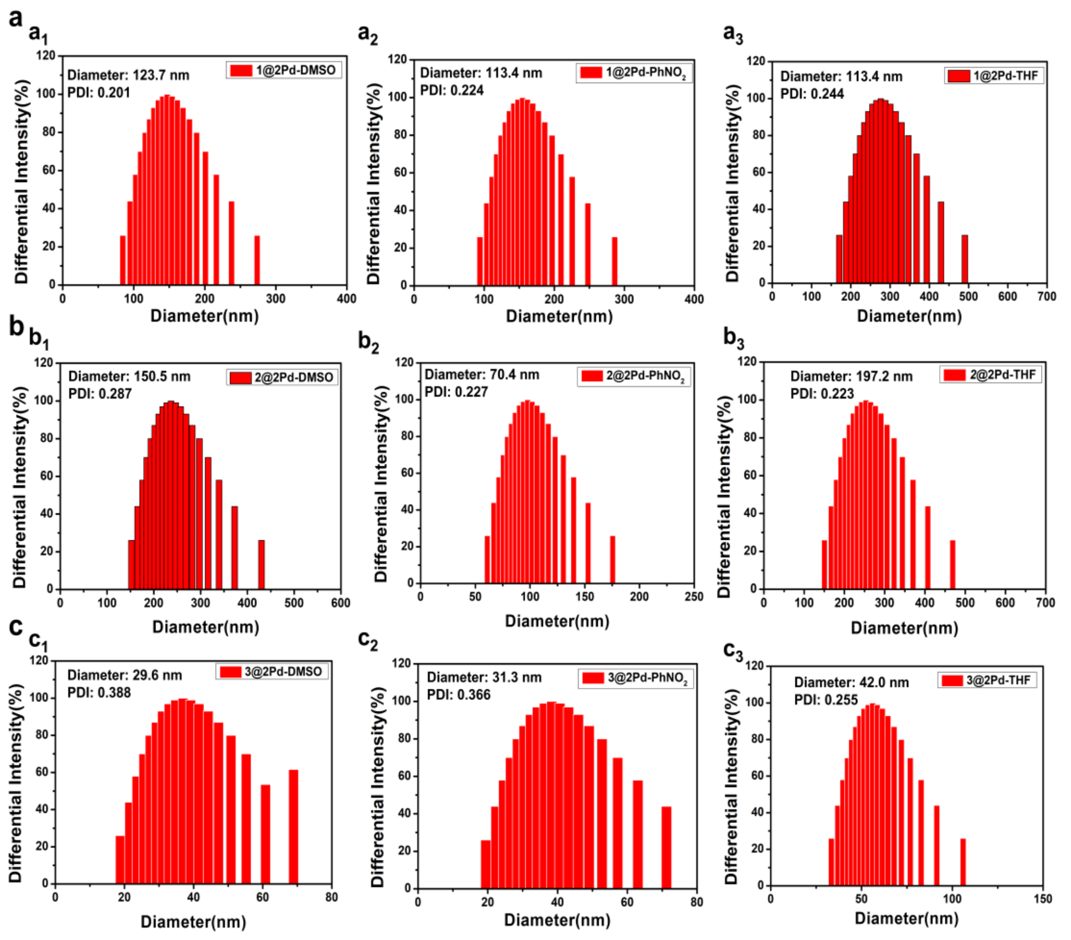

Supplementary Fig. 60 Particle size distribution of nanostructures of **1** (a), **2** (b), or **3@2Pd**. (c) dispersed in different solvents (a_1, b_1, c_1, DMSO; a_2, b_2, c_2, $PhNO_2$; a_3, b_3, c_3, THF).

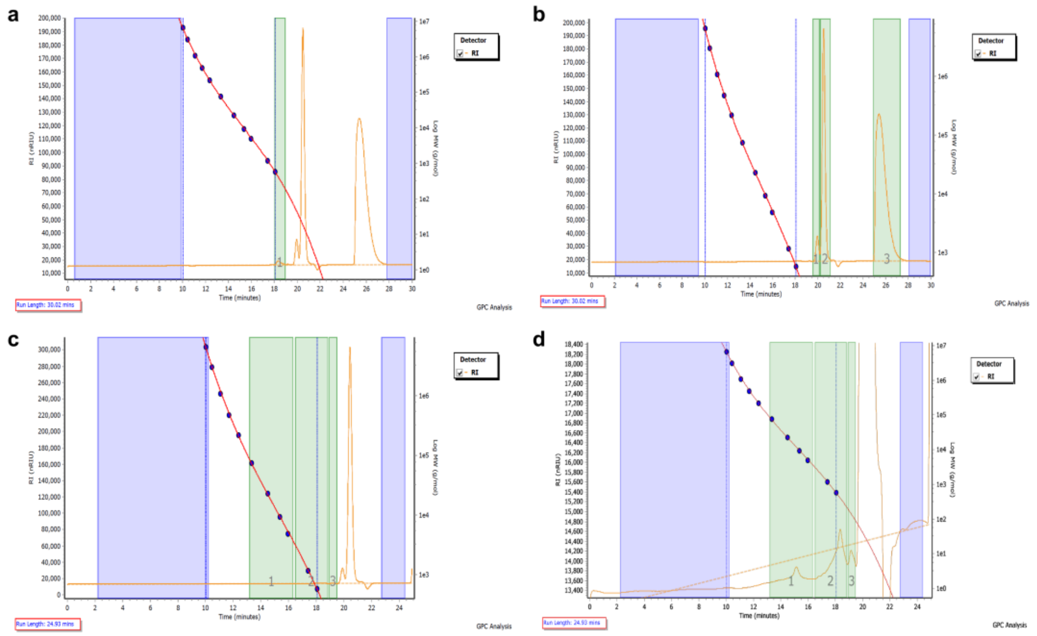

Supplementary Fig. 61 GPC spectra of **1** (a), $PhNO_2$/DMSO (1/4; *v/v*) solution(b), **1@2Pd** (c, d is the local enlarged image).

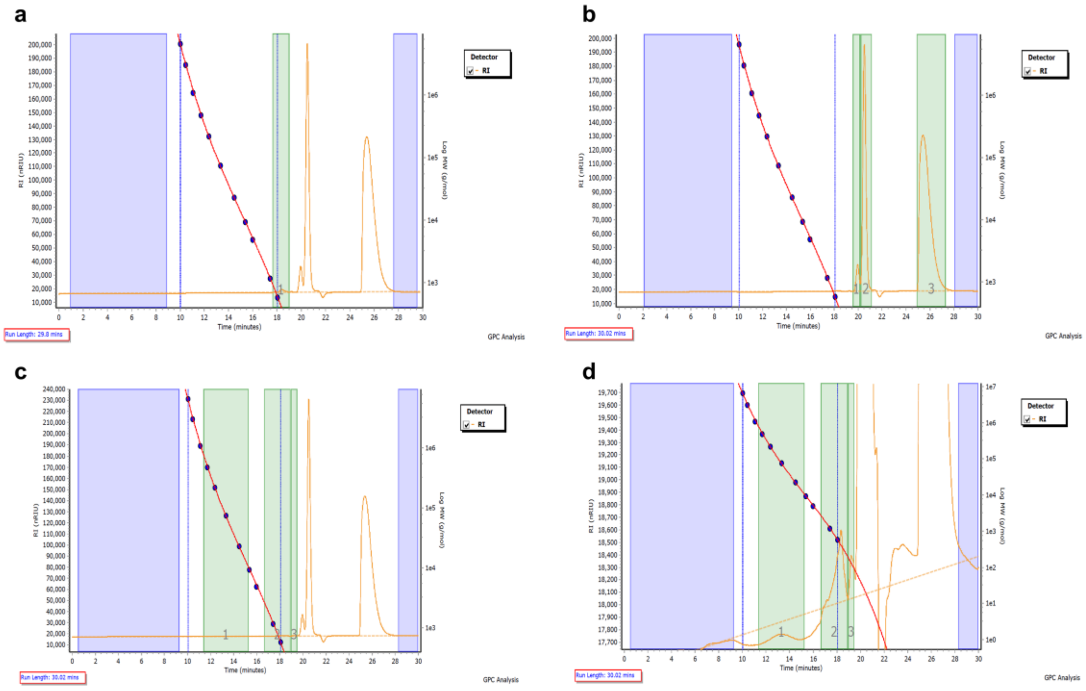

Supplementary Fig. 62 GPC spectra of **2** (a), $PhNO_2$/DMSO (1/4; *v/v*) solution(b), **2@2Pd**(c, d is the local enlarged image).

Supplementary Note 12: Optimized geometries of possible complexes among six macrocycles and twelve $(PhCN)_2PdCl_2$ and their corresponding minimized binding energies (ΔE) as calculated in vacuum.

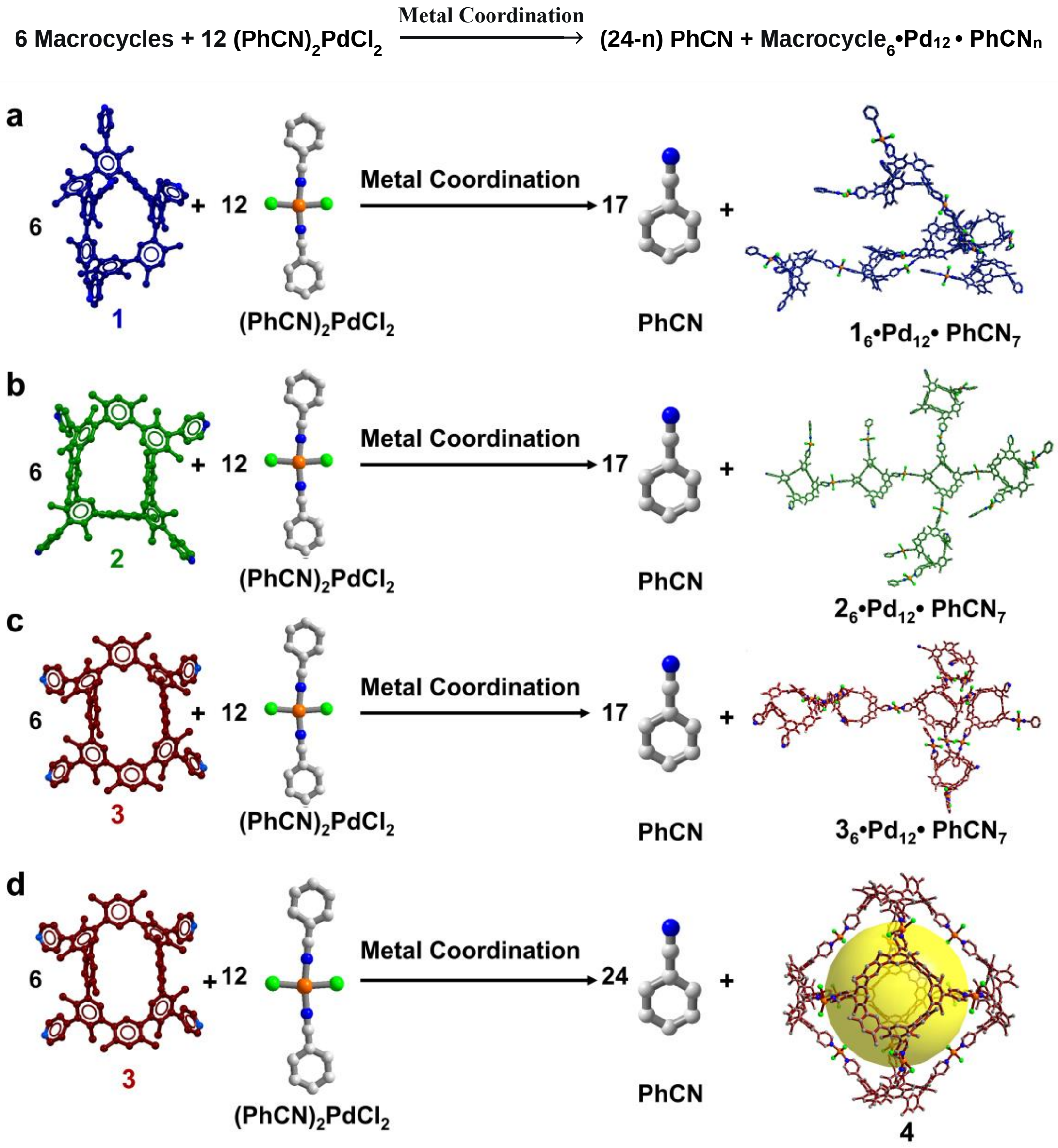

Supplementary Fig. 63 The formation energies of $\mathbf{1}_6$•Pd_{12}•$PhCN_7$(a), $\mathbf{2}_6$•Pd_{12}•$PhCN_7$ (b), $\mathbf{3}_6$•Pd_{12}•$PhCN_7$ (c), **4** (d) formed by coordination between six **1**, **2** or **3** and twelve $(PhCN)_2PdCl_2$ were calculated via molecular mechanics (MM+) using the force filed in the HyperChem 8.0 program.[5,7,8]

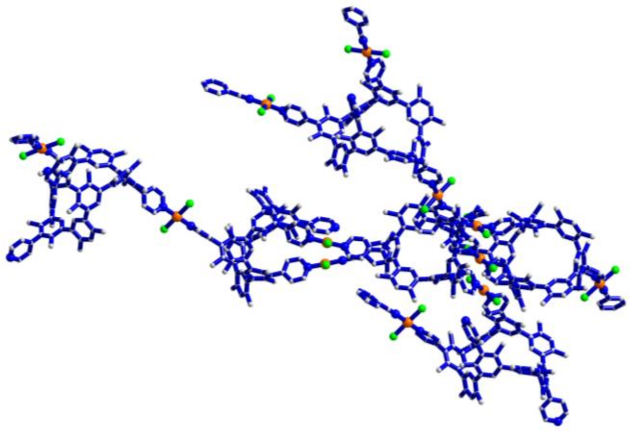

Supplementary Fig. 64 Optimized geometries of possible complexes containing six **1** and twelve Pd^{2+}.

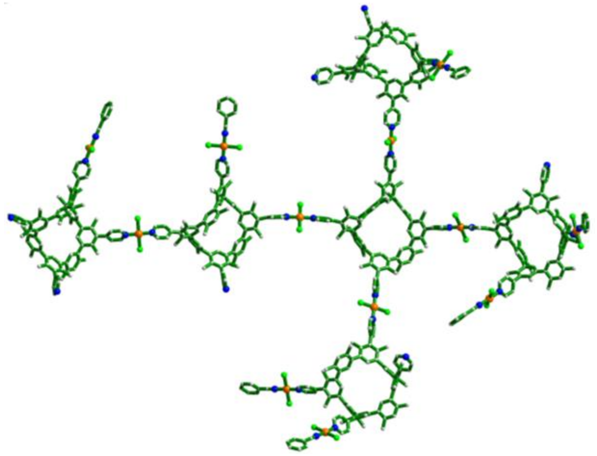

Supplementary Fig. 65 Optimized geometries of possible complexes containing six **2** and twelve Pd^{2+}.

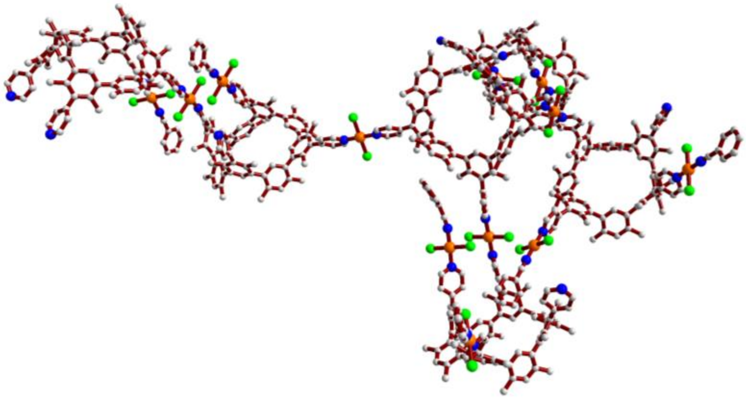

Supplementary Fig. 66 Optimized geometries of possible complexes containing six **3** and twelve Pd^{2+}.

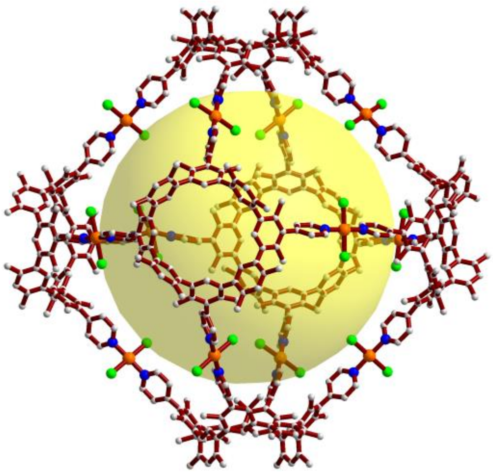

Supplementary Fig. 67 Optimized geometries of **4**.

Supplementary Table 7. The corresponding minimized binding Energies (*ΔE*) of the complexes containing six **1**, **2**, or **3** and twelve $(PhCN)_2PdCl_2$ (calculated via molecular mechanics (MM+) using the force filed in the HyperChem 8.0 program).[5,7,8]

$$\Delta E = E_{(\mathrm{macrocycle_6 \bullet Pd12 \bullet\ PhCNn})} + (24\text{-}n)\ E_{(\mathrm{PhCN})} - 12\ E_{((\mathrm{PhCN})_2\mathrm{PdCl_2})} - 6\ E_{(\mathrm{macrocycle})}$$

$(PhCN)_2PdCl_2$ = 3.14 (kJ mol^{-1})		
$(PhCN)_2PdCl_2$ = 3.14 (kJ mol^{-1})		
	**1**	$\mathbf{1}_6$•Pd_{12}•$PhCN_7$
E (kJ mol^{-1})	570.37	2034.1
Δ*E* (kJ mol^{-1})	2034.1	
	**2**	$\mathbf{2}_6$•Pd_{12}•$PhCN_7$
E (kJ mol^{-1})	514.52	2423.7
Δ*E* (kJ mol^{-1})	-643.30	
	**3**	$\mathbf{3}_6$•Pd_{12}•$PhCN_7$
E (kJ mol^{-1})	516.15	2265.9
Δ*E* (kJ mol^{-1})	-810.88	
	**3**	**4**
E (kJ mol^{-1})	516.15	3387.8
Δ*E* (kJ mol^{-1})	334.82	

Supplementary Note 13: ^{1}H-, ^{1}H DOSY NMR spectroscopic of **1**, **2**, **or 3@3Pd.**

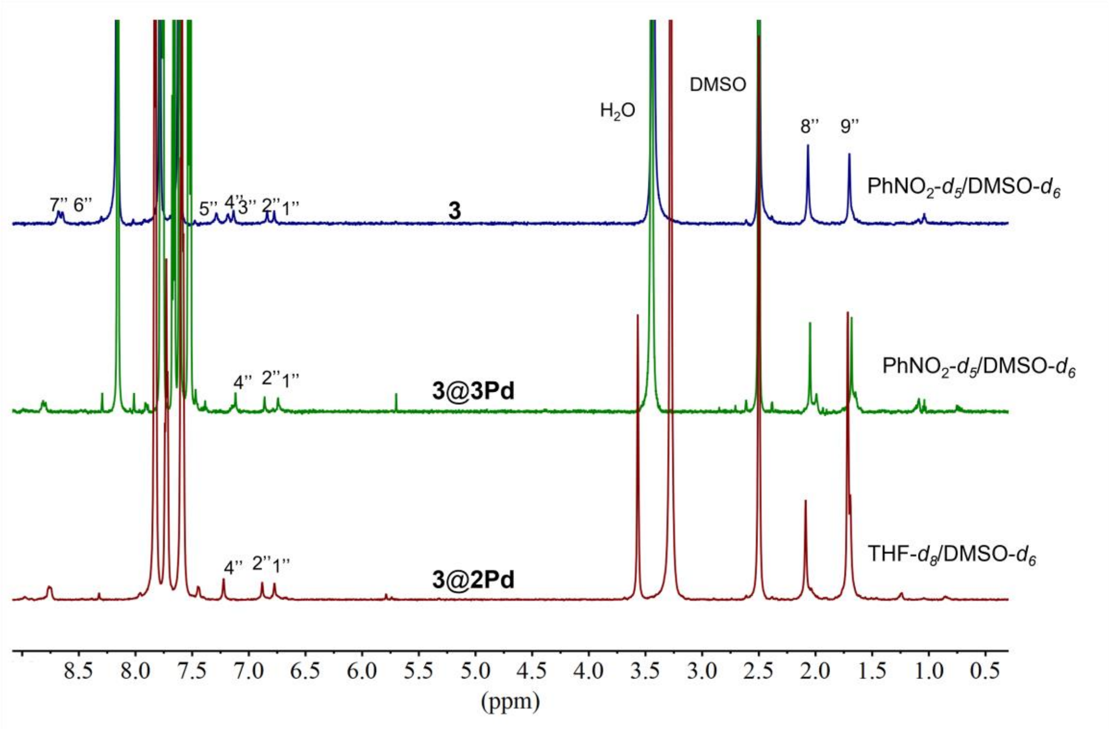

Supplementary Fig. 68 ^{1}H NMR spectra of **3**, **3@2Pd**, and **3@3Pd** in PhNO$_2$-*d*$_5$/DMSO-*d*$_6$ (1/4; *v/v*) or THF-*d*$_8$/DMSO-*d*$_6$ (1/4, *v/v*) at 298K (600 MHz).

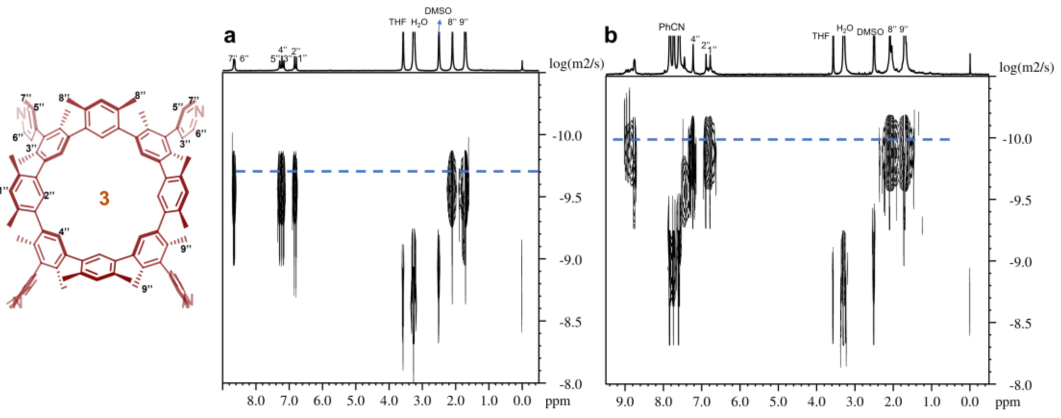

Supplementary Fig. 69 ^{1}H DOSY NMR spectra of ligand **3** (a) and **3@2Pd** (b) (600 MHz, THF-*d*$_8$/DMSO-*d*$_6$ (1/4; *v/v*), 298 K).

Stokes-Einstein equation:

$$D = \frac{\mathrm{K}T}{6\Pi \eta r_{\mathrm{H}}} \text{ (eq. 33)}$$

D is the molecular diffusion coefficient; r_H is the van der Waals radius of the molecule; K is Boltzmann's constant; T is absolute temperature; η is the viscosity of the medium. The mixed solvent (THF/DMSO) viscosity obtained by the Ubbelohde viscosimeter is $\eta_{(THF/DMSO)}$ =1.45 mPa s^{-1}. $D_{(3)}$ = 1.75 × 10^{-10} m^2 s^{-1}, $r_{H(\mathbf{3})}$ = 0.90 nm, $D_{(\mathbf{3@2Pd})}$ = 7.47 × 10^{-11} m^2 s^{-1}, $r_{H(\mathbf{3@3Pd})}$ = 2.0 nm.

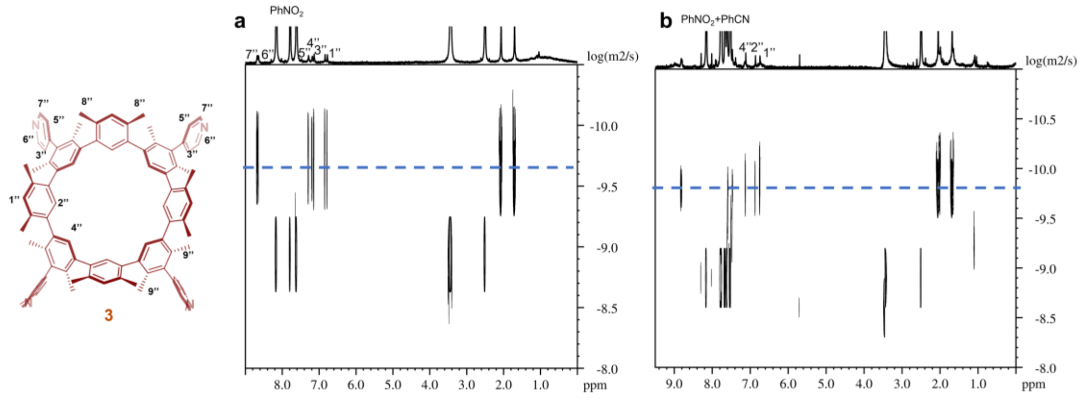

Supplementary Fig. 70 ^{1}H DOSY NMR of ligand **3** (a) and ***3*@3Pd** (b) (600 MHz, $PhNO_2$-d_5/DMSO-d_6 (1/4; *v/v*), 298K). **3 @3Pd** was heated at 338K for 100min and cooled to 298K for ^{1}H DOSY NMR test. The mixed solvent obtained by the Ubbelohde viscosimeter is $\eta_{(PhNO2/DMSO)}$=1.88 mPa s^{-1}. $D_{(3)}$ = 9.54 × 10^{-11} m^2 s^{-1}, $r_{H(3)}$ = 1.20 nm, $D_{(\mathbf{3@3Pd})}$ = 7.20 ×10^{-11} m^2 s^{-1}, $r_{H(\mathbf{3@3Pd})}$ = 1.60 nm.

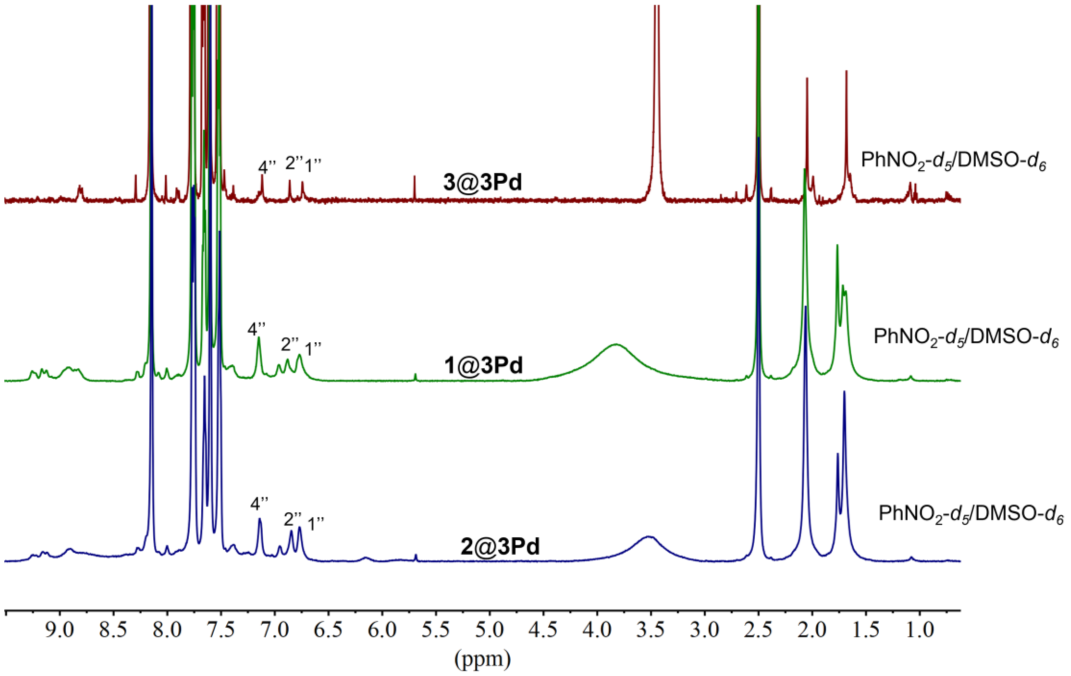

Supplementary Fig. 71 ^{1}H NMR spectra of **2@3Pd** (down), **1@3Pd** (middle), and **3@3Pd** (up) in PhNO$_2$-*d$_5$*/DMSO-*d$_6$* (1/4; *v/v*) at 298K (600 MHz). **1@3Pd** and **2@3Pd** were heated at 473K for 100 min and cooled to 298K for corresponding ^{1}H NMR test. It is suggested that the difference between **1**, **2**, and **3@3Pd** is due to the concentration difference. **3@3Pd** created precipitate in the initial assembly stage, while **1** or **2@3Pd** do not generate a large amount of precipitate during the heating promoted **4** conversion. It is supposed that **4** concentrations are higher in the latter two cases.

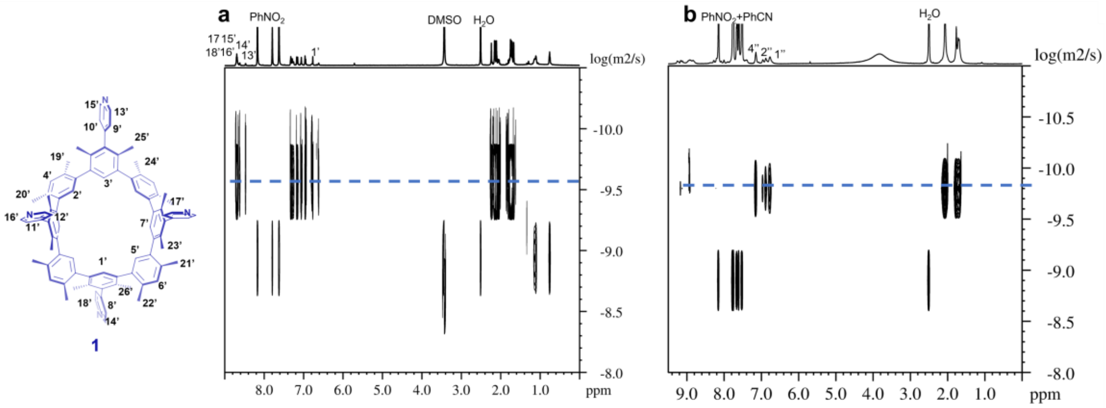

Supplementary Fig. 72 ^{1}H DOSY NMR of ligand **1** (a) and **1@3Pd** (b) (600 MHz, PhNO$_2$-*d$_5$*/DMSO-*d$_6$* (1/4; *v/v*), 298 K). **1@3Pd** was heated at 473 K for 100 min and

cooled to 298K for the ^{1}H DOSY NMR test. The mixed solvent obtained by the Ubbelohde viscosimeter is $\eta_{(PhNO2/DMSO)}$=1.88 mPa s^{-1}. $D_{(1)}$ = 1.03 × 10^{-10} m^{2} s^{-1}, $r_{H(\mathbf{1})}$ = 1.10 nm, $D_{(\mathbf{1@3Pd})}$ = 7.21 × 10^{-11} m^{2} S^{-11}, $r_{H(\mathbf{1@3Pd})}$ = 1.60 nm.

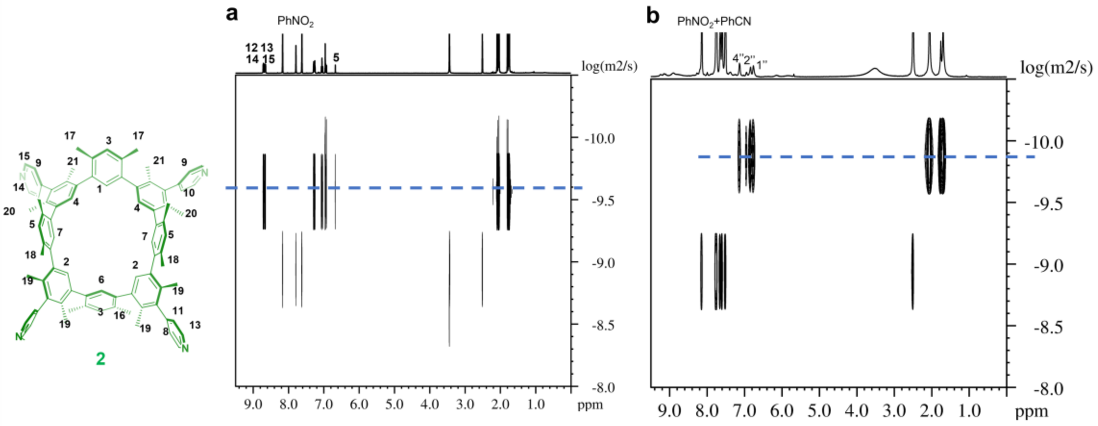

Supplementary Fig. 73 ^{1}H DOSY NMR of ligand **2** (a) and ***2*@3Pd** (b) (600 MHz, PhNO$_2$-d_5/DMSO-d_6 (1/4; v/v), 298 K). **2@3Pd** was heated at 473 K for 100 min and cooled to 298K for the ^{1}H DOSY NMR test. The mixed solvent obtained by the Ubbelohde viscosimeter is $\eta_{(PhNO2/DMSO)}$=1.88 mPa s^{-1} $D_{(2)}$ = 1.07×10^{-10} m^{2} s^{-1}, $r_{H\ (2)}$ = 1.10 nm, $D_{(\mathbf{2@3Pd})}$ = 5.96 ×10^{-11} m^{2} s^{-1}, $r_{H(\mathbf{3@3Pd})}$ = 1.90 nm.

Supplementary References

1 Sheldrick, G. M. SHELXT-integrated space-group and crystal-structure determination. *Acta Cryst.* **71**, 3-8 (2015).

2 $R_W(F^2) = \{w(|F_o|^2 - |F_c|^2)^2/w(|F_o|)^4\}^{1/2}$ where w is the weight given each reflection. R(F) = $(|F_o| - |F_c|)/ |F_o|\}$ for reflections with $F_o > 4((F_o))$. $S = [w(|F_o|^2 - |F_c|^2)^2/(n - p)]^{1/2}$, where n is the number of reflections and p is the number of refined parameters.

3 Wilson, A. J. C. International Tables for X-ray Crystallography. Vol. C, Tables 4.2.6.8 and 6.1.1.4 (Kluwer Academic Press, 1992).

4 Sheldrick, G. M. SHELXTL/PC (Version 5.03) (Siemens Analytical X-ray Instruments, Inc., Wisconsin, 1994).

5 *HyperChem*, version 8.0; Hypercube: Gainesville, FL, 2002.

6 Stewart, J. J. P. *Stewart Computational Chemistry-MOPAC*; Colorado Springs, CO, 2016.

7 Viossat, B.; Dung, N.-H.; Robert, F. Structure du trans-Dichlorobis(pyridine)palladium(II). *Acta Cryst. C49*, 84-85 (1993).

8 Olmstead, M. M.; Wei, P.-p.; Ginwalla, A. S.; Balch, A. L. Bis(benzonitrile)palladium(II) Dihalides: Structures and Cocrystallization of the Cubic Cluster Pd_6Cl_{12} with (E)-Stilbene and with Bis(benzonitrile)palladium(II) Dichloride. *Inorg. Chem. 39*, 4555-4559 (2000).

checkCIF/PLATON report

Structure factors have been supplied for datablock(s) I

THIS REPORT IS FOR GUIDANCE ONLY. IF USED AS PART OF A REVIEW PROCEDURE FOR PUBLICATION, IT SHOULD NOT REPLACE THE EXPERTISE OF AN EXPERIENCED CRYSTALLOGRAPHIC REFEREE.

No syntax errors found. CIF dictionary Interpreting this report

Datablock: 1•2.25•CH_2Cl_2

Bond precision:	C-C = 0.0092 A		Wavelength=1.54184
Cell:	a=13.2724(8)	b=27.336(3)	c=20.0597(15)
	alpha=90	beta=90	gamma=90
Temperature:	170 K		

	Calculated	Reported
Volume	7278.0(11)	7278.1(10)
Space group	P n m a	P n m a
Hall group	-P 2ac 2n	-P 2ac 2n
Moiety formula	4(C84 H76 N4), 6(C0.50 H Cl), 6(C H2 Cl2)	?
Sum formula	C345 H322 Cl18 N16	C86.25 H80.50 Cl4.50 N4
Mr	5330.29	1332.57
Dx,g cm-3	1.216	1.216
Z	1	4
Mu (mm-1)	2.010	2.010
F000	2810.0	2810.0
F000'	2822.97	
h,k,lmax	15,31,23	15,31,23
Nref	5947	5925
Tmin,Tmax	0.953,0.961	0.814,1.000
Tmin'	0.904	

Correction method= # Reported T Limits: Tmin=0.814 Tmax=1.000
AbsCorr = MULTI-SCAN

Data completeness= 0.996 Theta(max)= 62.492

R(reflections)= 0.0986(3086) wR2(reflections)= 0.2440(5925)

S = 1.012 Npar= 471

The following ALERTS were generated. Each ALERT has the format

test-name_ALERT_alert-type_alert-level.

Click on the hyperlinks for more details of the test.

Alert level C

```
ABSTY02_ALERT_1_C  An _exptl_absorpt_correction_type has been given without
            a literature citation. This should be contained in the
            _exptl_absorpt_process_details field.
            Absorption correction given as multi-scan
THETM01_ALERT_3_C  The value of sine(theta_max)/wavelength is less than 0.590
            Calculated sin(theta_max)/wavelength =    0.5753
PLAT023_ALERT_3_C Resolution (too) Low [sin(theta)/Lambda < 0.6]..       0.58 Ang-1
PLAT048_ALERT_1_C MoietyFormula Not Given (or Incomplete) ........     Please Check
PLAT218_ALERT_3_C Constrained U(ij) Components(s) for C51A       .          2 Check
PLAT220_ALERT_2_C NonSolvent   Resd 1  C   Ueq(max)/Ueq(min) Range        3.4 Ratio
PLAT241_ALERT_2_C High   'MainMol' Ueq as Compared to Neighbors of        C45 Check
PLAT241_ALERT_2_C High   'MainMol' Ueq as Compared to Neighbors of        C46 Check
PLAT242_ALERT_2_C Low    'MainMol' Ueq as Compared to Neighbors of         N3 Check
PLAT242_ALERT_2_C Low    'MainMol' Ueq as Compared to Neighbors of        C47 Check
PLAT260_ALERT_2_C Large Average Ueq of Residue Including       Cl1      0.153 Check
PLAT260_ALERT_2_C Large Average Ueq of Residue Including       Cl3      0.121 Check
PLAT260_ALERT_2_C Large Average Ueq of Residue Including      Cl1A      0.188 Check
PLAT260_ALERT_2_C Large Average Ueq of Residue Including      Cl3A      0.195 Check
PLAT329_ALERT_4_C Carbon Atom Hybridisation Unclear for ..........        C50 Check
PLAT329_ALERT_4_C Carbon Atom Hybridisation Unclear for ..........       C50A Check
PLAT340_ALERT_3_C Low Bond Precision on  C-C Bonds ...............    0.00919 Ang.
PLAT906_ALERT_3_C Large K Value in the Analysis of Variance ......     20.590 Check
PLAT906_ALERT_3_C Large K Value in the Analysis of Variance ......      3.640 Check
PLAT911_ALERT_3_C Missing FCF Refl Between Thmin & STh/L=    0.575         23 Report
```

Alert level G

```
PLAT002_ALERT_2_G Number of Distance or Angle Restraints on AtSite         12 Note
PLAT003_ALERT_2_G Number of Uiso or Uij Restrained non-H Atoms ...         24 Report
PLAT045_ALERT_1_G Calculated and Reported Z Differ by a Factor ...         0. Check
PLAT083_ALERT_2_G SHELXL Second Parameter in WGHT  Unusually Large      25.00 Why ?
PLAT171_ALERT_4_G The CIF-Embedded .res File Contains EADP Records          3 Report
PLAT172_ALERT_4_G The CIF-Embedded .res File Contains DFIX Records          4 Report
PLAT177_ALERT_4_G The CIF-Embedded .res File Contains DELU Records          1 Report
PLAT178_ALERT_4_G The CIF-Embedded .res File Contains SIMU Records          1 Report
PLAT186_ALERT_4_G The CIF-Embedded .res File Contains ISOR Records          1 Report
PLAT300_ALERT_4_G Atom Site Occupancy of N2         Constrained at        0.5 Check
PLAT300_ALERT_4_G Atom Site Occupancy of N2A        Constrained at        0.5 Check
PLAT300_ALERT_4_G Atom Site Occupancy of C40        Constrained at        0.5 Check
PLAT300_ALERT_4_G Atom Site Occupancy of C40A       Constrained at        0.5 Check
PLAT300_ALERT_4_G Atom Site Occupancy of C41        Constrained at        0.5 Check
PLAT300_ALERT_4_G Atom Site Occupancy of C41A       Constrained at        0.5 Check
PLAT300_ALERT_4_G Atom Site Occupancy of C42        Constrained at        0.5 Check
PLAT300_ALERT_4_G Atom Site Occupancy of C42A       Constrained at        0.5 Check
PLAT300_ALERT_4_G Atom Site Occupancy of C43        Constrained at        0.5 Check
PLAT300_ALERT_4_G Atom Site Occupancy of C43A       Constrained at        0.5 Check
PLAT300_ALERT_4_G Atom Site Occupancy of C44        Constrained at        0.5 Check
PLAT300_ALERT_4_G Atom Site Occupancy of C44A       Constrained at        0.5 Check
PLAT300_ALERT_4_G Atom Site Occupancy of H40A       Constrained at        0.5 Check
PLAT300_ALERT_4_G Atom Site Occupancy of H40B       Constrained at        0.5 Check
```

```
PLAT300_ALERT_4_G Atom Site Occupancy of H41A       Constrained at        0.5 Check
PLAT300_ALERT_4_G Atom Site Occupancy of H41B       Constrained at        0.5 Check
PLAT300_ALERT_4_G Atom Site Occupancy of H43A       Constrained at        0.5 Check
PLAT300_ALERT_4_G Atom Site Occupancy of H43B       Constrained at        0.5 Check
PLAT300_ALERT_4_G Atom Site Occupancy of H44A       Constrained at        0.5 Check
PLAT300_ALERT_4_G Atom Site Occupancy of H44B       Constrained at        0.5 Check
PLAT300_ALERT_4_G Atom Site Occupancy of Cl1        Constrained at        0.5 Check
PLAT300_ALERT_4_G Atom Site Occupancy of Cl2        Constrained at        0.5 Check
PLAT300_ALERT_4_G Atom Site Occupancy of C50        Constrained at        0.5 Check
PLAT300_ALERT_4_G Atom Site Occupancy of H50A       Constrained at       0.25 Check
PLAT300_ALERT_4_G Atom Site Occupancy of H50B       Constrained at       0.25 Check
PLAT300_ALERT_4_G Atom Site Occupancy of Cl3        Constrained at        0.5 Check
PLAT300_ALERT_4_G Atom Site Occupancy of Cl4        Constrained at        0.5 Check
PLAT300_ALERT_4_G Atom Site Occupancy of C51        Constrained at        0.5 Check
PLAT300_ALERT_4_G Atom Site Occupancy of H51A       Constrained at        0.5 Check
PLAT300_ALERT_4_G Atom Site Occupancy of H51B       Constrained at        0.5 Check
PLAT300_ALERT_4_G Atom Site Occupancy of Cl1A       Constrained at       0.25 Check
PLAT300_ALERT_4_G Atom Site Occupancy of Cl2A       Constrained at       0.25 Check
PLAT300_ALERT_4_G Atom Site Occupancy of C50A       Constrained at       0.25 Check
PLAT300_ALERT_4_G Atom Site Occupancy of H50C       Constrained at      0.125 Check
PLAT300_ALERT_4_G Atom Site Occupancy of H50D       Constrained at      0.125 Check
PLAT300_ALERT_4_G Atom Site Occupancy of Cl3A       Constrained at       0.25 Check
PLAT300_ALERT_4_G Atom Site Occupancy of Cl4A       Constrained at       0.25 Check
PLAT300_ALERT_4_G Atom Site Occupancy of C51A       Constrained at       0.25 Check
PLAT300_ALERT_4_G Atom Site Occupancy of H51C       Constrained at       0.25 Check
PLAT300_ALERT_4_G Atom Site Occupancy of H51D       Constrained at       0.25 Check
PLAT301_ALERT_3_G Main Residue  Disorder .............(Resd  1  )        14% Note
PLAT302_ALERT_4_G Anion/Solvent/Minor-Residue Disorder (Resd  2  )       100% Note
PLAT302_ALERT_4_G Anion/Solvent/Minor-Residue Disorder (Resd  3  )       100% Note
PLAT302_ALERT_4_G Anion/Solvent/Minor-Residue Disorder (Resd  4  )       100% Note
PLAT302_ALERT_4_G Anion/Solvent/Minor-Residue Disorder (Resd  5  )       100% Note
PLAT304_ALERT_4_G Non-Integer Number of Atoms in ..... (Resd  2  )       1.25 Check
PLAT304_ALERT_4_G Non-Integer Number of Atoms in ..... (Resd  3  )       2.50 Check
PLAT304_ALERT_4_G Non-Integer Number of Atoms in ..... (Resd  4  )       0.62 Check
PLAT304_ALERT_4_G Non-Integer Number of Atoms in ..... (Resd  5  )       1.25 Check
PLAT380_ALERT_4_G Incorrectly? Oriented X(sp2)-Methyl Moiety .....         C5 Check
PLAT380_ALERT_4_G Incorrectly? Oriented X(sp2)-Methyl Moiety .....        C12 Check
PLAT380_ALERT_4_G Incorrectly? Oriented X(sp2)-Methyl Moiety .....        C13 Check
PLAT380_ALERT_4_G Incorrectly? Oriented X(sp2)-Methyl Moiety .....        C20 Check
PLAT380_ALERT_4_G Incorrectly? Oriented X(sp2)-Methyl Moiety .....        C21 Check
PLAT380_ALERT_4_G Incorrectly? Oriented X(sp2)-Methyl Moiety .....        C28 Check
PLAT380_ALERT_4_G Incorrectly? Oriented X(sp2)-Methyl Moiety .....        C29 Check
PLAT380_ALERT_4_G Incorrectly? Oriented X(sp2)-Methyl Moiety .....        C34 Check
PLAT413_ALERT_2_G Short Inter XH3 .. XHn     H29A     ..H50C     .       2.01 Ang.
                                      1/2+x,1/2-y,3/2-z  =      4_556 Check
PLAT413_ALERT_2_G Short Inter XH3 .. XHn     H29A     ..H50D     .       2.01 Ang.
                                          1/2+x,y,3/2-z  =      6_657 Check
PLAT720_ALERT_4_G Number of Unusual/Non-Standard Labels .........          1 Note
PLAT779_ALERT_4_G Suspect or Irrelevant (Bond) Angle(s) in CIF ...      32.00 Deg.
              H50B -C50  -H50C    1_555   1_555   7_565 ......     #  201 Check
PLAT779_ALERT_4_G Suspect or Irrelevant (Bond) Angle(s) in CIF ...      32.00 Deg.
              H50A -C50  -H50D    1_555   1_555   7_565 ......     #  204 Check
PLAT790_ALERT_4_G Centre of Gravity not Within Unit Cell: Resd.  #          3 Note
              C H2 Cl2
PLAT790_ALERT_4_G Centre of Gravity not Within Unit Cell: Resd.  #          5 Note
              C H2 Cl2
PLAT860_ALERT_3_G Number of Least-Squares Restraints ............        210 Note
```

```
PLAT883_ALERT_1_G No Info/Value for _atom_sites_solution_primary .     Please Do !
PLAT933_ALERT_2_G Number of HKL-OMIT Records in Embedded .res File         20 Note
PLAT941_ALERT_3_G Average HKL Measurement Multiplicity ..........         3.9 Low
PLAT960_ALERT_3_G Number of Intensities with I  <   - 2*sig(I) ...          3 Check
PLAT965_ALERT_2_G The SHELXL WEIGHT Optimisation has not Converged     Please Check
PLAT967_ALERT_5_G Note: Two-Theta Cutoff Value in Embedded .res ..      125.0 Degree
PLAT978_ALERT_2_G Number C-C Bonds with Positive Residual Density.          1 Info
```

```
   0 ALERT level A = Most likely a serious problem - resolve or explain
   0 ALERT level B = A potentially serious problem, consider carefully
  20 ALERT level C = Check. Ensure it is not caused by an omission or oversight
  81 ALERT level G = General information/check it is not something unexpected

   4 ALERT type 1 CIF construction/syntax error, inconsistent or missing data
  17 ALERT type 2 Indicator that the structure model may be wrong or deficient
  11 ALERT type 3 Indicator that the structure quality may be low
  68 ALERT type 4 Improvement, methodology, query or suggestion
   1 ALERT type 5 Informative message, check
```

Publication of your CIF

You should attempt to resolve as many as possible of the alerts in all categories. Often the minor alerts point to easily fixed oversights, errors and omissions in your CIF or refinement strategy, so attention to these fine details can be worthwhile. In order to resolve some of the more serious problems it may be necessary to carry out additional measurements or structure refinements. However, the nature of your study may justify the reported deviations from journal submission requirements and the more serious of these should be commented upon in the discussion or experimental section of a paper or in the "special_details" fields of the CIF. *checkCIF* was carefully designed to identify outliers and unusual parameters, but every test has its limitations and alerts that are not important in a particular case may appear. Conversely, the absence of alerts does not guarantee there are no aspects of the results needing attention. It is up to the individual to critically assess their own results and, if necessary, seek expert advice.

If you wish to submit your CIF for publication in Acta Crystallographica Section C or E, you should upload your CIF via the web. If you wish to submit your CIF for publication in IUCrData you should upload your CIF via the web. If your CIF is to form part of a submission to another IUCr journal, you will be asked, either during electronic submission or by the Co-editor handling your paper, to upload your CIF via our web site.

PLATON version of 19/02/2022; check.def file version of 19/01/2022

Datablock I - ellipsoid plot

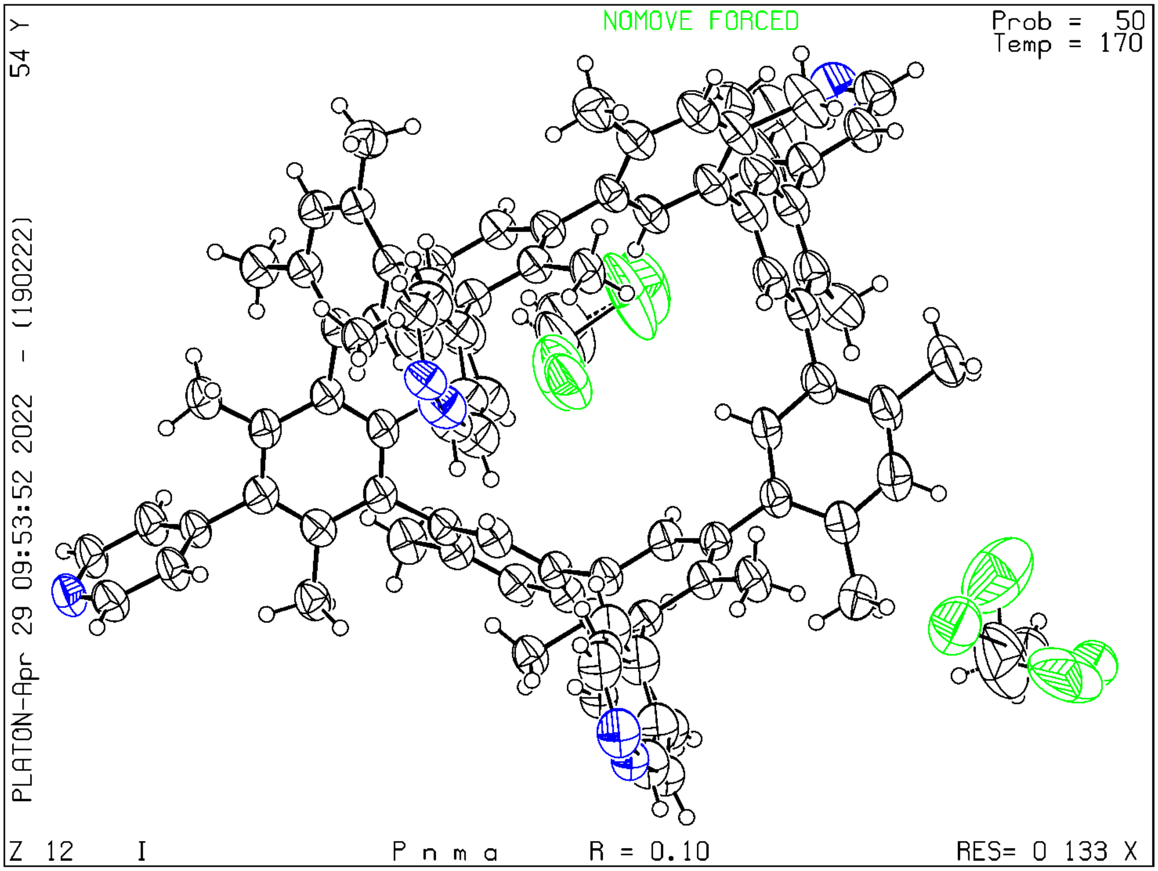

checkCIF/PLATON report

Structure factors have been supplied for datablock(s) I

THIS REPORT IS FOR GUIDANCE ONLY. IF USED AS PART OF A REVIEW PROCEDURE FOR PUBLICATION, IT SHOULD NOT REPLACE THE EXPERTISE OF AN EXPERIENCED CRYSTALLOGRAPHIC REFEREE.

No syntax errors found. CIF dictionary Interpreting this report

Datablock: 2•CH_2Cl_2•0.75H_2O

Bond precision: C-C = 0.0072 A Wavelength=1.54184

Cell:	a=16.7411(9)	b=16.0298(8)	c=26.5504(12)
	alpha=90	beta=97.788(4)	gamma=90
Temperature:	170 K		

	Calculated	Reported
Volume	7059.3(6)	7059.3(6)
Space group	P 21/c	P 21/c
Hall group	-P 2ybc	-P 2ybc
Moiety formula	4(C84 H76 N4), 4(C H2 Cl2), 3(O)	?
Sum formula	C340 H312 Cl8 N16 O3	C85 H79.50 Cl2 N4 O0.75
Mr	4953.68	1239.92
Dx,g cm-3	1.165	1.167
Z	1	4
Mu (mm-1)	1.195	1.195
F000	2624.0	2630.0
F000’	2633.35	
h,k,lmax	19,18,30	19,18,30
Nref	11276	11267
Tmin,Tmax	0.806,0.887	0.187,1.000
Tmin’	0.806	

Correction method= # Reported T Limits: Tmin=0.187 Tmax=1.000
AbsCorr = MULTI-SCAN

Data completeness= 0.999 Theta(max)= 62.497

R(reflections)= 0.0866(6210) wR2(reflections)= 0.2867(11267)

S = 1.001 Npar= 865

```
The following ALERTS were generated. Each ALERT has the format
       test-name_ALERT_alert-type_alert-level.
Click on the hyperlinks for more details of the test.
```

Alert level C

```
ABSTY02_ALERT_1_C  An _exptl_absorpt_correction_type has been given without
            a literature citation. This should be contained in the
            _exptl_absorpt_process_details field.
            Absorption correction given as multi-scan
DIFMX02_ALERT_1_C  The maximum difference density is > 0.1*ZMAX*0.75
            The relevant atom site should be identified.
THETM01_ALERT_3_C  The value of sine(theta_max)/wavelength is less than 0.590
            Calculated sin(theta_max)/wavelength =    0.5753
PLAT018_ALERT_1_C _diffrn_measured_fraction_theta_max .NE. *_full           ! Check
PLAT041_ALERT_1_C Calc. and Reported SumFormula    Strings  Differ     Please Check
PLAT043_ALERT_1_C Calculated and Reported Mol. Weight Differ by ..       6.00 Check
PLAT048_ALERT_1_C MoietyFormula Not Given (or Incomplete) ........     Please Check
PLAT068_ALERT_1_C Reported F000 Differs from Calcd (or Missing)...     Please Check
PLAT084_ALERT_3_C High wR2 Value (i.e. > 0.25) ...................       0.29 Report
PLAT094_ALERT_2_C Ratio of Maximum / Minimum Residual Density ....       3.26 Report
PLAT097_ALERT_2_C Large Reported Max.  (Positive) Residual Density       1.48 eA-3
PLAT234_ALERT_4_C Large Hirshfeld Difference N3       --C75      .       0.16 Ang.
PLAT234_ALERT_4_C Large Hirshfeld Difference C75      --C76      .       0.17 Ang.
PLAT241_ALERT_2_C High   'MainMol' Ueq as Compared to Neighbors of        C65 Check
PLAT241_ALERT_2_C High   'MainMol' Ueq as Compared to Neighbors of        C84 Check
PLAT260_ALERT_2_C Large Average Ueq of Residue Including       Cl1      0.150 Check
PLAT260_ALERT_2_C Large Average Ueq of Residue Including      Cl1A      0.165 Check
PLAT260_ALERT_2_C Large Average Ueq of Residue Including       O1W      0.144 Check
PLAT260_ALERT_2_C Large Average Ueq of Residue Including       O2W      0.157 Check
PLAT340_ALERT_3_C Low Bond Precision on  C-C Bonds ..............    0.00721 Ang.
PLAT413_ALERT_2_C Short Inter XH3 .. XHn     H8C      ..H32C     .       2.02 Ang.
                                               1-x,1-y,1-z  =      3_666 Check
PLAT413_ALERT_2_C Short Inter XH3 .. XHn     H56A     ..H79A     .       2.09 Ang.
                                             x,3/2-y,1/2+z  =      4_576 Check
PLAT601_ALERT_2_C Unit Cell Contains Solvent Accessible VOIDS of .         35 Ang**3
PLAT906_ALERT_3_C Large K Value in the Analysis of Variance ......      5.230 Check
PLAT911_ALERT_3_C Missing FCF Refl Between Thmin & STh/L=    0.575          9 Report
PLAT934_ALERT_3_C Number of (Iobs-Icalc)/Sigma(W) > 10 Outliers ..          1 Check
```

Alert level G

```
FORMU01_ALERT_2_G  There is a discrepancy between the atom counts in the
            _chemical_formula_sum and the formula from the _atom_site* data.
            Atom count from _chemical_formula_sum:C85 H79.5 Cl2 N4 O0.75
            Atom count from the _atom_site data:  C85 H78 Cl2 N4 O0.75
CELLZ01_ALERT_1_G Difference between formula and atom_site contents detected.
CELLZ01_ALERT_1_G WARNING: H atoms missing from atom site list. Is this intentional?
           From the CIF: _cell_formula_units_Z    4
           From the CIF: _chemical_formula_sum  C85 H79.50 Cl2 N4 O0.75
           TEST: Compare cell contents of formula and atom_site data

           atom    Z*formula  cif sites diff
           C         340.00    340.00    0.00
           H         318.00    312.00    6.00
           Cl          8.00      8.00    0.00
           N          16.00     16.00    0.00
           O           3.00      3.00    0.00
PLAT002_ALERT_2_G Number of Distance or Angle Restraints on AtSite          6 Note
PLAT003_ALERT_2_G Number of Uiso or Uij Restrained non-H Atoms ...          8 Report
PLAT045_ALERT_1_G Calculated and Reported Z Differ by a Factor ...       0.25 Check
```

```
PLAT172_ALERT_4_G The CIF-Embedded .res File Contains DFIX Records          2 Report
PLAT177_ALERT_4_G The CIF-Embedded .res File Contains DELU Records          1 Report
PLAT178_ALERT_4_G The CIF-Embedded .res File Contains SIMU Records          1 Report
PLAT186_ALERT_4_G The CIF-Embedded .res File Contains ISOR Records          1 Report
PLAT300_ALERT_4_G Atom Site Occupancy of Cl1        Constrained at       0.67 Check
PLAT300_ALERT_4_G Atom Site Occupancy of Cl2        Constrained at       0.67 Check
PLAT300_ALERT_4_G Atom Site Occupancy of C85        Constrained at       0.67 Check
PLAT300_ALERT_4_G Atom Site Occupancy of H85A       Constrained at       0.67 Check
PLAT300_ALERT_4_G Atom Site Occupancy of H85B       Constrained at       0.67 Check
PLAT300_ALERT_4_G Atom Site Occupancy of Cl1A       Constrained at       0.33 Check
PLAT300_ALERT_4_G Atom Site Occupancy of Cl2A       Constrained at       0.33 Check
PLAT300_ALERT_4_G Atom Site Occupancy of C85A       Constrained at       0.33 Check
PLAT300_ALERT_4_G Atom Site Occupancy of H85C       Constrained at       0.33 Check
PLAT300_ALERT_4_G Atom Site Occupancy of H85D       Constrained at       0.33 Check
PLAT300_ALERT_4_G Atom Site Occupancy of O1W        Constrained at        0.5 Check
PLAT300_ALERT_4_G Atom Site Occupancy of O2W        Constrained at       0.25 Check
PLAT302_ALERT_4_G Anion/Solvent/Minor-Residue Disorder (Resd  2  )       100% Note
PLAT302_ALERT_4_G Anion/Solvent/Minor-Residue Disorder (Resd  3  )       100% Note
PLAT302_ALERT_4_G Anion/Solvent/Minor-Residue Disorder (Resd  4  )       100% Note
PLAT302_ALERT_4_G Anion/Solvent/Minor-Residue Disorder (Resd  5  )       100% Note
PLAT304_ALERT_4_G Non-Integer Number of Atoms in ..... (Resd  2  )       3.35 Check
PLAT304_ALERT_4_G Non-Integer Number of Atoms in ..... (Resd  3  )       1.65 Check
PLAT304_ALERT_4_G Non-Integer Number of Atoms in ..... (Resd  4  )       0.50 Check
PLAT304_ALERT_4_G Non-Integer Number of Atoms in ..... (Resd  5  )       0.25 Check
PLAT311_ALERT_2_G Isolated Disordered Oxygen Atom (No H’s ?) .....        O1W Check
PLAT311_ALERT_2_G Isolated Disordered Oxygen Atom (No H’s ?) .....        O2W Check
PLAT380_ALERT_4_G Incorrectly? Oriented X(sp2)-Methyl Moiety .....         C7 Check
PLAT380_ALERT_4_G Incorrectly? Oriented X(sp2)-Methyl Moiety .....         C8 Check
PLAT380_ALERT_4_G Incorrectly? Oriented X(sp2)-Methyl Moiety .....        C15 Check
PLAT380_ALERT_4_G Incorrectly? Oriented X(sp2)-Methyl Moiety .....        C16 Check
PLAT380_ALERT_4_G Incorrectly? Oriented X(sp2)-Methyl Moiety .....        C23 Check
PLAT380_ALERT_4_G Incorrectly? Oriented X(sp2)-Methyl Moiety .....        C24 Check
PLAT380_ALERT_4_G Incorrectly? Oriented X(sp2)-Methyl Moiety .....        C31 Check
PLAT380_ALERT_4_G Incorrectly? Oriented X(sp2)-Methyl Moiety .....        C32 Check
PLAT380_ALERT_4_G Incorrectly? Oriented X(sp2)-Methyl Moiety .....        C39 Check
PLAT380_ALERT_4_G Incorrectly? Oriented X(sp2)-Methyl Moiety .....        C40 Check
PLAT380_ALERT_4_G Incorrectly? Oriented X(sp2)-Methyl Moiety .....        C47 Check
PLAT380_ALERT_4_G Incorrectly? Oriented X(sp2)-Methyl Moiety .....        C48 Check
PLAT380_ALERT_4_G Incorrectly? Oriented X(sp2)-Methyl Moiety .....        C55 Check
PLAT380_ALERT_4_G Incorrectly? Oriented X(sp2)-Methyl Moiety .....        C56 Check
PLAT380_ALERT_4_G Incorrectly? Oriented X(sp2)-Methyl Moiety .....        C63 Check
PLAT380_ALERT_4_G Incorrectly? Oriented X(sp2)-Methyl Moiety .....        C64 Check
PLAT790_ALERT_4_G Centre of Gravity not Within Unit Cell: Resd.  #          2 Note
              C H2 Cl2
PLAT860_ALERT_3_G Number of Least-Squares Restraints .............         48 Note
PLAT883_ALERT_1_G No Info/Value for _atom_sites_solution_primary .     Please Do !
PLAT941_ALERT_3_G Average HKL Measurement Multiplicity ...........        3.6 Low
PLAT960_ALERT_3_G Number of Intensities with I  <   - 2*sig(I) ...          2 Check
PLAT965_ALERT_2_G The SHELXL WEIGHT Optimisation has not Converged     Please Check
PLAT978_ALERT_2_G Number C-C Bonds with Positive Residual Density.          0 Info
```

---

0 **ALERT level A** = Most likely a serious problem - resolve or explain
0 **ALERT level B** = A potentially serious problem, consider carefully
26 **ALERT level C** = Check. Ensure it is not caused by an omission or oversight
55 **ALERT level G** = General information/check it is not something unexpected

```
11 ALERT type 1 CIF construction/syntax error, inconsistent or missing data
18 ALERT type 2 Indicator that the structure model may be wrong or deficient
 9 ALERT type 3 Indicator that the structure quality may be low
43 ALERT type 4 Improvement, methodology, query or suggestion
 0 ALERT type 5 Informative message, check
```

Publication of your CIF

You should attempt to resolve as many as possible of the alerts in all categories. Often the minor alerts point to easily fixed oversights, errors and omissions in your CIF or refinement strategy, so attention to these fine details can be worthwhile. In order to resolve some of the more serious problems it may be necessary to carry out additional measurements or structure refinements. However, the nature of your study may justify the reported deviations from journal submission requirements and the more serious of these should be commented upon in the discussion or experimental section of a paper or in the "special_details" fields of the CIF. *checkCIF* was carefully designed to identify outliers and unusual parameters, but every test has its limitations and alerts that are not important in a particular case may appear. Conversely, the absence of alerts does not guarantee there are no aspects of the results needing attention. It is up to the individual to critically assess their own results and, if necessary, seek expert advice.

If you wish to submit your CIF for publication in Acta Crystallographica Section C or E, you should upload your CIF via the web. If you wish to submit your CIF for publication in IUCrData you should upload your CIF via the web. If your CIF is to form part of a submission to another IUCr journal, you will be asked, either during electronic submission or by the Co-editor handling your paper, to upload your CIF via our web site.

PLATON version of 03/06/2021; check.def file version of 02/06/2021

Datablock I - ellipsoid plot

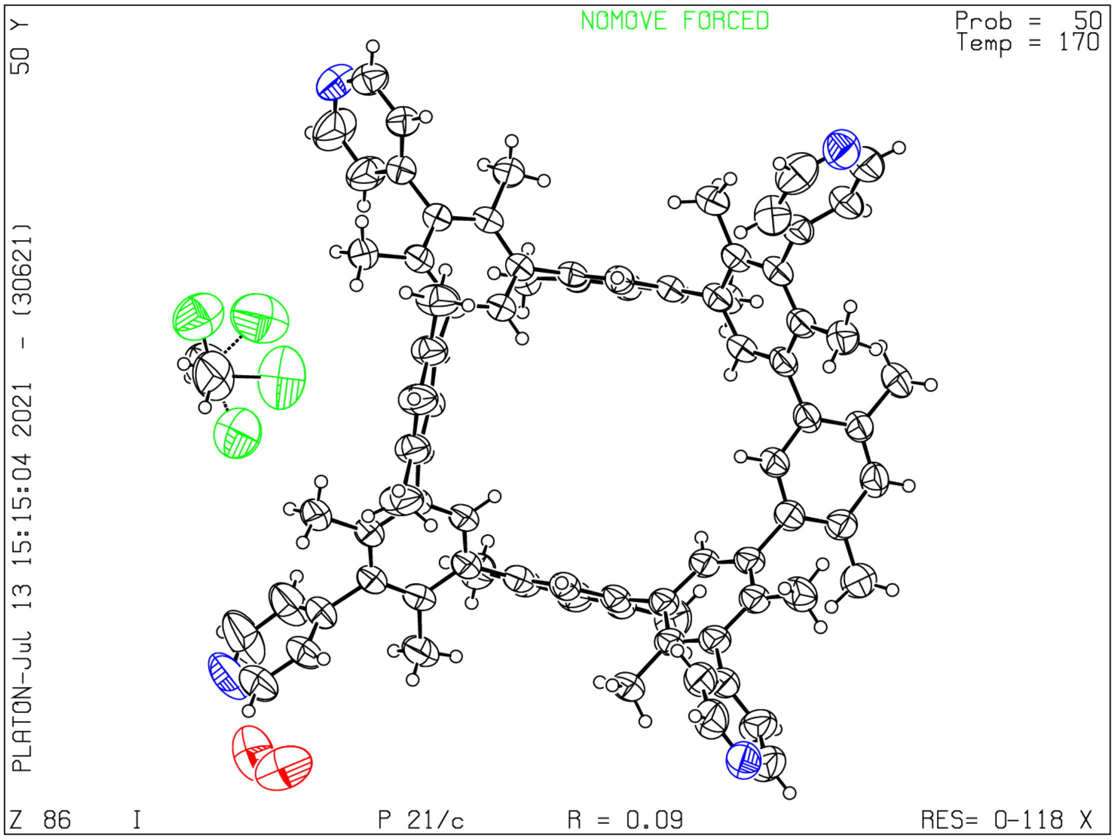

checkCIF/PLATON report

Structure factors have been supplied for datablock(s) I

THIS REPORT IS FOR GUIDANCE ONLY. IF USED AS PART OF A REVIEW PROCEDURE FOR PUBLICATION, IT SHOULD NOT REPLACE THE EXPERTISE OF AN EXPERIENCED CRYSTALLOGRAPHIC REFEREE.

No syntax errors found. CIF dictionary Interpreting this report

Datablock: 3•$CH_3COOC_2H_5$•2CH_3CN•3.5H_2O

```
Bond precision:  C-C = 0.0062 A          Wavelength=1.54184

Cell:          a=15.4676(3)      b=16.1929(3)      c=16.3730(3)
               alpha=90.832(2)   beta=100.813(2)   gamma=91.175(2)
Temperature:   100 K
```

	Calculated	Reported
Volume	4026.50(13)	4026.50(13)
Space group	P -1	P -1
Hall group	-P 1	-P 1
Moiety formula	2(C84 H76 N4), C2 O2, 1.5(C4 O2), C2 N O, 3(C2 N), 5(O)	?
Sum formula	C184 H152 N12 O11	C92 H97 N6 O5.50
Mr	2707.19	1374.75
Dx,g cm-3	1.117	1.134
Z	1	2
Mu (mm-1)	0.548	0.549
F000	1428.0	1470.0
F000’	1432.02	
h,k,lmax	17,18,18	17,18,18
Nref	12846	12820
Tmin,Tmax	0.877,0.947	0.671,1.000
Tmin’	0.848	

```
Correction method= # Reported T Limits: Tmin=0.671 Tmax=1.000
AbsCorr = MULTI-SCAN

Data completeness= 0.998          Theta(max)= 62.499

R(reflections)= 0.1079( 11418)    wR2(reflections)= 0.2400( 12820)

S = 1.020                   Npar= 1055
```

The following ALERTS were generated. Each ALERT has the format
test-name_ALERT_alert-type_alert-level.
Click on the hyperlinks for more details of the test.

Alert level A

PLAT430_ALERT_2_A Short Inter D...A Contact O1 ..O5W . 2.33 Ang.
x,y,z = 1_555 Check

Author Response: Due to the intermolecular hydrogen bonding interactions.

PLAT430_ALERT_2_A Short Inter D...A Contact O1W ..O3W . 1.97 Ang.
x,y,z = 1_555 Check

Author Response: Due to the intermolecular hydrogen bonding interactions.

PLAT430_ALERT_2_A Short Inter D...A Contact O2W ..O3W . 2.05 Ang.
x,y,z = 1_555 Check

Author Response: Due to the intermolecular hydrogen bonding interactions.

Alert level B

PLAT043_ALERT_1_B Calculated and Reported Mol. Weight Differ by .. 42.31 Check

Author Response: Due to the Mol. Weight caculation involving the disorder H atoms.

PLAT097_ALERT_2_B Large Reported Max. (Positive) Residual Density 1.14 eA-3

Author Response: Due to the solvent molecular disorder.

PLAT306_ALERT_2_B Isolated Oxygen Atom (H-atoms Missing ?) .... O1W Check

Author Response: Due to the H disorder of solvent water molecules.

PLAT306_ALERT_2_B Isolated Oxygen Atom (H-atoms Missing ?) .... O2W Check

Author Response: Due to the H disorder of solvent water molecules.

PLAT430_ALERT_2_B Short Inter D...A Contact O1W ..O1W . 2.56 Ang.
1-x,-y,1-z = 2_656 Check

Author Response: Due to the intermolecular hydrogen bonding interactions.

PLAT430_ALERT_2_B Short Inter D...A Contact O1W ..O2W . 2.75 Ang.
x,y,z = 1_555 Check

Author Response: Due to the intermolecular hydrogen bonding interactions.

```
PLAT430_ALERT_2_B Short Inter D...A Contact  O4W      ..N4       .       2.80 Ang.
                                              x,y,-1+z  =      1_554 Check
```

Author Response: Due to the intermolecular hydrogen bonding interactions.

Alert level C

```
ABSTY02_ALERT_1_C  An _exptl_absorpt_correction_type has been given without
            a literature citation. This should be contained in the
            _exptl_absorpt_process_details field.
            Absorption correction given as multi-scan
DIFMN02_ALERT_2_C  The minimum difference density is < -0.1*ZMAX*0.75
            _refine_diff_density_min given =     -0.783
            Test value =     -0.600
DIFMN03_ALERT_1_C  The minimum difference density is < -0.1*ZMAX*0.75
            The relevant atom site should be identified.
DIFMX02_ALERT_1_C  The maximum difference density is > 0.1*ZMAX*0.75
            The relevant atom site should be identified.
THETM01_ALERT_3_C  The value of sine(theta_max)/wavelength is less than 0.590
            Calculated sin(theta_max)/wavelength =    0.5753
PLAT018_ALERT_1_C _diffrn_measured_fraction_theta_max .NE. *_full           ! Check
PLAT041_ALERT_1_C Calc. and Reported SumFormula    Strings  Differ     Please Check
PLAT044_ALERT_1_C Calculated and Reported Density Dx  Differ by ..     0.0175 Check
PLAT048_ALERT_1_C MoietyFormula Not Given (or Incomplete) ........     Please Check
PLAT068_ALERT_1_C Reported F000 Differs from Calcd (or Missing)...     Please Check
PLAT082_ALERT_2_C High R1 Value ..................................       0.11 Report
PLAT098_ALERT_2_C Large Reported Min.  (Negative) Residual Density      -0.78 eA-3
PLAT260_ALERT_2_C Large Average Ueq of Residue Including       O5W      0.128 Check
PLAT260_ALERT_2_C Large Average Ueq of Residue Including       O4W      0.153 Check
PLAT260_ALERT_2_C Large Average Ueq of Residue Including       O1W      0.135 Check
PLAT260_ALERT_2_C Large Average Ueq of Residue Including       O2W      0.171 Check
PLAT260_ALERT_2_C Large Average Ueq of Residue Including       O3W      0.130 Check
PLAT340_ALERT_3_C Low Bond Precision on  C-C Bonds ...............    0.00624 Ang.
PLAT413_ALERT_2_C Short Inter XH3 .. XHn     H8B      ..H48C     .       2.13 Ang.
                                               1+x,y,z  =      1_655 Check
PLAT713_ALERT_1_C TORSION Unknown or Inconsistent Label ..........     C91A_B Check
                    C91A_B      O1A_B       O5W         C93A_B
PLAT906_ALERT_3_C Large K Value in the Analysis of Variance ......      9.353 Check
PLAT906_ALERT_3_C Large K Value in the Analysis of Variance ......      2.186 Check
PLAT911_ALERT_3_C Missing FCF Refl Between Thmin & STh/L=    0.575         23 Report
PLAT918_ALERT_3_C Reflection(s) with I(obs) much Smaller I(calc) .          5 Check
PLAT975_ALERT_2_C Check Calcd Resid. Dens.  0.62A   From O3W         0.88 eA-3
PLAT975_ALERT_2_C Check Calcd Resid. Dens.  0.53A   From O2W         0.55 eA-3
PLAT976_ALERT_2_C Check Calcd Resid. Dens.  0.60A   From O2W        -0.78 eA-3
PLAT976_ALERT_2_C Check Calcd Resid. Dens.  0.98A   From O2W        -0.59 eA-3
PLAT976_ALERT_2_C Check Calcd Resid. Dens.  0.69A   From O2W        -0.41 eA-3
PLAT977_ALERT_2_C Check Negative Difference Density on H7B          -0.33 eA-3
PLAT977_ALERT_2_C Check Negative Difference Density on H7C          -0.31 eA-3
PLAT977_ALERT_2_C Check Negative Difference Density on H15A         -0.31 eA-3
PLAT977_ALERT_2_C Check Negative Difference Density on H15B         -0.45 eA-3
PLAT977_ALERT_2_C Check Negative Difference Density on H15C         -0.33 eA-3
PLAT977_ALERT_2_C Check Negative Difference Density on H23B         -0.32 eA-3
PLAT977_ALERT_2_C Check Negative Difference Density on H39C         -0.37 eA-3
PLAT977_ALERT_2_C Check Negative Difference Density on H47A         -0.33 eA-3
PLAT977_ALERT_2_C Check Negative Difference Density on H47B         -0.33 eA-3
PLAT977_ALERT_2_C Check Negative Difference Density on H47C         -0.34 eA-3
PLAT977_ALERT_2_C Check Negative Difference Density on H48B         -0.37 eA-3
```

Alert level G

```
FORMU01_ALERT_2_G  There is a discrepancy between the atom counts in the
            _chemical_formula_sum and the formula from the _atom_site* data.
            Atom count from _chemical_formula_sum:C92 H97 N6 O5.5
            Atom count from the _atom_site data:  C92 H76 N6 O5.5
CELLZ01_ALERT_1_G Difference between formula and atom_site contents detected.
CELLZ01_ALERT_1_G WARNING: H atoms missing from atom site list. Is this intentional?
           From the CIF: _cell_formula_units_Z    2
           From the CIF: _chemical_formula_sum  C92 H97 N6 O5.50
           TEST: Compare cell contents of formula and atom_site data

           atom    Z*formula  cif sites diff
           C        184.00    184.00    0.00
           H        194.00    152.00   42.00
           N         12.00     12.00    0.00
           O         11.00     11.00    0.00
PLAT002_ALERT_2_G Number of Distance or Angle Restraints on AtSite         24 Note
PLAT003_ALERT_2_G Number of Uiso or Uij Restrained non-H Atoms ...         29 Report
PLAT045_ALERT_1_G Calculated and Reported Z Differ by a Factor ...       0.50 Check
PLAT083_ALERT_2_G SHELXL Second Parameter in WGHT  Unusually Large      18.00 Why ?
PLAT154_ALERT_1_G The s.u.'s on the Cell Angles are Equal ..(Note)      0.002 Degree
PLAT172_ALERT_4_G The CIF-Embedded .res File Contains DFIX Records         32 Report
PLAT177_ALERT_4_G The CIF-Embedded .res File Contains DELU Records          3 Report
PLAT178_ALERT_4_G The CIF-Embedded .res File Contains SIMU Records          3 Report
PLAT186_ALERT_4_G The CIF-Embedded .res File Contains ISOR Records          4 Report
PLAT300_ALERT_4_G Atom Site Occupancy of O5W       Constrained at        0.5 Check
PLAT300_ALERT_4_G Atom Site Occupancy of O1A       Constrained at       0.25 Check
PLAT300_ALERT_4_G Atom Site Occupancy of O2A       Constrained at       0.25 Check
PLAT300_ALERT_4_G Atom Site Occupancy of C90A      Constrained at       0.25 Check
PLAT300_ALERT_4_G Atom Site Occupancy of C91A      Constrained at       0.25 Check
PLAT300_ALERT_4_G Atom Site Occupancy of C92A      Constrained at       0.25 Check
PLAT300_ALERT_4_G Atom Site Occupancy of C93A      Constrained at       0.25 Check
PLAT300_ALERT_4_G Atom Site Occupancy of O1        Constrained at       0.75 Check
PLAT300_ALERT_4_G Atom Site Occupancy of O2        Constrained at       0.75 Check
PLAT300_ALERT_4_G Atom Site Occupancy of C90       Constrained at       0.75 Check
PLAT300_ALERT_4_G Atom Site Occupancy of C91       Constrained at       0.75 Check
PLAT300_ALERT_4_G Atom Site Occupancy of C92       Constrained at       0.75 Check
PLAT300_ALERT_4_G Atom Site Occupancy of C93       Constrained at       0.75 Check
PLAT300_ALERT_4_G Atom Site Occupancy of O4W       Constrained at        0.5 Check
PLAT300_ALERT_4_G Atom Site Occupancy of N6A       Constrained at        0.5 Check
PLAT300_ALERT_4_G Atom Site Occupancy of C88A      Constrained at        0.5 Check
PLAT300_ALERT_4_G Atom Site Occupancy of C89A      Constrained at        0.5 Check
PLAT300_ALERT_4_G Atom Site Occupancy of N5        Constrained at        0.5 Check
PLAT300_ALERT_4_G Atom Site Occupancy of C86       Constrained at        0.5 Check
PLAT300_ALERT_4_G Atom Site Occupancy of C87       Constrained at        0.5 Check
PLAT300_ALERT_4_G Atom Site Occupancy of N5A       Constrained at        0.5 Check
PLAT300_ALERT_4_G Atom Site Occupancy of C86A      Constrained at        0.5 Check
PLAT300_ALERT_4_G Atom Site Occupancy of C87A      Constrained at        0.5 Check
PLAT300_ALERT_4_G Atom Site Occupancy of N6        Constrained at        0.5 Check
PLAT300_ALERT_4_G Atom Site Occupancy of C88       Constrained at        0.5 Check
PLAT300_ALERT_4_G Atom Site Occupancy of C89       Constrained at        0.5 Check
PLAT300_ALERT_4_G Atom Site Occupancy of O3W       Constrained at        0.5 Check
PLAT302_ALERT_4_G Anion/Solvent/Minor-Residue Disorder (Resd  2  )       100% Note
PLAT302_ALERT_4_G Anion/Solvent/Minor-Residue Disorder (Resd  3  )       100% Note
PLAT302_ALERT_4_G Anion/Solvent/Minor-Residue Disorder (Resd  4  )       100% Note
PLAT302_ALERT_4_G Anion/Solvent/Minor-Residue Disorder (Resd  5  )       100% Note
PLAT302_ALERT_4_G Anion/Solvent/Minor-Residue Disorder (Resd  6  )       100% Note
PLAT302_ALERT_4_G Anion/Solvent/Minor-Residue Disorder (Resd  7  )       100% Note
PLAT302_ALERT_4_G Anion/Solvent/Minor-Residue Disorder (Resd 10  )       100% Note
PLAT304_ALERT_4_G Non-Integer Number of Atoms in ..... (Resd  3  )       4.50 Check
PLAT304_ALERT_4_G Non-Integer Number of Atoms in ..... (Resd  5  )       1.50 Check
```

```
PLAT304_ALERT_4_G Non-Integer Number of Atoms in ..... (Resd  6  )       1.50 Check
PLAT304_ALERT_4_G Non-Integer Number of Atoms in ..... (Resd  7  )       1.50 Check
PLAT304_ALERT_4_G Non-Integer Number of Atoms in ..... (Resd 10  )       0.50 Check
PLAT311_ALERT_2_G Isolated Disordered Oxygen Atom (No H's ?) .....        O3W Check
PLAT315_ALERT_2_G Singly Bonded Carbon Detected (H-atoms Missing).        C90 Check
PLAT315_ALERT_2_G Singly Bonded Carbon Detected (H-atoms Missing).        C93 Check
PLAT315_ALERT_2_G Singly Bonded Carbon Detected (H-atoms Missing).        C86 Check
PLAT315_ALERT_2_G Singly Bonded Carbon Detected (H-atoms Missing).       C86A Check
PLAT315_ALERT_2_G Singly Bonded Carbon Detected (H-atoms Missing).        C88 Check
PLAT380_ALERT_4_G Incorrectly? Oriented X(sp2)-Methyl Moiety .....         C7 Check
PLAT380_ALERT_4_G Incorrectly? Oriented X(sp2)-Methyl Moiety .....         C8 Check
PLAT380_ALERT_4_G Incorrectly? Oriented X(sp2)-Methyl Moiety .....        C15 Check
PLAT380_ALERT_4_G Incorrectly? Oriented X(sp2)-Methyl Moiety .....        C16 Check
PLAT380_ALERT_4_G Incorrectly? Oriented X(sp2)-Methyl Moiety .....        C23 Check
PLAT380_ALERT_4_G Incorrectly? Oriented X(sp2)-Methyl Moiety .....        C24 Check
PLAT380_ALERT_4_G Incorrectly? Oriented X(sp2)-Methyl Moiety .....        C31 Check
PLAT380_ALERT_4_G Incorrectly? Oriented X(sp2)-Methyl Moiety .....        C32 Check
PLAT380_ALERT_4_G Incorrectly? Oriented X(sp2)-Methyl Moiety .....        C39 Check
PLAT380_ALERT_4_G Incorrectly? Oriented X(sp2)-Methyl Moiety .....        C40 Check
PLAT380_ALERT_4_G Incorrectly? Oriented X(sp2)-Methyl Moiety .....        C47 Check
PLAT380_ALERT_4_G Incorrectly? Oriented X(sp2)-Methyl Moiety .....        C48 Check
PLAT380_ALERT_4_G Incorrectly? Oriented X(sp2)-Methyl Moiety .....        C55 Check
PLAT380_ALERT_4_G Incorrectly? Oriented X(sp2)-Methyl Moiety .....        C56 Check
PLAT380_ALERT_4_G Incorrectly? Oriented X(sp2)-Methyl Moiety .....        C63 Check
PLAT380_ALERT_4_G Incorrectly? Oriented X(sp2)-Methyl Moiety .....        C64 Check
PLAT395_ALERT_2_G Deviating  X-O-Y    Angle From 120 for O1A            109.7 Degree
PLAT432_ALERT_2_G Short Inter X...Y Contact  O4W      ..C88              2.84 Ang.
                                                      x,y,z  =      1_555 Check
PLAT432_ALERT_2_G Short Inter X...Y Contact  O5W      ..C90              2.65 Ang.
                                                      x,y,z  =      1_555 Check
PLAT432_ALERT_2_G Short Inter X...Y Contact  O5W      ..C91              2.78 Ang.
                                                      x,y,z  =      1_555 Check
PLAT432_ALERT_2_G Short Inter X...Y Contact  N4       ..C88A             2.92 Ang.
                                                    x,y,1+z  =      1_556 Check
PLAT432_ALERT_2_G Short Inter X...Y Contact  C74      ..C92A             3.16 Ang.
                                                  2-x,-y,-z  =      2_755 Check
PLAT773_ALERT_2_G Check long C-C Bond in CIF: C90A     --C92A            2.01 Ang.
PLAT773_ALERT_2_G Check long C-C Bond in CIF: C92A     --C90A            2.01 Ang.
PLAT779_ALERT_4_G Suspect or Irrelevant (Bond) Angle(s) in CIF ...      36.60 Deg.
              O2A  -C91A -O2A     1_555   1_555   2_755 ......     #  326 Check
PLAT779_ALERT_4_G Suspect or Irrelevant (Bond) Angle(s) in CIF ...      34.50 Deg.
              C93A -C92A -C90A    1_555   1_555   2_755 ......     #  330 Check
PLAT860_ALERT_3_G Number of Least-Squares Restraints ............         339 Note
PLAT883_ALERT_1_G No Info/Value for _atom_sites_solution_primary .     Please Do !
PLAT909_ALERT_3_G Percentage of I>2sig(I) Data at Theta(Max) Still        79% Note
PLAT910_ALERT_3_G Missing # of FCF Reflection(s) Below Theta(Min).          3 Note
PLAT933_ALERT_2_G Number of OMIT Records in Embedded .res File ...         20 Note
PLAT941_ALERT_3_G Average HKL Measurement Multiplicity ..........         4.3 Low
PLAT965_ALERT_2_G The SHELXL WEIGHT Optimisation has not Converged     Please Check
PLAT978_ALERT_2_G Number C-C Bonds with Positive Residual Density.          1 Info
PLAT992_ALERT_5_G Repd & Actual _reflns_number_gt Values Differ by          2 Check
```

---

3 **ALERT level A** = Most likely a serious problem - resolve or explain
7 **ALERT level B** = A potentially serious problem, consider carefully
40 **ALERT level C** = Check. Ensure it is not caused by an omission or oversight
92 **ALERT level G** = General information/check it is not something unexpected

15 ALERT type 1 CIF construction/syntax error, inconsistent or missing data

```
55 ALERT type 2 Indicator that the structure model may be wrong or deficient
10 ALERT type 3 Indicator that the structure quality may be low
61 ALERT type 4 Improvement, methodology, query or suggestion
 1 ALERT type 5 Informative message, check
```

Publication of your CIF

You should attempt to resolve as many as possible of the alerts in all categories. Often the minor alerts point to easily fixed oversights, errors and omissions in your CIF or refinement strategy, so attention to these fine details can be worthwhile. In order to resolve some of the more serious problems it may be necessary to carry out additional measurements or structure refinements. However, the nature of your study may justify the reported deviations from journal submission requirements and the more serious of these should be commented upon in the discussion or experimental section of a paper or in the "special_details" fields of the CIF. *checkCIF* was carefully designed to identify outliers and unusual parameters, but every test has its limitations and alerts that are not important in a particular case may appear. Conversely, the absence of alerts does not guarantee there are no aspects of the results needing attention. It is up to the individual to critically assess their own results and, if necessary, seek expert advice.

If you wish to submit your CIF for publication in Acta Crystallographica Section C or E, you should upload your CIF via the web. If you wish to submit your CIF for publication in IUCrData, you should upload your CIF via the web. If your CIF is to form part of a submission to another IUCr journal, you will be asked, either during electronic submission or by the Co-editor handling your paper, to upload your CIF via our web site.

PLATON version of 03/06/2021; check.def file version of 02/06/2021

Datablock I - ellipsoid plot

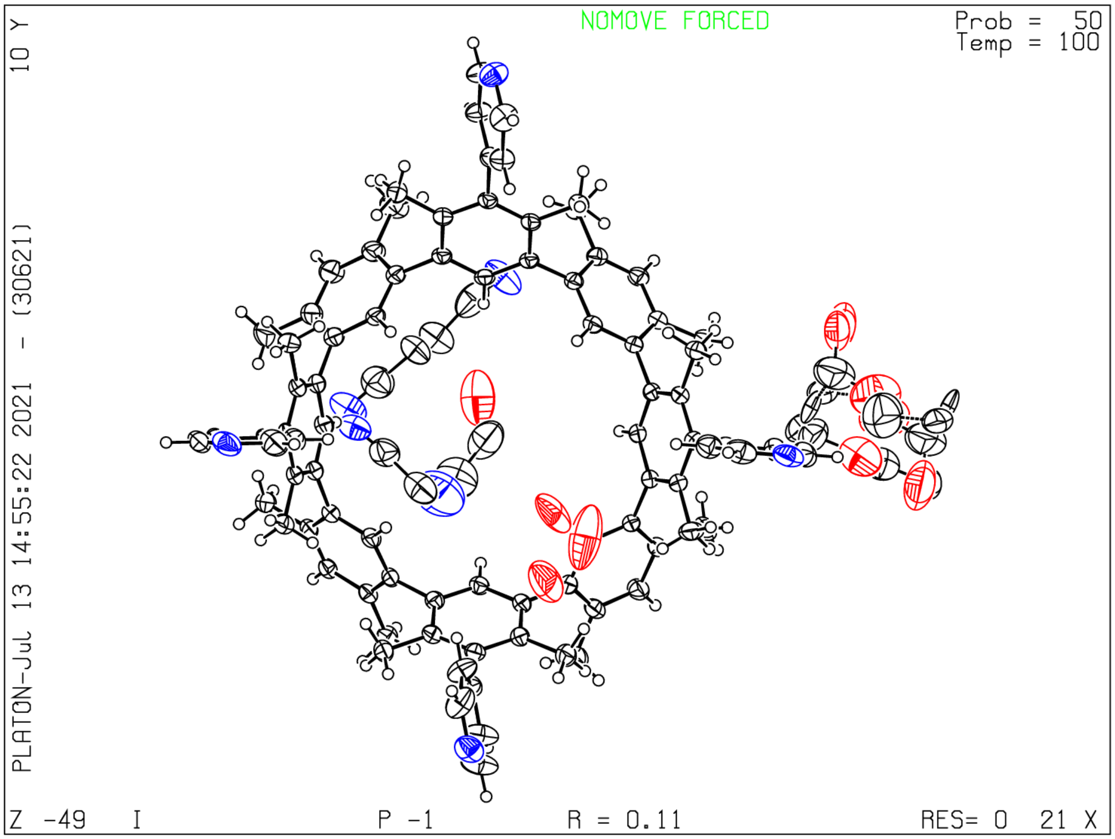

checkCIF/PLATON report

Structure factors have been supplied for datablock(s) I

THIS REPORT IS FOR GUIDANCE ONLY. IF USED AS PART OF A REVIEW PROCEDURE FOR PUBLICATION, IT SHOULD NOT REPLACE THE EXPERTISE OF AN EXPERIENCED CRYSTALLOGRAPHIC REFEREE.

No syntax errors found. CIF dictionary Interpreting this report

Datablock: 4

Bond precision: C-C = 0.0121 A Wavelength=1.54184

Cell:	a=30.115(4)	b=30.115(4)	c=53.271(11)
	alpha=90	beta=90	gamma=90
Temperature:	170 K		

	Calculated	Reported
Volume	48312(16)	48313(17)
Space group	P 42/m n m	P 42/m n m
Hall group	-P 4n 2n	-P 4n 2n
Moiety formula	C504 H456 Cl24 N24 Pd12 [+ solvent]	?
Sum formula	C504 H456 Cl24 N24 Pd12 [+ solvent]	C642 H870 Cl24 N24 O69 Pd12 S69
Mr	8976.56	14367.31
Dx,g cm-3	0.617	0.988
Z	2	2
Mu (mm-1)	2.575	4.133
F000	9216.0	15012.0
F000'	9256.08	
h,k,lmax	34,34,61	32,34,61
Nref	20108	19792
Tmin,Tmax	0.639,0.813	0.496,1.000
Tmin'	0.512	

Correction method= # Reported T Limits: Tmin=0.496 Tmax=1.000
AbsCorr = MULTI-SCAN

Data completeness= 0.984 Theta(max)= 62.496

R(reflections)= 0.0775(6846) wR2(reflections)= 0.2652(19792)

S = 1.004 Npar= 762

The following ALERTS were generated. Each ALERT has the format
test-name_ALERT_alert-type_alert-level.
Click on the hyperlinks for more details of the test.

Alert level B

PLAT026_ALERT_3_B Ratio Observed / Unique Reflections (too) Low .. 35% Check

Author Response: Due to the weak diffraction signals of the sample.

PLAT934_ALERT_3_B Number of (Iobs-Icalc)/Sigma(W) > 10 Outliers .. 2 Check

Author Response: Due to the weak diffraction signals of the sample.

Alert level C

ABSTY02_ALERT_1_C An _exptl_absorpt_correction_type has been given without
a literature citation. This should be contained in the
_exptl_absorpt_process_details field.
Absorption correction given as multi-scan
RINTA01_ALERT_3_C The value of Rint is greater than 0.12
Rint given 0.141
THETM01_ALERT_3_C The value of sine(theta_max)/wavelength is less than 0.590
Calculated sin(theta_max)/wavelength = 0.5753
PLAT018_ALERT_1_C _diffrn_measured_fraction_theta_max .NE. *_full ! Check
PLAT020_ALERT_3_C The Value of Rint is Greater Than 0.12 .... 0.141 Report
PLAT048_ALERT_1_C MoietyFormula Not Given (or Incomplete) .... Please Check
PLAT084_ALERT_3_C High wR2 Value (i.e. > 0.25) .... 0.27 Report
PLAT241_ALERT_2_C High 'MainMol' Ueq as Compared to Neighbors of C1 Check
PLAT241_ALERT_2_C High 'MainMol' Ueq as Compared to Neighbors of C4 Check
PLAT241_ALERT_2_C High 'MainMol' Ueq as Compared to Neighbors of C41 Check
PLAT241_ALERT_2_C High 'MainMol' Ueq as Compared to Neighbors of C44 Check
PLAT241_ALERT_2_C High 'MainMol' Ueq as Compared to Neighbors of C48 Check
PLAT241_ALERT_2_C High 'MainMol' Ueq as Compared to Neighbors of C49 Check
PLAT241_ALERT_2_C High 'MainMol' Ueq as Compared to Neighbors of C50 Check
PLAT241_ALERT_2_C High 'MainMol' Ueq as Compared to Neighbors of C51 Check
PLAT241_ALERT_2_C High 'MainMol' Ueq as Compared to Neighbors of C53 Check
PLAT242_ALERT_2_C Low 'MainMol' Ueq as Compared to Neighbors of Pd3 Check
PLAT242_ALERT_2_C Low 'MainMol' Ueq as Compared to Neighbors of N3 Check
PLAT242_ALERT_2_C Low 'MainMol' Ueq as Compared to Neighbors of N4 Check
PLAT242_ALERT_2_C Low 'MainMol' Ueq as Compared to Neighbors of C2 Check
PLAT242_ALERT_2_C Low 'MainMol' Ueq as Compared to Neighbors of C3 Check
PLAT242_ALERT_2_C Low 'MainMol' Ueq as Compared to Neighbors of C43 Check
PLAT242_ALERT_2_C Low 'MainMol' Ueq as Compared to Neighbors of C47 Check
PLAT242_ALERT_2_C Low 'MainMol' Ueq as Compared to Neighbors of C52 Check
PLAT242_ALERT_2_C Low 'MainMol' Ueq as Compared to Neighbors of C56 Check
PLAT242_ALERT_2_C Low 'MainMol' Ueq as Compared to Neighbors of C70 Check
PLAT260_ALERT_2_C Large Average Ueq of Residue Including Pd1 0.130 Check
PLAT334_ALERT_2_C Small Aver. Benzene C-C Dist C56 -C58 1.37 Ang.
PLAT342_ALERT_3_C Low Bond Precision on C-C Bonds .... 0.01214 Ang.
PLAT369_ALERT_2_C Long C(sp2)-C(sp2) Bond C3 - C6 . 1.53 Ang.
PLAT905_ALERT_3_C Negative K value in the Analysis of Variance ... -27.953 Report
PLAT905_ALERT_3_C Negative K value in the Analysis of Variance ... -2.880 Report
PLAT905_ALERT_3_C Negative K value in the Analysis of Variance ... -0.026 Report
PLAT911_ALERT_3_C Missing FCF Refl Between Thmin & STh/L= 0.575 313 Report

---

Alert level G

```
FORMU01_ALERT_2_G  There is a discrepancy between the atom counts in the
            _chemical_formula_sum and the formula from the _atom_site* data.
            Atom count from _chemical_formula_sum:C642 H870 Cl24 N24 O69 Pd12 S69
            Atom count from the _atom_site data:  C504 H456 Cl24 N24 Pd12
CELLZ01_ALERT_1_G Difference between formula and atom_site contents detected.
CELLZ01_ALERT_1_G ALERT: Large difference may be due to a
            symmetry error - see SYMMG tests
           From the CIF: _cell_formula_units_Z    2
           From the CIF: _chemical_formula_sum  C642 H870 Cl24 N24 O69 Pd12 S69
           TEST: Compare cell contents of formula and atom_site data

           atom    Z*formula  cif sites diff
           C       1284.00   1008.00  276.00
           H       1740.00    912.00  828.00
           Cl        48.00     48.00    0.00
           N         48.00     48.00    0.00
           O        138.00      0.00  138.00
           Pd        24.00     24.00    0.00
           S        138.00      0.00  138.00
PLAT002_ALERT_2_G Number of Distance or Angle Restraints on AtSite         31 Note
PLAT003_ALERT_2_G Number of Uiso or Uij Restrained non-H Atoms ...         28 Report
PLAT014_ALERT_1_G N.O.K.   _shelx_fab_checksum Found in CIF ......     Please Check
PLAT041_ALERT_1_G Calc. and Reported SumFormula    Strings  Differ     Please Check
PLAT051_ALERT_1_G Mu(calc) and Mu(CIF) Ratio Differs from 1.0 by .      37.71 %
PLAT083_ALERT_2_G SHELXL Second Parameter in WGHT  Unusually Large      25.00 Why ?
PLAT168_ALERT_4_G The CIF-Embedded .res File Contains EXYZ Records          2 Report
PLAT171_ALERT_4_G The CIF-Embedded .res File Contains EADP Records          4 Report
PLAT172_ALERT_4_G The CIF-Embedded .res File Contains DFIX Records          7 Report
PLAT177_ALERT_4_G The CIF-Embedded .res File Contains DELU Records          2 Report
PLAT178_ALERT_4_G The CIF-Embedded .res File Contains SIMU Records          2 Report
PLAT186_ALERT_4_G The CIF-Embedded .res File Contains ISOR Records          2 Report
PLAT300_ALERT_4_G Atom Site Occupancy of Cl1        Constrained at        0.5 Check
PLAT300_ALERT_4_G Atom Site Occupancy of Cl2        Constrained at        0.5 Check
PLAT300_ALERT_4_G Atom Site Occupancy of Cl4        Constrained at        0.7 Check
PLAT300_ALERT_4_G Atom Site Occupancy of Cl5        Constrained at       0.67 Check
PLAT300_ALERT_4_G Atom Site Occupancy of Cl6        Constrained at       0.75 Check
PLAT300_ALERT_4_G Atom Site Occupancy of Cl3        Constrained at        0.3 Check
PLAT300_ALERT_4_G Atom Site Occupancy of Cl5A       Constrained at       0.33 Check
PLAT300_ALERT_4_G Atom Site Occupancy of Cl6A       Constrained at       0.25 Check
PLAT300_ALERT_4_G Atom Site Occupancy of C11        Constrained at        0.5 Check
PLAT300_ALERT_4_G Atom Site Occupancy of C11A       Constrained at        0.5 Check
PLAT300_ALERT_4_G Atom Site Occupancy of C12        Constrained at        0.5 Check
PLAT300_ALERT_4_G Atom Site Occupancy of C12A       Constrained at        0.5 Check
PLAT300_ALERT_4_G Atom Site Occupancy of C13        Constrained at        0.5 Check
PLAT300_ALERT_4_G Atom Site Occupancy of C13A       Constrained at        0.5 Check
PLAT300_ALERT_4_G Atom Site Occupancy of C14        Constrained at        0.5 Check
PLAT300_ALERT_4_G Atom Site Occupancy of C14A       Constrained at        0.5 Check
PLAT300_ALERT_4_G Atom Site Occupancy of C15        Constrained at        0.5 Check
PLAT300_ALERT_4_G Atom Site Occupancy of C15A       Constrained at        0.5 Check
PLAT300_ALERT_4_G Atom Site Occupancy of C16        Constrained at        0.5 Check
PLAT300_ALERT_4_G Atom Site Occupancy of C16A       Constrained at        0.5 Check
PLAT300_ALERT_4_G Atom Site Occupancy of C17        Constrained at        0.5 Check
PLAT300_ALERT_4_G Atom Site Occupancy of C17A       Constrained at        0.5 Check
PLAT300_ALERT_4_G Atom Site Occupancy of C18        Constrained at        0.5 Check
PLAT300_ALERT_4_G Atom Site Occupancy of C18A       Constrained at        0.5 Check
PLAT300_ALERT_4_G Atom Site Occupancy of C27        Constrained at        0.5 Check
PLAT300_ALERT_4_G Atom Site Occupancy of C27A       Constrained at        0.5 Check
PLAT300_ALERT_4_G Atom Site Occupancy of C28        Constrained at        0.5 Check
PLAT300_ALERT_4_G Atom Site Occupancy of C28A       Constrained at        0.5 Check
PLAT300_ALERT_4_G Atom Site Occupancy of C29        Constrained at        0.5 Check
```

```
PLAT300_ALERT_4_G Atom Site Occupancy of C29A       Constrained at        0.5 Check
PLAT300_ALERT_4_G Atom Site Occupancy of C30        Constrained at        0.5 Check
PLAT300_ALERT_4_G Atom Site Occupancy of C30A       Constrained at        0.5 Check
PLAT300_ALERT_4_G Atom Site Occupancy of C31        Constrained at        0.5 Check
PLAT300_ALERT_4_G Atom Site Occupancy of C31A       Constrained at        0.5 Check
PLAT300_ALERT_4_G Atom Site Occupancy of C32        Constrained at        0.5 Check
PLAT300_ALERT_4_G Atom Site Occupancy of C32A       Constrained at        0.5 Check
PLAT300_ALERT_4_G Atom Site Occupancy of C33        Constrained at        0.5 Check
PLAT300_ALERT_4_G Atom Site Occupancy of C33A       Constrained at        0.5 Check
PLAT300_ALERT_4_G Atom Site Occupancy of C34        Constrained at        0.5 Check
PLAT300_ALERT_4_G Atom Site Occupancy of C34A       Constrained at        0.5 Check
PLAT300_ALERT_4_G Atom Site Occupancy of H11A       Constrained at        0.5 Check
PLAT300_ALERT_4_G Atom Site Occupancy of H11B       Constrained at        0.5 Check
PLAT300_ALERT_4_G Atom Site Occupancy of H14A       Constrained at        0.5 Check
PLAT300_ALERT_4_G Atom Site Occupancy of H14B       Constrained at        0.5 Check
PLAT300_ALERT_4_G Atom Site Occupancy of H17A       Constrained at        0.5 Check
PLAT300_ALERT_4_G Atom Site Occupancy of H17B       Constrained at        0.5 Check
PLAT300_ALERT_4_G Atom Site Occupancy of H17C       Constrained at        0.5 Check
PLAT300_ALERT_4_G Atom Site Occupancy of H17D       Constrained at        0.5 Check
PLAT300_ALERT_4_G Atom Site Occupancy of H17E       Constrained at        0.5 Check
PLAT300_ALERT_4_G Atom Site Occupancy of H17F       Constrained at        0.5 Check
PLAT300_ALERT_4_G Atom Site Occupancy of H18A       Constrained at        0.5 Check
PLAT300_ALERT_4_G Atom Site Occupancy of H18B       Constrained at        0.5 Check
PLAT300_ALERT_4_G Atom Site Occupancy of H18C       Constrained at        0.5 Check
PLAT300_ALERT_4_G Atom Site Occupancy of H18D       Constrained at        0.5 Check
PLAT300_ALERT_4_G Atom Site Occupancy of H18E       Constrained at        0.5 Check
PLAT300_ALERT_4_G Atom Site Occupancy of H18F       Constrained at        0.5 Check
PLAT300_ALERT_4_G Atom Site Occupancy of H27A       Constrained at        0.5 Check
PLAT300_ALERT_4_G Atom Site Occupancy of H27B       Constrained at        0.5 Check
PLAT300_ALERT_4_G Atom Site Occupancy of H30A       Constrained at        0.5 Check
PLAT300_ALERT_4_G Atom Site Occupancy of H30B       Constrained at        0.5 Check
PLAT300_ALERT_4_G Atom Site Occupancy of H33A       Constrained at        0.5 Check
PLAT300_ALERT_4_G Atom Site Occupancy of H33B       Constrained at        0.5 Check
PLAT300_ALERT_4_G Atom Site Occupancy of H33C       Constrained at        0.5 Check
PLAT300_ALERT_4_G Atom Site Occupancy of H33D       Constrained at        0.5 Check
PLAT300_ALERT_4_G Atom Site Occupancy of H33E       Constrained at        0.5 Check
PLAT300_ALERT_4_G Atom Site Occupancy of H33F       Constrained at        0.5 Check
PLAT300_ALERT_4_G Atom Site Occupancy of H34A       Constrained at        0.5 Check
PLAT300_ALERT_4_G Atom Site Occupancy of H34B       Constrained at        0.5 Check
PLAT300_ALERT_4_G Atom Site Occupancy of H34C       Constrained at        0.5 Check
PLAT300_ALERT_4_G Atom Site Occupancy of H34D       Constrained at        0.5 Check
PLAT300_ALERT_4_G Atom Site Occupancy of H34E       Constrained at        0.5 Check
PLAT300_ALERT_4_G Atom Site Occupancy of H34F       Constrained at        0.5 Check
PLAT301_ALERT_3_G Main Residue  Disorder ..............(Resd  1  )        28% Note
PLAT380_ALERT_4_G Incorrectly? Oriented X(sp2)-Methyl Moiety .....        C10 Check
PLAT380_ALERT_4_G Incorrectly? Oriented X(sp2)-Methyl Moiety .....        C17 Check
PLAT380_ALERT_4_G Incorrectly? Oriented X(sp2)-Methyl Moiety .....       C17A Check
PLAT380_ALERT_4_G Incorrectly? Oriented X(sp2)-Methyl Moiety .....        C18 Check
PLAT380_ALERT_4_G Incorrectly? Oriented X(sp2)-Methyl Moiety .....       C18A Check
PLAT380_ALERT_4_G Incorrectly? Oriented X(sp2)-Methyl Moiety .....        C25 Check
PLAT380_ALERT_4_G Incorrectly? Oriented X(sp2)-Methyl Moiety .....        C26 Check
PLAT380_ALERT_4_G Incorrectly? Oriented X(sp2)-Methyl Moiety .....        C33 Check
PLAT380_ALERT_4_G Incorrectly? Oriented X(sp2)-Methyl Moiety .....       C33A Check
PLAT380_ALERT_4_G Incorrectly? Oriented X(sp2)-Methyl Moiety .....        C34 Check
PLAT380_ALERT_4_G Incorrectly? Oriented X(sp2)-Methyl Moiety .....       C34A Check
PLAT380_ALERT_4_G Incorrectly? Oriented X(sp2)-Methyl Moiety .....        C39 Check
PLAT380_ALERT_4_G Incorrectly? Oriented X(sp2)-Methyl Moiety .....        C55 Check
PLAT380_ALERT_4_G Incorrectly? Oriented X(sp2)-Methyl Moiety .....        C66 Check
PLAT380_ALERT_4_G Incorrectly? Oriented X(sp2)-Methyl Moiety .....        C67 Check
PLAT380_ALERT_4_G Incorrectly? Oriented X(sp2)-Methyl Moiety .....        C72 Check
PLAT412_ALERT_2_G Short Intra XH3 .. XHn     H26C     ..H33C     .       2.10 Ang.
                                                      x,y,z  =      1_555 Check
```

```
PLAT432_ALERT_2_G Short Inter X...Y Contact  Cl2      ..C              3.25 Ang.
                                                1-x,-y,z  =      2_655 Check
PLAT606_ALERT_4_G Solvent Accessible VOID(S) in Structure ........          ! Info
PLAT860_ALERT_3_G Number of Least-Squares Restraints .............        520 Note
PLAT869_ALERT_4_G ALERTS Related to the Use of SQUEEZE Suppressed           ! Info
PLAT883_ALERT_1_G No Info/Value for _atom_sites_solution_primary .     Please Do !
PLAT910_ALERT_3_G Missing # of FCF Reflection(s) Below Theta(Min).          4 Note
PLAT913_ALERT_3_G Missing # of Very Strong Reflections in FCF ....          2 Note
PLAT933_ALERT_2_G Number of OMIT Records in Embedded .res File ...         20 Note
PLAT950_ALERT_5_G Calculated (ThMax) and CIF-Reported Hmax Differ           2 Units
PLAT960_ALERT_3_G Number of Intensities with I  <   - 2*sig(I) ...         35 Check
PLAT965_ALERT_2_G The SHELXL WEIGHT Optimisation has not Converged     Please Check
PLAT978_ALERT_2_G Number C-C Bonds with Positive Residual Density.          0 Info
```

```
  0 ALERT level A = Most likely a serious problem - resolve or explain
  2 ALERT level B = A potentially serious problem, consider carefully
 34 ALERT level C = Check. Ensure it is not caused by an omission or oversight
117 ALERT level G = General information/check it is not something unexpected

  9 ALERT type 1 CIF construction/syntax error, inconsistent or missing data
 31 ALERT type 2 Indicator that the structure model may be wrong or deficient
 16 ALERT type 3 Indicator that the structure quality may be low
 96 ALERT type 4 Improvement, methodology, query or suggestion
  1 ALERT type 5 Informative message, check
```

Publication of your CIF

You should attempt to resolve as many as possible of the alerts in all categories. Often the minor alerts point to easily fixed oversights, errors and omissions in your CIF or refinement strategy, so attention to these fine details can be worthwhile. In order to resolve some of the more serious problems it may be necessary to carry out additional measurements or structure refinements. However, the nature of your study may justify the reported deviations from journal submission requirements and the more serious of these should be commented upon in the discussion or experimental section of a paper or in the "special_details" fields of the CIF. *checkCIF* was carefully designed to identify outliers and unusual parameters, but every test has its limitations and alerts that are not important in a particular case may appear. Conversely, the absence of alerts does not guarantee there are no aspects of the results needing attention. It is up to the individual to critically assess their own results and, if necessary, seek expert advice.

If you wish to submit your CIF for publication in Acta Crystallographica Section C or E, you should upload your CIF via the web. If you wish to submit your CIF for publication in IUCrData you should upload your CIF via the web. If your CIF is to form part of a submission to another IUCr journal, you will be asked, either during electronic submission or by the Co-editor handling your paper, to upload your CIF via our web site.

PLATON version of 13/07/2021; check.def file version of 13/07/2021

Datablock I - ellipsoid plot

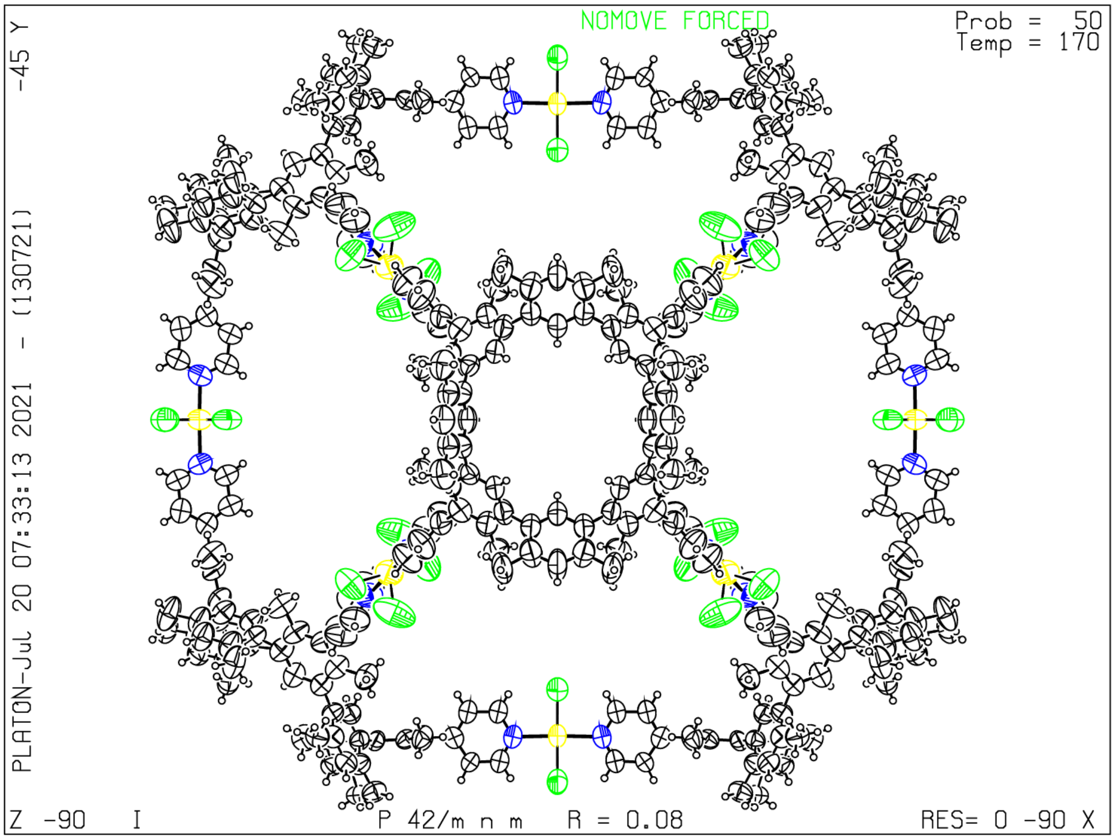

Supplement: Supplementary file 1 — Supplementary Information [file 41467_2023_43756_MOESM1_ESM.pdf]
